# Global, regional, and national time trends in mortality for congenital heart disease, 1990–2019: An age-period-cohort analysis for the Global Burden of Disease 2019 study

**DOI:** 10.1016/j.eclinm.2021.101249

**Published:** 2022-01-11

**Authors:** Zhanhao Su, Zhiyong Zou, Simon I. Hay, Yiwei Liu, Shoujun Li, Huiwen Chen, Mohsen Naghavi, Meghan S. Zimmerman, Gerard R. Martin, Lauren B. Wilner, Craig A. Sable, Christopher J L Murray, Nicholas J. Kassebaum, George C. Patton, Hao Zhang

**Affiliations:** a State Key Laboratory of Cardiovascular Disease, Fuwai Hospital, National Center for Cardiovascular Diseases, Pediatric Cardiac Surgery Center, Fuwai Hospital, Chinese Academy of Medical Sciences and Peking Union Medical College, Beijing, China; b Institute of Child and Adolescent Health, National Health Commission Key Laboratory of Reproductive Health, Peking University School of Public Health, No.38 Xueyuan Rd, Haidian District, Beijing 100191, China; c Institute for Health Metrics and Evaluation, University of Washington, Seattle, WA, United States; d Department of Health Metrics Sciences, School of Medicine, University of Washington, Seattle, WA, United States; e Heart Center and Shanghai Institute of Pediatric Congenital Heart Disease, Shanghai Children's Medical Center, National Children's Medical Center, Shanghai Jiaotong University School of Medicine, Room 7016, Heart centre, Shanghai Children's Medical centre, No. 1678, Dongfang Rd, Pudong District, Shanghai, China; f Division of Pediatric Cardiology, Dartmouth-Hitchcock Medical Center, Lebanon, NH, United States; g Milken Institute School of Public Health, George Washington University, Washington, DC, United States; h Department of Cardiology, Children's National Health System, Washington, DC, United States; i Department of Anesthesiology and Pain Medicine, Harborview Medical Center, University of Washington, Seattle, WA, United States; j Department of Pediatrics, The University of Melbourne, Parkville, Victoria, Australia; k Centre for Adolescent Health, Royal Children's Hospital, Parkville, Victoria, Australia; l Murdoch Children's Research Institute, Melbourne, Victoria, Australia

**Keywords:** congenital heart disease, mortality, age-period-cohort, health disparities

## Abstract

**Background:**

Congenital heart disease (CHD) is the leading cause of morbidity and mortality from birth defects worldwide. We report an overview of trends in CHD mortality in 204 countries and territories over the past 30 years and associations with age, period, and birth cohort.

**Methods:**

Cause-specific CHD mortality estimates were derived from the Global Burden of Disease 2019 study. We utilised an age-period-cohort model to estimate overall annual percentage changes in mortality (net drifts), annual percentage changes from 0 to 4 to 65–69 years (local drifts), period and cohort relative risks (period/cohort effects) between 1990 and 2019. This approach allows for the examination and differentiation of age, period, and cohort effects in the mortality trends, with the potential to identify disparities and treatment gaps in cardiac care.

**Findings:**

CHD is the leading cause of deaths from non-communicable diseases (NCDs) in those under 20 years. Global CHD deaths in 2019 were 217,000 (95% uncertainty interval 177,000–262,000). There were 129 countries with at least 50 deaths. India, China, Pakistan, and Nigeria had the highest mortality, accounting for 39.7% of deaths globally. Between 1990 and 2019, the net drift of CHD mortality ranged from –2.41% per year (95% confidence interval [CI] –2.55, –2.67) in high Socio-demographic Index (SDI) countries to –0.62% per year (95% CI: –0.82, –0.42) in low-SDI countries. Globally, there was an emerging transition in the age distribution of deaths from paediatric to adult populations, except for an increasing trend of mortality in those aged 10–34 years in Mexico and Pakistan. During the past 30 years, favourable mortality reductions were generally found in most high-SDI countries like South Korea (net drift = –4.0% [95% CI –4.8 to –3.1] per year) and the United States (–2.3% [–2.5 to –2.0]), and also in many middle-SDI countries like Brazil (–2.7% [–3.1 to 2.4]) and South Africa (–2.5% [–3.2 to –1.8]). However, 52 of 129 countries had either increasing trends (net drifts ≥0.0%) or stagnated reductions (≥–0.5%) in mortality. The relative risk of mortality generally showed improving trends over time and in successively younger birth cohorts amongst high- and high-middle-SDI countries, with the exceptions of Saudi Arabia and Kazakhstan. 14 middle-SDI countries such as Ecuador and Mexico, and 16 low-middle-SDI countries including India and 20 low-SDI countries including Pakistan, had unfavourable or worsening risks for recent periods and birth cohorts.

**Interpretation:**

CHD mortality is a useful and accessible indicator of trends in the provision of congenital cardiac care both in early childhood and across later life. Improvements in the treatment of CHD should reduce the risk for successively younger cohorts and shift the risk for all age groups over time. Although there were gains in CHD mortality globally over the past three decades, unfavourable period and cohort effects were found in many countries, raising questions about adequacy of their health care for CHD patients across all age groups. These failings carry significant implications for the likelihood of achieving the Sustainable Development Goal targets for under-5 years and NCD mortality.

**Funding:**

Supported by the 10.13039/501100001809National Natural Science Foundation of China (81525002, 31971048, 82073573 to ZZ and HZ), Shanghai Outstanding Medical Academic Leader program (2019LJ22 to HZ), and Collaborative Innovation Program of Shanghai Municipal Health Commission (2020CXJQ01 to HZ), the Bill & Melinda Gates Foundation for the Global Burden of Disease Project (to NJK) and NHMRC fellowship administered through the University of Melbourne (to GCP).

**Research in context**

**Evidence before this study**

We undertook a literature search in PubMed for English-language papers, with no restrictions on publication date. We used the terms “congenital heart disease”, “congenital heart defect”, congenital heart malformation”, “congenital heart anomalies”, “cardiovascular malformation”, “cardiovascular defect”, “cardiovascular anomalies”, “mortality”, “death”, and “trend”. Previous studies have used national databases, registry data, or single-centre cohort data to characterise trends in congenital heart disease (CHD) mortality. Many were from high-income countries, with few reports from the remaining parts of the world. Many countries lack resources to track progress in congenital cardiac care, and no previous studies have examined age, period, and birth cohort trends in CHD mortality using all the available data on a global scale.

**Added value of this study**

We used data from the Global Burden of Disease study 2019 to examine the age, period, and cohort trends in CHD mortality across 204 countries and territories. Despite an overall declining trend in CHD mortality globally, CHD continues to be the leading cause of deaths from non-communicable diseases (NCDs) in populations under 20 years, and there are widening disparities between countries, with mortality gains not necessarily commensurate with the SDI as an index of socioeconomic development. Unfavourable period and cohort effects were most notable in countries in Africa, Asia, and Latin America, consistent with a recent slowing of progress in reducing CHD mortality. The use of age-period-cohort models with GBD data identifies treatment gaps for congenital heart diseases, notably in recent birth cohorts in India, and worsening mortality in adolescents and adults in Mexico and Pakistan, and amongst older adults in China.

**Implications of all the available evidence**

The available resources for managing this patient group are inadequate in many countries that could afford better health care. CHD mortality is a useful indicator of trends in the provision and accessibility of congenital cardiac care in both early childhood and later life. Increasingly, countries need to extend CHD health care beyond paediatric settings to those who increasingly survive into adulthood. To meet the 2030 Sustainable Development Goals of reducing premature deaths from NCDs, continuous global investments in congenital cardiac care are needed to improve outcomes across the life span. Such measures would reduce the risk for successively younger generations and shift risks for all age groups. The methods adopted in this paper may be relevant for tracking the progress of other NCDs that are amenable to early intervention and active treatment.

Alt-text: Unlabelled box

## Introduction

Congenital heart disease (CHD) is the leading group of birth defects, with 13.3 million patients worldwide in 2019,^1^ and an important cause of non-communicable diseases (NCDs).^2^ In 2015, the United Nations adopted the Sustainable Development Goals (SDGs) to end avertable deaths in children under 5 years of age and reduce premature deaths from NCDs by one-third by 2030.^3^ CHD is particularly relevant as this group of conditions accounted for the largest proportion of NCD-related deaths in populations under 30 years of age in 2016.^4^ Concerns have been raised that CHD-related mortality in all ages has been neglected in the global health agenda, limiting the scope to realise the relevant SDG targets.^5^

Over the past three decades, progress in interventional cardiology and congenital heart surgery has substantially reduced mortality for the entire spectrum of CHD.^6^ The global crude mortality rate for CHD declined from 7.1 per 100,000 in 1990 to 2.8 per 100,000 in 2019.^1^ In order to generate information for tracking progress and identifying priorities for investment, an in-depth analysis of time trends in CHD mortality for all countries is warranted. In the study of time trends for disease, the time variable usually acts as a surrogate for causal factors that are changing over time. For patients with CHD, mortality risks can be decomposed into age, period, and birth cohort effects. The risk of death from CHD differs not only by physiological age (the *age effect*) but is likely to differ between birth cohorts with the introduction of new methods of diagnosis and treatment. The diagnosis and correction of heart defects at an early-life stage has long-lasting impact on life outcomes (the *cohort effect*).^7^ Technological advances or health policies related to the management of CHD can also affect all individuals regardless of age and birth cohort in a certain period (the *period effect*). In this regard, analysis of mortality trends with a particular focus on their associations with age, period, and cohort effects has the potential to delineate the success of different aspects of health-care delivery and identify remaining treatment gaps.^7^^,^^8^ For example, a previous study from the UK analysed age, period, and birth cohort trends in CHD mortality amongst children to determine the progress made in prenatal detection, paediatric cardiac surgery, and neonatal care over the period of 1959–2009.^7^

The available literature from high-income countries suggests declining trends of CHD mortality worldwide.^9^^,^^10^ However, these analyses did not differentiate the relative contributions of age, period, and cohort effects on mortality. Many countries around the world, particularly low- and middle-income countries, lack information on mortality trends for CHD or its relationship to age, changes over time, or birth cohorts. The Global Burden of Diseases, Injuries, and Risk Factors Study (GBD) is an international collaboration that uses consistent methodology and all available population-level data to generate population health metrics, offering a unique opportunity to analyse disease trends on a global scale. In this article, we used GBD 2019 data and age-period-cohort models to explore changes in CHD mortality across 204 countries and territories from 1990 to 2019. This manuscript was produced as part of the GBD Collaborator Network and in accordance with GBD Protocols.

## Methods

### Data sources

GBD 2019 provides the most up-to-date estimation of the descriptive epidemiological data on a total of 369 diseases and injuries for 204 countries and territories from 1990 to 2019.^11^^,^^12^ Each death in GBD was assigned to a single underlying cause from a mutually exclusive and collectively exhaustive list of diseases and injuries.^12^ Cause-specific deaths attributed to CHD were mapped to the GBD cause list with the following International Classification of Diseases and Injuries (ICD) codes: Q20-Q28.9 (ICD-10) and 745–747.9 (ICD-9).^1^^,^^2^ Details of data inputs, processing, synthesis, and final models are available in the accompanying GBD 2019 publications.^11^ The GBD network utilised standardised tools within a Bayesian framework to leverage all available data across time, age, and geography, as well as across causes and domains of health to generate disease estimates, allowing for the “borrowing” of information from the available data to produce estimates for countries without primary data sources, and this process allows for estimates of CHD burden in all regions of the globe. All disease estimates from GBD contain 95% uncertainty intervals (UI) for every metric, which are based on the 25th and 975th ordered values of 1000 draws of the posterior distribution.^11^ Countries with fewer or no data sources generally have larger 95% UI, suggesting greater inaccuracy in disease estimates. Notably, many countries around the world lack primary data for CHD mortality, particularly those in Africa.^2^ Therefore, mortality estimates for these countries consist of modelling results from the CHD-specific model informed by data from other countries. The GBD study uses deidentified data, and a waiver of informed consent was approved by the University of Washington Institutional Review Board.

This analysis used the Socio-demographic Index (SDI) for each country, an indicator estimated as a composite of income per capita, average years of schooling, and fertility rate in females under 25 years old.^11^ The SDI is scaled from 0 to 1, with higher values indicating higher socioeconomic levels. All countries were categorised into one of five SDI quintiles based on 2019 SDI values.

### Analysis of overall temporal trends in CHD mortality

Temporal trends in mortality over the study period were assessed by all-age mortality (crude mortality) and age-standardised mortality, and the relative change of mortality in percentage between 1990 and 2019. Age-standardised mortality was calculated using global age-standard population data from GBD 2019.^12^ We also examined the age distribution of deaths as an indirect indicator of survival by arranging death counts into six age strata (neonatal, post-neonatal, 1–4, 5–19, 20–39, 40–69) and calculating the proportions of deaths from each age stratum.

### Age-period-cohort modelling analysis of mortality data

This study uses an age-period-cohort (APC) model framework to analyse the underlying trends in mortality by age, period, and birth cohort.^13^ The APC model is designed to unpack the contributions of age-associated biological factors, and technological and social factors on disease trends, extending beyond traditional epidemiological analyses.^14^ This approach has been adopted in descriptive epidemiology for certain chronic diseases, including cardiovascular diseases.^15^ Generally, the APC model fits a log-linear Poisson model over a Lexis diagram of observed rates and quantifies the additive effects of age, period, and birth cohorts. As the relationship between age, period, and cohort is perfectly linear (birth cohort = period – age), it is statistically impossible to estimate their independent effects, the so-called identification problem.^13^^,^^14^ In this study, we circumvent this issue by producing estimable APC parameters and functions without imposing arbitrary constraints on model parameters.^13^ The APC model is implemented using freely available R tools, and the methodological details are described in previous literature.^16^

GBD 2019 mortality estimates for CHD and population data of each country/region were used as data inputs for the APC model. In a typical APC model, the age and period intervals must all be equal, ie, five-year age groups should be used with five-year calendar periods. As GBD estimates are produced in an unequally spaced data format (five-year age groups with annual data), we arranged GBD data into a single unit framework by selecting the death and population counts from the mid-year of six five-year-periods (ie, [1992] 1990–1994, [1997] 1995–1999 … [2017] 2015–2019) to represent for the specific period. The input data included 14 age groups (from 0 to 4 to 65–69 in five-year age group intervals) and 19 partially overlapping ten-year birth cohorts, as referenced by the mid-year of birth, from 1921 to 1929 (the 1925 cohort) to 2011–2019 (the 2015 cohort) (a detailed schematic is in appendix Table S1). The fitted APC model estimated the overall temporal trend in mortality, which is expressed as the *annual percentage change of mortality* (ie, the *net drift* of mortality,% per year). Technically, the net drift is determined by two components: the component of the trend attributable to calendar time and the component of the trend attributable to the successive cohorts. The APC model also estimated the temporal trend of mortality within each age group, expressed as *annual percentage change of age-specific mortality* (ie, the *local drift* of mortality,% per year), and it reflects trends in birth cohort effects.^16^ A drift of ±1% per year or more is considered a substantial change in mortality^16^ because this approximates ±10%, ±18%, and ±26% of change in the fitted rate over a period of 10, 20, and 30 years. The significance of trends in annual percentage change was tested with a Wald chi-squared test.^16^ The APC model outputs also include fitted longitudinal age-specific rates in the referent cohort adjusted for period deviations to represent age-associated natural history (ie, age effects), and period (cohort) relative risks of mortality for each period (cohort) to represent period (cohort) effects.^16^ The relative risk is computed as the ratio of age-specific rates in each period (cohort) relative to reference period (cohort). Both the period (cohort) rate ratio curves incorporate the entire value of the net drift. The choice of referent period (cohort) is arbitrary and does not affect the interpretation of results. Statistical tests were two-sided and *p* < 0.05 is considered significant. All analysis was conducted in R (version 3.6.3).^17^

### Role of the funding source

The funders of this study had no role in study design, data collection, data analysis, data interpretation, and writing of the manuscript.

## Results

### The leading cause of global mortality from NCDs under 20, 1990–2019

**Figure 1:**
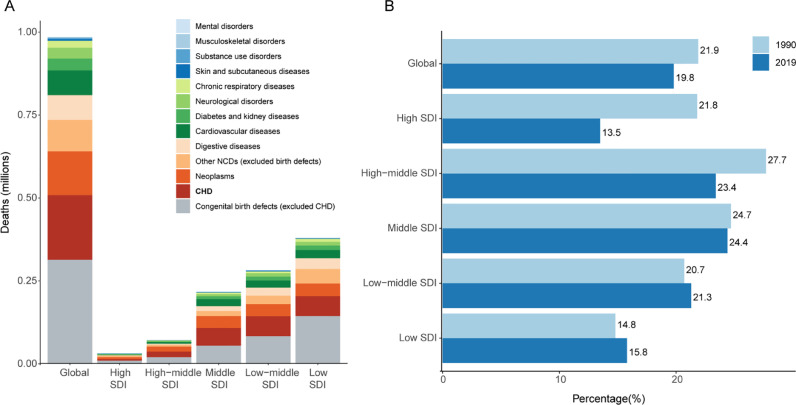
CHD is the leading cause of global mortality from NCDs in the population under 20 years of age (A) The number of deaths in 2019 for NCDs in the population under 20 years. Globally, congenital birth defects are the leading cause of NCDs in this age group, and congenital heart disease (CHD) accounts for the largest proportion of all birth defects. (B) Change in the proportion of deaths from CHD relative to all NCD mortality in the population under 20 years, 1990–2019. CHD=congenital heart disease; NCDs=non-communicable diseases.

Globally in 2019, the estimated number of deaths from NCDs in the population under 20 years of age was 985 thousand (95% UI 837–1171 thousand, Fig. 1A). Of all NCD causes congenital birth defects accounted for over 50% of deaths in those under 20 years of age (509 thousand [406–656 thousand]). CHD is also the leading birth defect with the highest burden of mortality (195 thousand [156–241 thousand]).

Globally across 1990–2019, the proportion of CHD-related deaths relative to all causes of NCD mortality in those under 20 years of age decreased from 21.9% to 19.8%, with the largest relative decrease in high-SDI regions (Fig. 1B). Progress was less evident in high-middle-SDI and middle-SDI regions, and in lower-SDI regions, CHD contributes to an increasing proportion of NCD mortality.

### Global and regional trends in CHD mortality, 1990–2019

**Figure 2:**
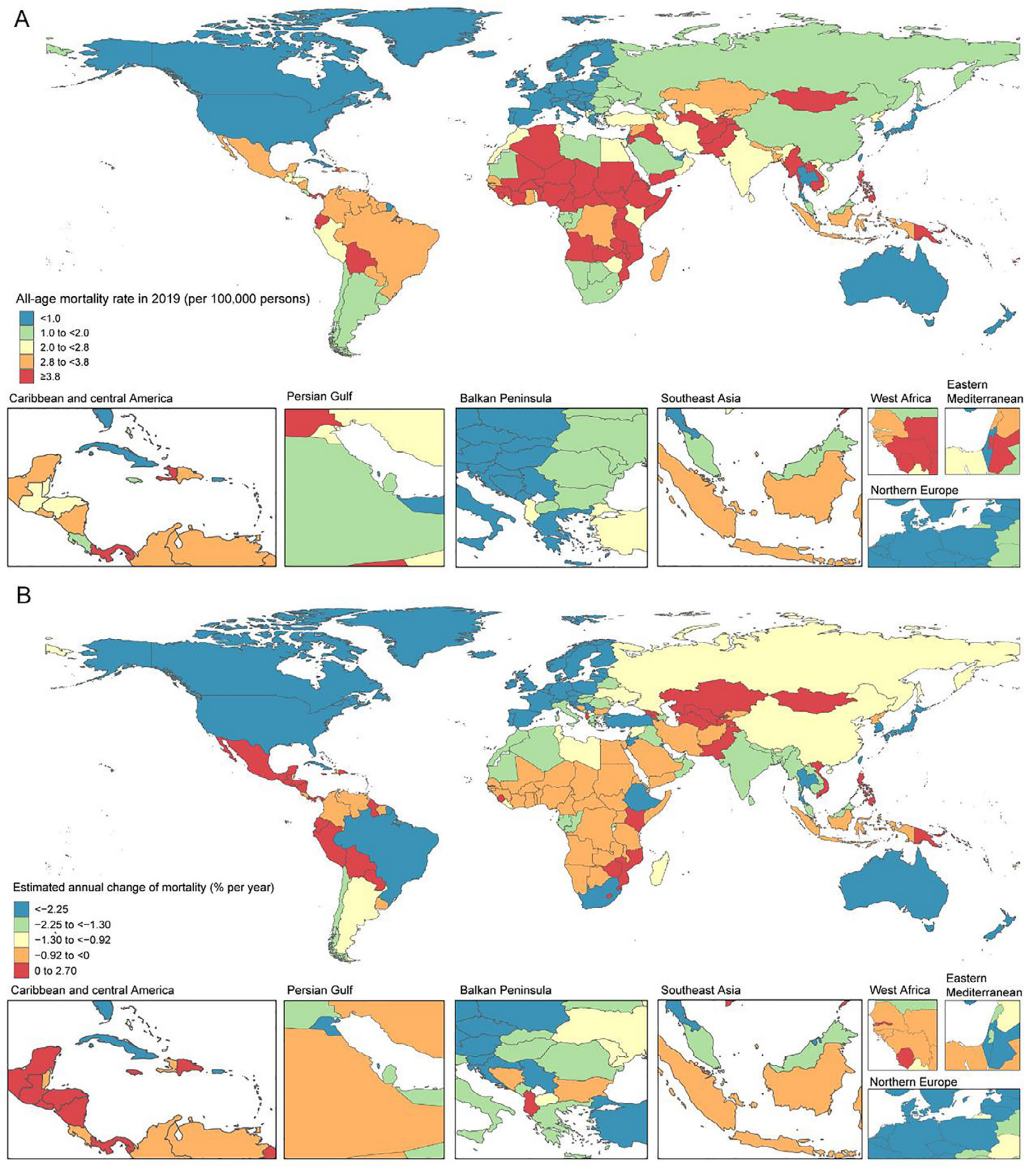
The all-age mortality in 2019 (A) and net drift of mortality during 1990–2019 (B) for CHD in 204 countries and territories (A) World map of all-age mortality for CHD. In 2019, the global all-age mortality rate was 2.80 (95% UI 2.29–3.38) per 100,000 population. (B) World map of net drifts for CHD mortality, ie, estimated annual percentage change of mortality from age-period-cohort model. Net drift captures components of the trends attributable to calendar time and successive birth cohorts. The global net drift of CHD mortality was –1.30% [95% CI –1.38 to –1.21]. CHD=congenital heart disease

Table 1, Fig. 2, appendix Table S2 and Fig. S1 show population, total number of deaths, all-age mortality, and age-standardised mortality, as well as *net drift* of mortality (estimated from the APC model and analogous to annual percentage change of mortality, but capturing both components of the trend attributable to calendar time and successive birth cohorts.^16^) In the past 30 years, although global population increased from 5.3 billion (95% UI 5.2–5.5) to 7.7 billion (7.5–8.0), growth of 44.6%, the number of CHD deaths decreased from 378 thousand (268–563 thousand) to 217 thousand (177–262 thousand), a 42.7% decline. Globally in 2019, all-age mortality for CHD was 2.80 (2.29–3.38) per 100,000 population with a decrease of 60.4% (41.4–71.9), and the age-standardised mortality for CHD was 3.23 (2.64–3.92) per 100,000 population, a 45.5% (19.5–61.1) decrease from 1990. Of note, all-age mortality rates were generally lower than age-standardised rates across SDI regions except low-SDI regions, indicating that all-age mortality rates are more appropriate to capture the true burden of CHD mortality for lower-income countries. The relative reduction in all-age mortality was largest in high-SDI (–71.3% [–74.7 to –64.6]) and high-middle-SDI regions (–74.9% [–82.2 to –65.1]) and lowest in low-SDI regions (–42.1% [–61.9 to 12.1]). The percentage change in number of deaths also decreased in all SDI regions except in low-SDI regions, where there was an increase of 23.7% (–18.7 to 139.5). Globally, the APC model estimated a net drift of CHD mortality at –1.3% (95% CI –1.38 to –1.21) per year, ranging from –2.41% (–2.55 to –2.67) in high-SDI regions to –0.62% (–0.82 to –0.42) in low-SDI regions. A total of 49 countries had net drifts with upper bound of 95% CI <–1.0%. During 1990–2019, low-middle-SDI and low-SDI regions accounted for an increasing proportion of CHD deaths around the world.

**Table 1:** Trends in congenital heart disease mortality across Socio-demographic Index quintiles, 1990–2019.

	Global (N=204)		High SDI (N=41)		High-middle SDI (N=41)		Middle SDI (N=40)		Low-middle SDI (N=41)		Low SDI (N=41)	
	1990	2019	1990	2019	1990	2019	1990	2019	1990	2019	1990	2019
**Population**												
Number, n × 1,000,000	5350 (5239, 5460)	7737 (7483, 7993)	822	1013	1150	1430	1717	2397	1130	1764	528	1128
Percentage of global, %	100	100	15.4	13.1	21.5	18.5	32.1	39.6	21.10	22.8	9.9	14.6
**Deaths**												
Number⁎, n × 1,000	378 (268, 563)	217 (177, 262)	20 (17, 22)	7 (6, 9)	68 (55, 85)	21 (18, 25)	140 (106, 200)	60 (50, 75)	100 (64, 162)	66 (50, 86)	50 (19, 104)	62 (38, 98)
Percentage of global, %	100	100	5.3	3.3	17.9	9.7	37.1	27.9	26.4	30.3	13.3	28.8
Percent change of deaths 1990–2019, %	-42.7 (-59.3, -15.2)		-64.6 (-68.8, -56.3)		-68.8 (-77.8, -56.6)		-56.9 (-73.8, -33.3)		-34.1 (-59.4, 19.5)		23.7 (-18.7, 139.5)	
**APC model estimates**												
Net drift of mortality†, % per year	-1.30 (-1.38, -1.21)		-2.41 (-2.55, -2.67)		-1.65 (-1.78, -1.53)		-1.02 (-1.15, -0.89)		-0.94 (-1.06, -0.81)		-0.62 (-0.82, -0.42)	
**All-age mortality rate**												
Rate per 100,000	7.07 (5.01, 10.52)	2.80 (2.29, 3.38)	2.42 (2.08, 2.63)	0.70 (0.61, 0.84)	5.88 (4.77, 7.36)	1.48 (1.26, 1.72)	8.18 (6.16, 11.65)	2.52 (2.07, 3.11)	8.82 (5.68, 14.32)	3.73 (2.81, 4.85)	9.54 (3.59, 19.61)	5.52 (3.41, 8.68)
Percent change of rate 1990–2019, %	-60.4 (-71.9, -41.4)		-71.3 (-74.7, -64.6)		-74.9 (-82.2, -65.1)		-69.2 (-81.3, -52.2)		-57.8 (-74.0, -23.5)		-42.1 (-61.9, 12.1)	
**Age-standardised mortality rate**												
Rate per 100,000	5.93 (4.22, 8.79)	3.23 (2.64, 3.92)	3.19 (2.74, 3.49)	1.07 (0.93, 1.33)	6.56 (5.31, 8.22)	2.39 (2.00, 2.83)	6.87 (5.18, 9.76)	3.26 (2.66, 4.05)	5.89 (3.84, 9.44)	3.80 (2.86, 4.95)	5.03 (2.00, 10.16)	3.67 (2.32, 5.68)
Percent change of rate 1990–2019, %	-45.5 (-61.1, -19.5)		-66.4 (-71.3, -56.5)		-63.5 (-74.7, -48.8)		-52.5 (-71.1, -26.4)		-35.6 (-59.9, 15.4)		-27.0 (-51.9, 37.3)	

**Notes:** All-age mortality=crude mortality rate.

Age-standardised mortality rate is computed by direct standardisation with global standard population in GBD 2019.

† Net drifts are estimates derived from the age-period-cohort model and denotes overall annual percentage change in mortality, which captures the contribution of the effects from calendar time and successive birth cohorts

⁎ Parentheses for all GBD health estimate indicate 95% uncertainty intervals; parentheses for net drift indicate 95% confidence intervals. SDI=Socio-demographic Index; APC=age-period-cohort.

### National trends in CHD mortality, 1990–2019

Amongst 204 countries and territories, 129 had at least 50 deaths in 2019, with India (death number = 38 thousand, [95% UI 25–56 thousand]), China (25 thousand, [21–30 thousand]), Pakistan (11 thousand, [7–19 thousand]), and Nigeria (11 thousand, [4–25 thousand]) being the top four countries and accounting for 39.7% of CHD deaths globally. Of these 129 countries, 52 showed an increasing trend (net drifts ≥0.0% per year) or modest reduction (–0.5 to 0.0%) in mortality. Tajikistan had the highest increase in all-age mortality (from 4.87 [3.54–7.99] to 12.33 [4.34–18.06] per 100,000), with a net drift of mortality at 2.15% (0.12–4.21) per year. In 2019, 23 countries had an all-age mortality more than two-fold higher than the global average, and seven countries (Haiti, Afghanistan, Tajikistan, Sudan, Yemen, Papua New Guinea, Myanmar) had an age-standardised mortality more than two-fold higher than the global average, most of which were lower-SDI countries in Africa, Asia, and Latin America. Nigeria and Pakistan are examples of highly populous lower-SDI countries showing dramatic population growth (138.2% and 98.6%, respectively) with notable increases in the number of CHD deaths (85.3% [23.3–259.7] and 40.5% [–17.3 to 152.6]). Although favourable mortality reduction was generally observed in higher-SDI countries in Europe, North America, and the Asia-Pacific region, Saudi Arabia had an exceptionally high mortality in 2019 (1.79 per 100,000 [1.33–2.39]), with a relatively flat net drift in mortality at –0.42% (–1.05 to 0.21) per year. In addition, decreasing net drifts were observed in some emerging economies like Brazil, which stood out for its notable reduction in all-age mortality (a 77.3% [48.8–85.5] decrease from 14.12 [6.56–19.78] to 3.20 [2.47–3.97] per 100,000) and net drift of mortality at –2.72% (–3.05 to –2.39) per year. India and China had the highest number of CHD deaths owing to their large populations, and their all-age mortality decreased by 55.4% (17.0–75.2) and 78.8% (69.9–86.3), respectively, with relatively modest net drifts of mortality (India: –1.36% [–1.75 to –0.98]; China: –0.99% [–1.22 to –0.76]). In Mexico, all-age mortality and age-standardised mortality decreased by 49.9% (22.3–80.9) and 20.2% (–23.7 to 69.2), respectively, but the net drift in mortality was essentially flat at 0.12% (–0.29 to 0.52) per year. Collectively, these results suggest that CHD mortality trends were uneven between countries and mortality gains were not necessarily commensurate with expectations based on national-level SDI status (like Saudi Arabia and Mexico). Moreover, the direction of change in mortality as indicated by conventional metrics (all-age/age-standardised mortality) may not fully accord with the change indicated by the net drift derived from the APC model, suggesting the necessity of differentiating period and cohort trends in CHD mortality.

### Time trends in CHD mortality across different age groups

**Figure 3:**
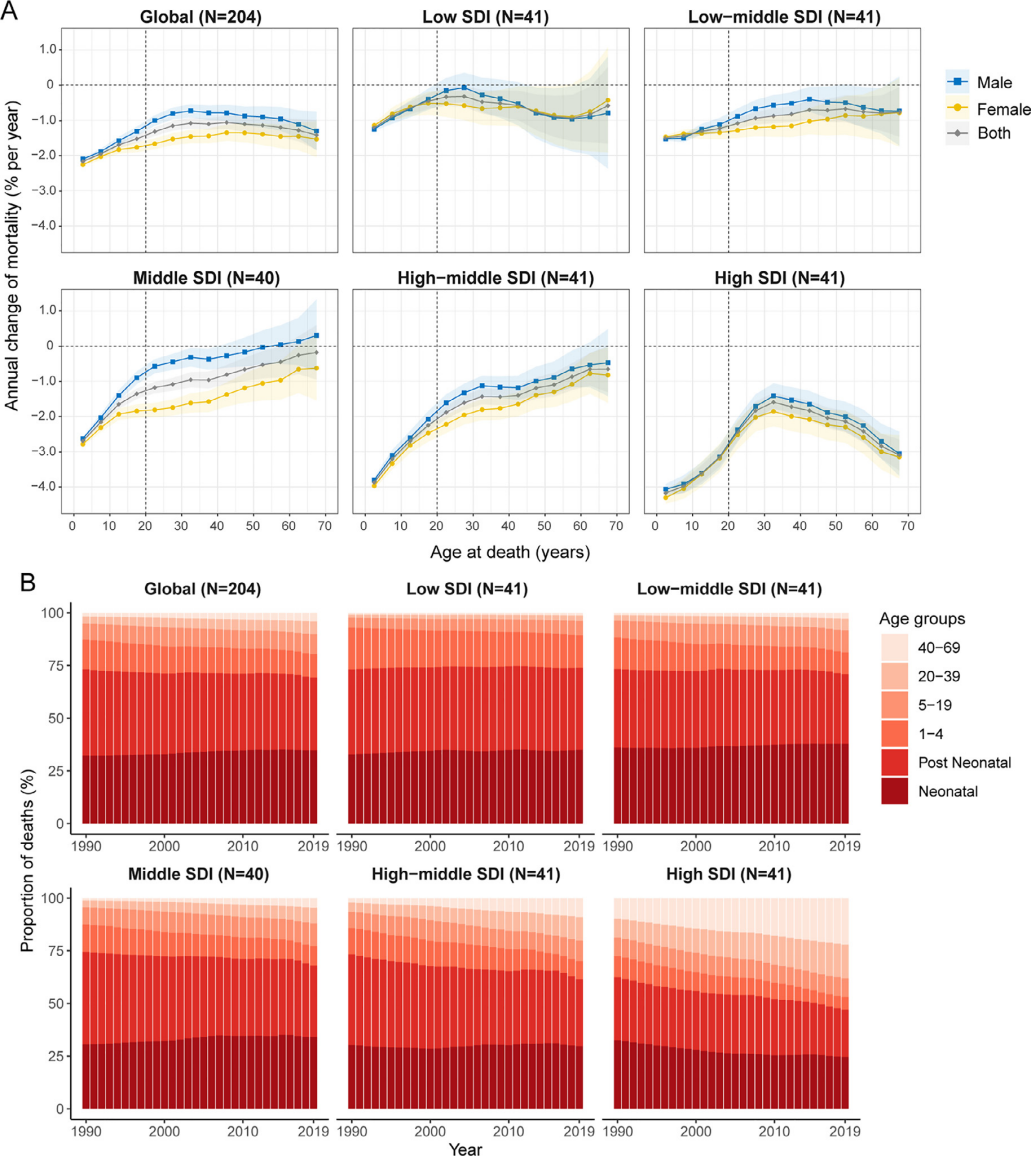
Local drifts of CHD mortality and age distribution of deaths from CHD by SDI quintiles, 1990–2019 (2A) Local drifts of CHD mortality (estimates from age-period-cohort models) for 14 age groups (0–4 to 65–69 years), 1990–2019. The dots and shaded areas indicate the annual percentage change of mortality (% per year) and the corresponding 95% CIs. (2B) Temporal change in the relative proportion of CHD deaths across age groups (neonatal, post neonatal, 1–4, 5–19, 20–39, 40–69 years), 1990–2019. Neonatal stage denotes < 30 days; Post neonatal denotes 30 days to 1 year. CHD=congenital heart disease; SDI=Socio-demographic Index.

Fig. 3A shows the annual percentage change in CHD mortality rate for each age group (ie, *local drift* of mortality estimated from the APC model and capturing trends in birth cohort effects), from 0 to 4 to 65–69 years. Globally, CHD mortality had decreasing trends across all age groups (*p* < 0.0001). The steepest mortality reduction occurred in the 0–4 years group (–2.17% per year, [95% CI –2.20 to –2.14], ie, equivalent to an overall ∼50% decrease in mortality rate over the past 30 years), and the declining trend attenuated with increasing age, becoming less favourable in adults (from –1.32% [–1.44 to –1.20] in adults 20–24 years to –1.42% [–1.84 to –1.00] in those 65–69 years). Males showed significantly slower mortality reductions than females in age groups where the 95% CI bounds did not overlap. In the paediatric population (0–19 years), mortality reduction was the greatest in high-SDI countries (from –4.17% [–4.28 to –4.05] in children 0–4 years to –3.15% [–3.41 to –2.90] in those 15–19 years) and was less in low-middle- and low-SDI countries. Local drifts for younger age groups under 25 are shown in appendix Fig. S2, which indicates wide disparities between higher- and lower-SDI regions. In the adult population (20–69 years), mortality reduction was generally less than in the paediatric population regardless of SDI quintile. In low-SDI countries, there was little improvement in mortality in adults older than 20 years. The local drift of mortality for each country is shown in appendix Figs. S3–S7.

Fig. 3B presents temporal changes in the age distribution of deaths, an indirect marker of CHD population survival. Globally, there was an emerging transition of deaths from the paediatric population (< 20 years) to the adult population (> 20 years), and this trend was more noticeable in middle-SDI, high-middle-SDI and high-SDI countries. In 2019, neonates (< 30 days) and post-neonatal infants (30 days to 1 year) accounted for the largest proportion of deaths, and this trend remained constant over the study period except in high-SDI countries. Over 90% of deaths were concentrated in children under 5 in low-SDI countries. Data for younger age groups under 25 were shown in appendix Fig. S2. The age distribution of deaths for each country is shown in appendix Figs. S8–S12.

### Age, period, and cohort effects on CHD mortality

**Figure 4:**
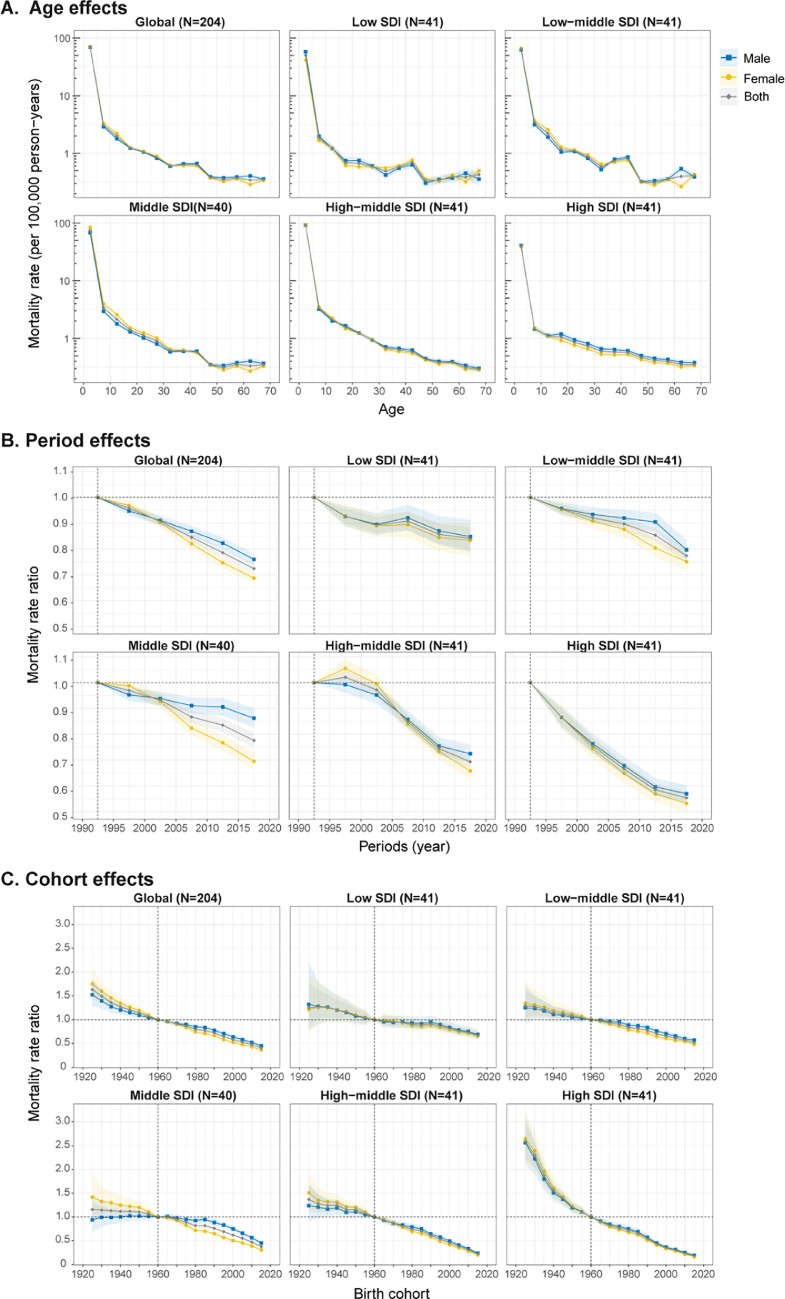
Age, period and cohort effects on CHD mortality by SDI quintiles (A) Age effects are shown by the fitted longitudinal age curves of mortality (per 100,000 person-years) adjusted for period deviations. (B) Period effects are shown by the relative risk of mortality (mortality rate ratio) and computed as the ratio of age-specific rates from 1990 to 1994 (the referent period) to 2015–2019. (C) Cohort effects are shown by the relative risk of mortality and computed as the ratio of age-specific rates from the 1925 cohort to the 2015 cohort, with the referent cohort set at 1960. The dots and shaded areas denote mortality rates or rate ratios and their corresponding 95% CIs. CHD=congenital heart disease; SDI=Socio-demographic Index.

Fig. 4 shows the APC model-derived estimates of age-period-cohort effects by SDI quintile (ie, *age effects*, expressed as longitudinal age curves to represent age-associated natural history of CHD mortality; *period effects*, expressed as relative risk of mortality by periods and used to track progress in different time periods; *cohort effects*, expressed as relative risk of mortality by cohorts and used to track changes in mortality for different birth cohorts). Generally, similar patterns in age effects were found across different SDI quintiles, with the highest risk in children aged 0–4 years, and with risk decreasing with age. Compared to other countries, high-SDI countries showed an overall lower mortality across all age groups, and the risk was lowest in those aged 5–19 years, suggesting better survival in this age group. No sex difference was found in age effects.

Period effects generally showed a declining risk of mortality across different SDI quintiles over the study period. However, for low-SDI countries, period effects remained nearly constant over the past two decades, indicating little mortality improvement. High-SDI countries had the most notable reduction of period risks across 1990–2019, whereas low-middle-SDI, middle-SDI, and high-middle-SDI countries had more favourable reduction of period risks only in the past ten years. In middle-SDI countries, period risk reduction was less for males than females.

Globally, there was an overall declining risk in successively younger birth cohorts. Similar to period effects, declining cohort effects were more noticeable in higher-SDI countries. High-SDI countries had progressive mortality improvements in those born after the 1920 s, whereas the risk in low-SDI countries had not decreased until after the 2000 cohort. Compared with individuals born in the referent 1960 cohort, the relative cohort risk for individuals born in the 2015 cohort ranged from 0.67 (95% CI 0.61–0.74) in low-SDI countries to 0.18 (0.17–0.20) in high-SDI countries. The age, period, and cohort effects on CHD mortality in each country are shown in appendix Figs.S13–S27.

### Age-period-cohort effects in exemplary countries

**Figure 5:**
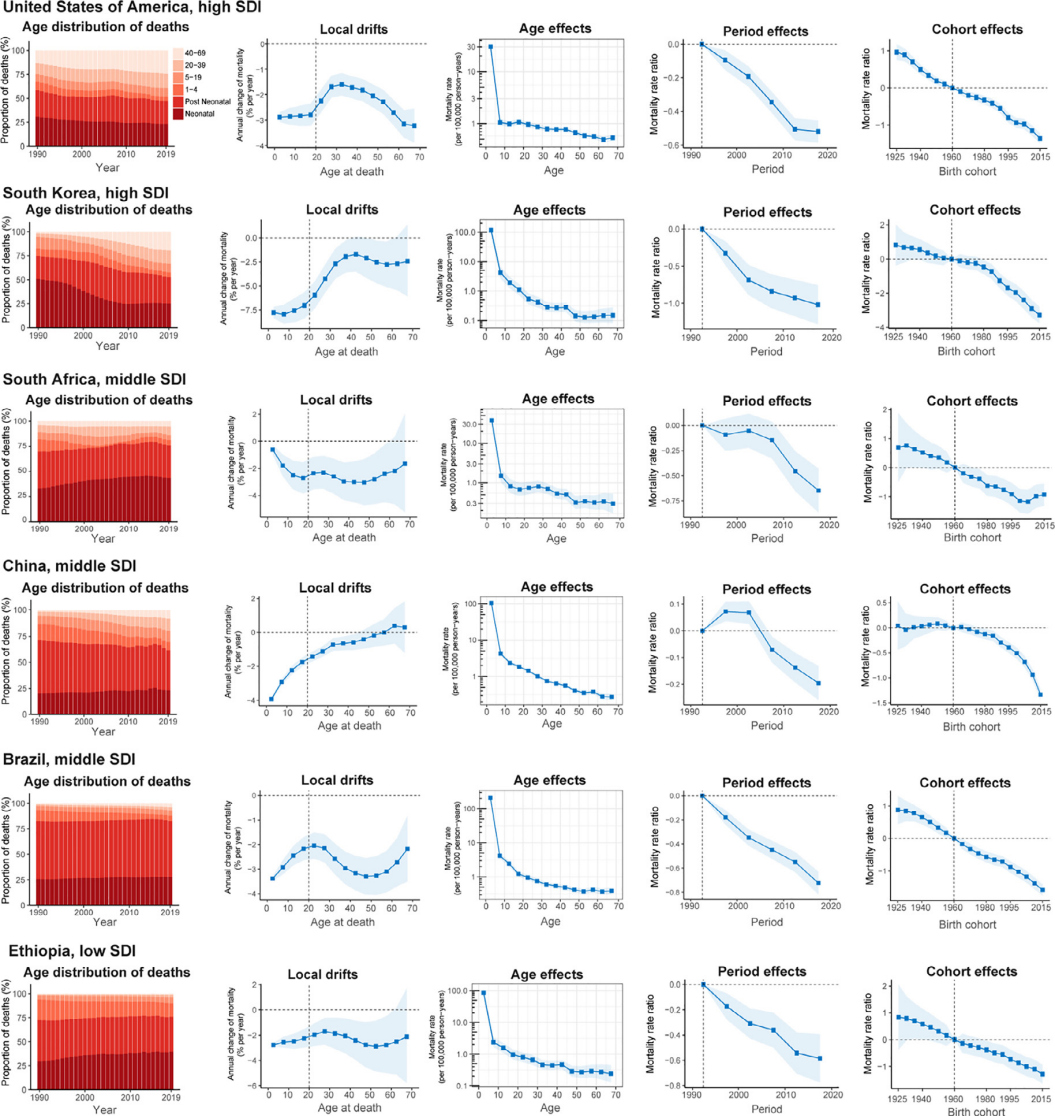

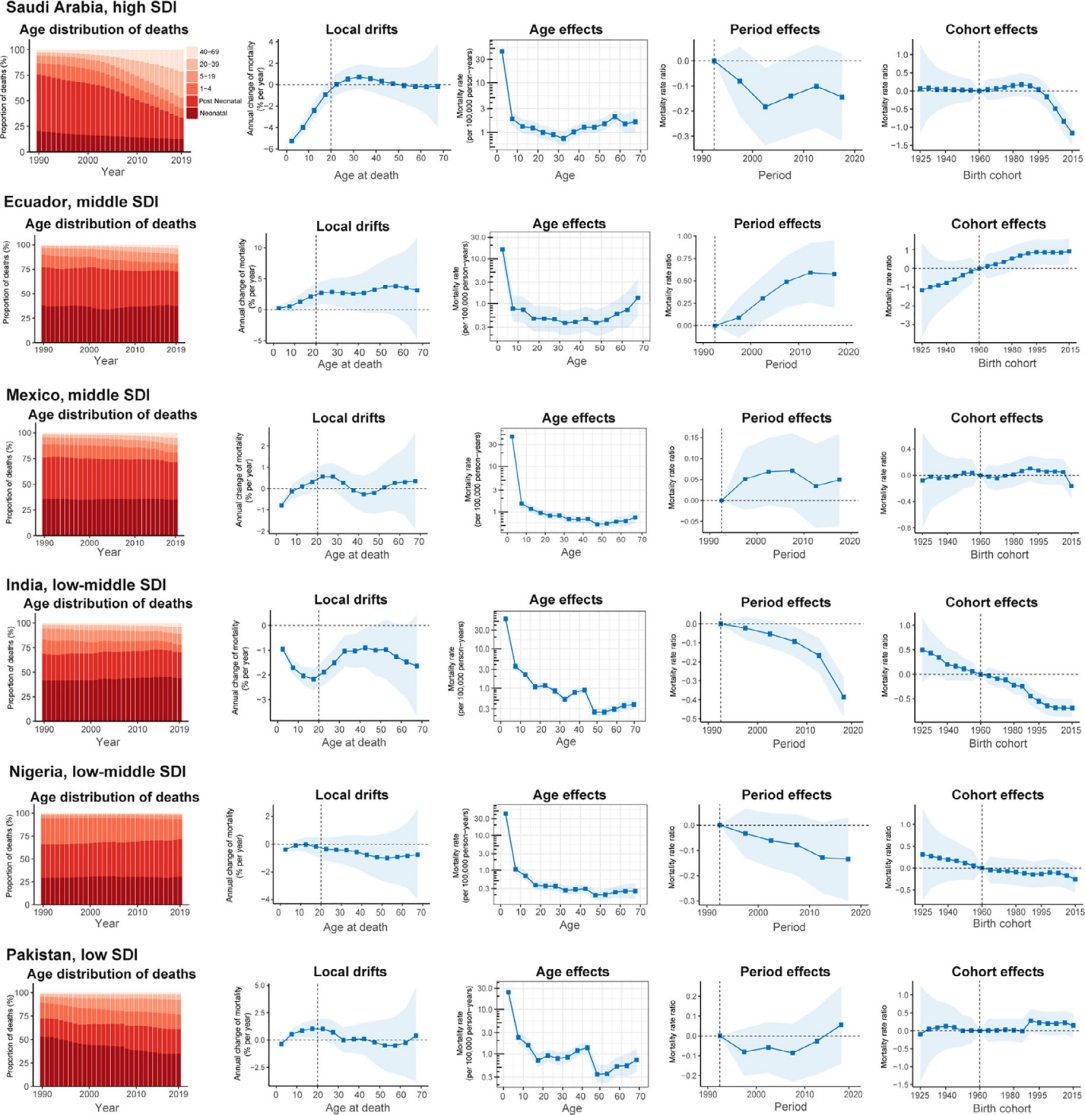
Favourable (A) and unfavourable (B) age-period-cohort effects on exemplar countries across SDI quintiles Age distribution of deaths shows the relative proportion of deaths from each age group during 1990–2019. Local drifts indicate the annual percentage change of mortality (% per year) across five-year age groups (from 0 to 4 to 65–69 years). Age effects are represented by the fitted longitudinal age curves of mortality (per 100,000 person-years) adjusted for period deviations. Period effects are represented by the relative risk of mortality (mortality rate ratio) and computed as the ratio of age-specific rates in each period compared to the referent 1990–1994 period. Cohort effects are represented by the relative risk of mortality (mortality rate ratio) and computed as the ratio of age-specific rates in each cohort compared to referent 1960 cohort. The shaded areas indicate the corresponding 95% CIs of each point estimate. SDI=Socio-demographic Index.

We presented several exemplary countries across SDI quintiles to better characterise the major trends in CHD mortality by age-period-cohort effects around the world. Fig. 5A shows countries with favourable age-period-cohort effects. The USA is typical of trends in high-SDI countries, where mortality reduction was found across all age groups and adults >40 years, accounting for a growing proportion of CHD deaths and favourable declining period and cohort risks. South Korea stood out for its notable net drift and demonstrated an emerging transition in the age distribution of deaths, but with mortality reduction attenuated in adults > 40 years. China also has had a transition in the age distribution of deaths, with significantly decreased risks in those born after 2000 and greater mortality in adults > 50 years. Brazil was the only middle-SDI country with significant mortality reduction for all age groups, with notable declining risk over the periods and in successive birth cohorts. South Africa showed relatively modest local drifts in mortality across age groups. Risks were lower from 2010 to 2015, but a declining cohort risk in those born after 2005. Ethiopia was a low-SDI country with a gradual reduction in the relative risk of mortality across periods and birth cohorts, in spite of a majority of deaths remaining concentrated in children under 5.

Fig. 5B presents countries with relatively unfavourable age-period-cohort effects on mortality. Saudi Arabia showed a transition in the age distribution of deaths like other high-SDI countries, but mortality increased in those 20–40 years and was unimproved in those over 40 years. Ecuador had the worst trends in CHD mortality amongst middle-SDI countries, with local drifts > 0% per year in 0–69 years of age and both period and cohort risks worsening in recent years. Mexico showed increasing mortality trends in those aged 10–34 years, with increased period effects over the entire study period. In India, mortality reduction in children under 5 was only –0.95% [95% CI –1.08 to –0.82] per year (ie, 26% reduction over the past 30 years), with mortality gains plateauing in cohorts born after 2005. In Nigeria and Pakistan, there was little improvement in either period or cohort trends in the past 30 years, and those aged 10–34 years in Pakistan had increasing mortality.

## Discussion

Mortality in CHD is an important indicator of progress in meeting SDG targets for ending avertable deaths in children under 5 years and reducing by one-third premature deaths from NCDs.^4^^,^^5^ Consistent with the prior GBD 2017 publication, our study showed that despite notable achievements over the past three decades, health disparities in CHD mortality appear to be widening around the world. Our analysis of GBD mortality estimates suggests that many higher-SDI countries have had declining CHD mortality, but economically disadvantaged countries in Africa, Asia, and Latin America have had little or no progress. Given that the analyses in some LMICs are based solely on mathematical models, further primary studies are needed to confirm our finding of little or no improvement in CHD mortality. The CHD mortality gains are not commensurate with what would be expected from country-level socioeconomic status (eg, Saudi Arabia, Mexico, Ecuador), assuming variations in the effectiveness of health care for this group correlate well with SDI. We also found that CHD deaths are increasingly occurring beyond early childhood and into adolescence and adulthood, suggesting the importance of health-care systems for CHD extending across the life span.

To our knowledge, this is the first use of APC models to analyse time trends in CHD mortality on a global scale and allowing comparisons between different countries. Compared with the prior GBD 2017 publication, the major contribution of our study to the field is that we provide a more granular understanding of disease trends, making the best use of data to generate public health insights. In particular, the examination of period and cohort effects enables us to differentiate the source of mortality trends by time periods and birth cohorts for each country, providing information for the effectiveness of CHD-related health-care services. Another important area of progress^2^^,^^18^ is the estimation of local drift values enabling us to capture time trends in mortality for each age group, adjusting for period effects.^16^ As for the mortality estimates, in GBD 2019, the inclusion of a new variable (birth prevalence of CHD) and the updated measurement of covariates (such as smoking and diabetes) into the CHD-specific models^1^ allowed for improved estimation of these fatal outcomes compared to GBD 2017.

Unlike many other cardiovascular diseases (such as ischaemic heart disease), the age-standardised prevalence, death, and disability-adjusted life-year estimates for CHD remain significantly lower than all-ages estimates in lower-income countries because of younger populations, higher birth rates, and restricted access to essential interventions for survival.^1^ Therefore, reliance on age-standardised rates to track trends in CHD mortality is likely to be misleading for lower-SDI regions. Moreover, assessing the change using an overall rate omits important information on differences across ages, time periods, and birth cohorts. In this study, we illustrated CHD mortality with both all-age rates and age-standardised rates, and we used APC model-derived estimates to facilitate an in-depth analysis of trends in CHD mortality.

Between 1990 and 2019, the global population increased by 45%, but the total number of CHD deaths decreased by 43%, with the largest reductions in higher-SDI countries in North America and Western Europe. In contrast, the population and the total number of CHD deaths in low-SDI countries increased by 114% and 24%, respectively. This is exemplified in our findings for Pakistan and Nigeria, where relative risks for CHD mortality have increased in birth cohorts born after 1990. Limited access to congenital cardiac care seems likely to be a major reason for a failure to make progress: the average volume of cardiac surgery in low-income countries is only 0.5 per million population, approximately one-thousandth of that in upper-middle-income countries.^19^ Pakistan is the second largest country (224 million) in south Asia but has only four facilities with a few specialists in CHD.^20^ Nigeria, the most populous country in Africa (215 million), has only 13 centres performing congenital heart surgeries, with an annual volume of only 21 cases per centre.^21^ The association between malnourishment and surgical mortality for CHD may also contribute to the limited progress in low-income settings.^22^

Current global efforts remain inadequate for meeting the growing needs for congenital cardiac care in lower-income countries. Although humanitarian congenital surgical missions that provide care and transfer skills have long-lasting societal benefits and are of reported high cost-effectiveness,^23^ many failed to expand or even declined during the period of study due to limited funding, equipment, and medical volunteers.^24^

India is the world's second most populous country (1.39 billion). Although the all-age mortality rate for CHD decreased by 55.4% during 1990–2019, it still accounted for the largest absolute number of CHD deaths globally. The mortality gain appeared to be driven by a favourable period effect in recent years, but the declining trend of mortality has remained constant for those born after 2005. India is the first country to establish a cardiac surgical programme in south Asia with many experienced professionals.^25^ However, the geographical distribution of cardiac facilities is uneven, and many centres are paradoxically located in states with a lower burden of CHD.^26^ Wide disparities in care between public and private sectors, together with financial constraints, are likely to be a further factor.^26^ A limited expansion of Indian government-funded cardiac services suggests that CHD will continue to be an important cause of NCD-related deaths.

In contrast to India, the all-age mortality of CHD in Brazil and China decreased by nearly 80%, with improvements over the study period and in successively younger birth cohorts. Mortality reduction in Brazil and China contributed significantly to the global decline. These gains are most likely to have come about from the establishment of self-sustaining programmes capable of performing congenital heart surgery with very low mortality.^27^^,^^28^ In China, nationwide efforts to screen for CHD in newborns^29^^,^^30^ have improved the detection of CHDs at an early stage and in turn contributed to declining CHD mortality. A unified government-funded system to reimburse treatment costs has not only reduced family financial burden but is also likely to have improved survival after surgery.^28^ Despite these gains, there is room for improvement in Brazil and China as their all-age mortality remains 2.5 to 4.5 times higher than that of high-SDI countries. In Brazil, nearly 60% of newly diagnosed patients are not financially covered for cardiac surgery,^31^ and specialised centres for paediatric cardiac surgery remain poorly structured and inadequately funded.^32^ In China, CHD mortality in rural children under 5 was about two-fold higher than in urban children,^33^ with underprivileged children having suboptimal outcomes after surgery.^34^ Maintaining the pace of CHD mortality reduction will require further strengthening of treatment capacity and follow-up management.^34^

Saudi Arabia, Mexico, and Ecuador represent countries with increasing trends in CHD mortality not commensurate with their socioeconomic development. Saudi Arabia is a wealthy country in the Middle East with a 2.5-fold higher all-age mortality than other high-SDI countries, unfavourable period effects, and diminished cohort risks for those born after 2000. The number of registered paediatric cardiac surgeons in Saudi Arabia was 1–2 per million population, which was comparable to other high-income countries, like South Korea.^35^ The high prevalence of extra-cardiac anomalies (such as Trisomy 21) in Saudi patients with CHD may contribute to the poor outcomes, but further investigation is clearly warranted.^36^ Ecuador is a middle-SDI country with increasing mortality risks in recent periods and birth cohorts, and 86% of patients with newly diagnosed CHD each year do not receive timely cardiac surgery.^31^ Mexico is a middle-SDI country but with all-age mortality 1.3-fold higher than the global level and worsening trends over time and cohort.^37^ The Mexican health-care system is fragmented, and CHD patients are treated in different institutions without integrated and uniform standards of care.^38^ In-hospital mortality for congenital heart surgery is substantially higher than in peer countries.^39^ For these countries, there is a pressing need to improve quality of care and to strengthen state and subregional CHD health services.^40^

Globally, adult patients with CHD outnumber children,^6^ and their number continues to increase by 5% each year.^41^ In many higher-SDI countries, adults account for a rising proportion of CHD deaths, particularly for those over 40 years of age. This corroborates previous work showing that in high-income countries, the median age at death in the CHD population had increased from 37 to 57 years.^42^ In the USA, mortality reduction was found in all age groups and was more pronounced in those > 60 years.^10^ However, in many other countries, mortality reduction has been less favourable for adolescents and adults. Amongst higher-SDI countries like South Korea and Saudi Arabia, mortality reduction attenuates with increasing age, with trends toward greater CHD mortality in older adults over time. China has similar unfavourable trends in those > 50 years. In line with the shift of age distribution of deaths towards the adult population in many countries, managing adult CHD patients will present challenges to health systems that have been orientated to paediatric populations.

Adult CHD is associated with heightened risks of cardiovascular diseases^43^ and cancer,^44^ with differing excess risks of death compared to the general population.^45^ This excess risk could originate from the disease per se and from surgical complications. For example, children who received cardiac surgery had a 12-fold higher risk of developing hypertension in later life, and this risk was partly associated with in-hospital events during the index surgery.^46^ Without regular and proper follow-up care, premature deaths in adult CHD patients are frequent. However, specialist resources for adult CHDs are very limited around the world^47^ and many programmes are not well organised.^48^ Moreover, the current management pathway of adult CHD patients is fraught with multiple problems, including loss to follow-up, a low proportion of adult patients receiving care at expert centres,^49^ and unsatisfactory delivery of guideline-recommended care.^50^ Given the growing disease burden and suboptimal progress made in adolescent and adult populations, there is a need to increase investment in specialist resources for adult CHD.

There are several cautions when translating our results into public health perspectives. First, estimates from LMICs without primary data (eg, Papua New Guinea, Yemen, and Sudan) should be treated with caution as they derive from models and await verification from other independent primary studies. Second, our analysis utilised country-level mortality estimates data to feed into the APC model; hence, the conclusions should not be directly extrapolated to subnational regions as geographical health disparities persist in almost all countries and progress can be achieved in certain regions but not others. Third, a comparison of our results with the trends in infant and neonatal heart operations as well as the surgical mortality for each country could complement the assessment of congenital heart service availability and quality; however, due to a lack of publicly available data, we did not perform such an analysis. Future studies using an international database like International Quality Improvement Collaborative for Congenital Heart Disease (IQIC), or a systematic analysis of congenital heart programmes in each country, may fill this knowledge gap.

Our study presents an example of performing an in-depth analysis of disease trends using GBD data. For other causes of NCDs, the application of APC models to analyse disease trends by age, period, and cohorts may also offer greater clarity about the effectiveness of health system responses beyond conventional epidemiological metrics and thus facilitate the tracking of country-specific progress towards SDG targets.

The study has several limitations. Firstly, our analyses have limitations that derive from GBD models, as a result of limited primary data in LMICs and estimates informed by data from higher-resource settings.^2^ Many countries estimated to have the highest mortality do not have primary data on CHD. GBD mortality estimates for these countries have wide uncertainty bounds as a result of being largely driven by covariates.^11^ This might affect estimates of age/period/cohort trends and may overstate the improvement in some low-SDI countries. There is a pressing need for better primary data on cardiovascular disease mortality in many LMICs, which might include longer-term cohort studies and registries of those born with CHD. Second, the GBD study uses mutually exclusive causes and a single underlying cause of death for CHD.^1^ For older adults, CHD becomes less likely to be coded as the underlying cause of death, and deaths arising from complications such as pulmonary arterial hypertension may not be included. Therefore, the actual death rates from CHD in the GBD study may have been underestimated. To address the changing age distribution of CHD, integrative disease surveillance systems should ideally be established to capture CHD-related morbidity and mortality for all age groups. Thirdly, this study used five-year age group mortality estimates data freely accessed from GBD 2019, also the most commonly used age-interval data format in APC models.^16^ This creates partially overlapping birth cohorts, and so we could not determine the effects for the youngest age group (< 1 year). Fourth, we did not analyse mortality trends for different CHD subtypes due to data unavailability. The incorporation of sub-classification data could better specify the types of CHD that warrant special attention for each country/region. Fifth, this study analysed mortality data at the national level and does not capture subnational differences. A more granular analysis using subnational data could identify areas with different trends. Finally, our study did not include direct country-level data on health system quality or access to care. In future iterations of GBD, the inclusion of indicators for surgical availability and surgical quality may improve the accuracy of CHD mortality estimates, and the inclusion of more data on critical CHD screening and surgical/catheter-based intervention availability may help to differentiate the relative contribution of timely identification of disease compared to lack of treatment access on CHD mortality.

CHD is an important cause of the remaining global mortality from NCDs. Our age-period-cohort analysis of global CHD mortality found that mortality gains in the past 30 years are not always commensurate with a country's socioeconomic development. Unfavourable period and cohort effects reveal that current resources are largely inadequate to manage CHD populations in many countries that could afford better health care for this group. The CHD mortality is therefore a useful indicator of trends in the provision and accessibility of congenital cardiac care both in early childhood and across later life. Increasingly, countries will need to extend health care for those with CHD beyond paediatric settings to those who increasingly survive into adulthood.

## Contributors

ZS, ZZ and HZ conceived the study. ZS accessed and acquired the raw data, performed the primary analysis, prepared tables and figures, and drafted the first manuscript. YL, SL and HC contributed to the interpretation of the data. MSZ, LBW, GRM, CAS, and SIH critically reviewed results and provided valuable inputs on revision. NJK, GCP and SIH substantially edited and critically reviewed the manuscript. MN managed all of the mortality modelling data. CJLM managed the overall research enterprise. HZ and ZZ were responsible for general supervision and had final responsibility for the decision to submit for publication. All authors reviewed the article, read the final manuscript and approved the submission.

### Data sharing

All data used in this study can be freely accessed at the GBD 2019 portal (http://ghdx.healthdata.org/gbd-2019).

### Funding

Supported by the 10.13039/501100001809National Natural Science Foundation of China (81525002, 31971048, 82073573 to ZZ and HZ), Shanghai Outstanding Medical Academic Leader program (2019LJ22 to HZ), and Collaborative Innovation Program of Shanghai Municipal Health Commission (2020CXJQ01 to HZ), the Bill & Melinda Gates Foundation for the Global Burden of Disease Project (to NJK) and NHMRC fellowship administered through the University of Melbourne (to GCP).

## Declaration of interests

NJK reports consulting fees from Merck as a one-day consultation on a potential public visualization tool. All the other authors declare no competing interests.

## Appendix Supplementary materials

Image, application 1
