# Supplementary material for: Global, regional, and national time trends in mortality for congenital heart disease, 1990–2019: An age-period-cohort analysis for the Global Burden of Disease 2019 study
Source: eClinicalMedicine. 2022 Jan 11;43:101249. doi: 10.1016/j.eclinm.2021.101249 (PMC8760503; doi:10.1016/j.eclinm.2021.101249)
Supplement: Supplementary file 1 [file mmc1.docx]

**Supplementary appendix to**

**Global, Regional and National Time Trends in Mortality for Congenital Heart Disease, 1990-2019: An Age-Period-Cohort Analysis for the GBD 2019**

**Table S1. The lexis diagram of GBD data for the APC model**

|  | **Age groups** | | | | | | | | | | | | | | **Birth cohort (median)** |
| --- | --- | --- | --- | --- | --- | --- | --- | --- | --- | --- | --- | --- | --- | --- | --- |
| **Period**  **(median)** | **0-4** | **5-9** | **10-14** | **15-19** | **20-24** | **25-29** | **30-34** | **35-39** | **40-44** | **45-49** | **50-54** | **55-59** | **60-64** | **65-69** |  |
|  |  |  |  |  |  |  |  |  |  |  |  |  |  | X | 1921-1929 (1925) |
|  |  |  |  |  |  |  |  |  |  |  |  |  | X | X | 1926-1934 (1930) |
|  |  |  |  |  |  |  |  |  |  |  |  | X | X | X | 1931-1939 (1935) |
|  |  |  |  |  |  |  |  |  |  |  | X | X | X | X | 1936-1944 (1940) |
|  |  |  |  |  |  |  |  |  |  | X | X | X | X | X | 1941-1949 (1945) |
|  |  |  |  |  |  |  |  |  | X | X | X | X | X | X | 1946-1954 (1950) |
|  |  |  |  |  |  |  |  | X | X | X | X | X | X |  | 1951-1959 (1955) |
|  |  |  |  |  |  |  | X | X | X | X | X | X |  |  | 1956-1964 (1960) |
|  |  |  |  |  |  | X | X | X | X | X | X |  |  |  | 1961-1969 (1965) |
|  |  |  |  |  | X | X | X | X | X | X |  |  |  |  | 1966-1974 (1970) |
|  |  |  |  | X | X | X | X | X | X |  |  |  |  |  | 1971-1979 (1975) |
|  |  |  | X | X | X | X | X | X |  |  |  |  |  |  | 1976-1984 (1980) |
|  |  | X | X | X | X | X | X |  |  |  |  |  |  |  | 1981-1989 (1985) |
|  | **X** | X | X | X | X | X |  |  |  |  |  |  |  |  | 1986-1994 (1990) |
| 1990-1994 (1992) | X | X | X | X | X |  |  |  |  |  |  |  |  |  | 1991-1999 (1995) |
| 1995-1999 (1997) | X | X | X | X |  |  |  |  |  |  |  |  |  |  | 1996-2004 (2000) |
| 2000-2004 (2002) | X | X | X |  |  |  |  |  |  |  |  |  |  |  | 2001-2009 (2005) |
| 2005-2009 (2007) | X | X |  |  |  |  |  |  |  |  |  |  |  |  | 2006-2014 (2010) |
| 2010-2014 (2012) | X |  |  |  |  |  |  |  |  |  |  |  |  |  | 2011-2019 (2015) |
| 2015-2019 (2017) |  |  |  |  |  |  |  |  |  |  |  |  |  |  |  |

Note: X denotes mortality data of each age group from the corresponding period. For instance, the mortality rate of age 0-4 years in 1992 is filled in the square with a bold **X** (see table), and this square belongs to the cohort 1986-1994 (1990).

**Table S2. Time trends in congenital heart disease mortality for both sexes in 129 countries, 1990-2019**

| **SDI quintile** | **Country** | **Population** | | **Deaths** | | **All-age mortality** | | **Age-standardized mortality** | | **Net drift of mortality from APC model †,**  **% per year** |
| --- | --- | --- | --- | --- | --- | --- | --- | --- | --- | --- |
|  |  | **Number in 2019** | **Percent change 1990-2019, %** | **Number in 2019** | **Percent change 1990-2019, %** | **Rate in**  **2019** | **Percent change 1990-2019, %** | **Rate in**  **2019** | **Percent change 1990-2019, %** |  |
| **High SDI** | Australia | 24568113 (22510058, 26779186) | 45.7 | 149 (105, 191) | -47.9 (-59.2, -17.7) | 0.61 (0.43, 0.78) | -64.2 (-72.0, -43.5) | 0.80 (0.56, 1.07) | -61.3 (-70.1, -35.2) | -2.51 (-3.48, -1.52) |
|  | Canada | 36519840 (33331463, 39599796) | 34.0 | 228 (175, 267) | -59.1 (-65.2, -45.7) | 0.62 (0.48, 0.73) | -69.5 (-74.0, -59.5) | 0.95 (0.71, 1.13) | -63.6 (-69.5, -49.2) | -2.78 (-3.53, -2.02) |
|  | France | 66204315 (60093850, 72433669) | 14.6 | 396 (312, 511) | -66.3 (-71.5, -51.8) | 0.60 (0.47, 0.77) | -70.6 (-75.1, -58.0) | 0.92 (0.74, 1.27) | -67.8 (-73.5, -49.7) | -2.90 (-3.50, -2.29) |
|  | Germany | 84914056 (77688648, 92219489) | 6.2 | 355 (259, 432) | -74.5 (-79.0, -61.8) | 0.42 (0.31, 0.51) | -76.0 (-80.2, -64.0) | 0.75 (0.54, 0.96) | -73.5 (-78.6, -56.9) | -3.03 (-3.66, -2.41) |
|  | Japan | 127788411 (115774123, 139878471) | 1.5 | 551 (429, 696) | -74.1 (-77.2, -60.9) | 0.43 (0.34, 0.54) | -74.5 (-77.6, -61.5) | 0.86 (0.61, 1.12) | -70.4 (-74.7, -53.6) | -2.71 (-3.17, -2.24) |
|  | Kuwait | 4426561 (3926462, 4929515) | 151.6 | 104 (80, 137) | -39.7 (-57.9, -7.9) | 2.34 (1.81, 3.10) | -76.0 (-83.3, -63.4) | 3.34 (2.57, 4.43) | -62.6 (-74.0, -42.7) | -3.30 (-6.09, -0.43) |
|  | Netherlands | 17156788 (15675163, 18613311) | 15.0 | 85 (68, 121) | -71.1 (-76.7, -52.5) | 0.49 (0.40, 0.70) | -74.9 (-79.7, -58.7) | 0.79 (0.62, 1.20) | -71.0 (-77.6, -49.0) | -4.27 (-5.49, -3.03) |
|  | Republic of Korea | 53398252 (48441017, 58407132) | 20.4 | 186 (153, 229) | -89.5 (-92.2, -77.3) | 0.35 (0.29, 0.43) | -91.3 (-93.5, -81.1) | 0.69 (0.54, 0.86) | -86.7 (-90.5, -70.6) | -3.95 (-4.83, -3.07) |
|  | Russian Federation | 146717427 (128850161, 165171807) | -2.9 | 1599 (1048, 2237) | -63.5 (-71.6, -32.0) | 1.09 (0.71, 1.52) | -62.4 (-70.8, -30.0) | 1.63 (0.99, 2.32) | -61.1 (-71.4, -23.1) | -1.11 (-1.45, -0.77) |
|  | Saudi Arabia | 35731972 (31175706, 40192039) | 122.7 | 640 (474, 855) | -58.5 (-76.4, -18.9) | 1.79 (1.33, 2.39) | -81.4 (-89.4, -63.6) | 2.16 (1.34, 3.05) | -66.8 (-81.4, -35.0) | -0.42 (-1.05, 0.21) |
|  | Slovakia | 5437223 (4969934, 5923604) | 2.9 | 53 (39, 72) | -70.4 (-79.0, -56.9) | 0.98 (0.72, 1.33) | -71.2 (-79.6, -58.1) | 1.69 (1.23, 2.32) | -62.1 (-73.4, -44.0) | -1.67 (-3.73, 0.45) |
|  | Sweden | 10222546 (9312312, 11127453) | 19 | 52 (44, 73) | -71.7 (-76.3, -55.8) | 0.51 (0.43, 0.72) | -76.2 (-80.1, -62.8) | 0.70 (0.56, 1.10) | -74.8 (-79.9, -57.2) | -3.17 (-4.59, -1.72) |
|  | United Arab Emirates | 9241704 (7764642, 10592699) | 393.7 | 69 (43, 103) | -54.6 (-75.2, -18.3) | 0.75 (0.46, 1.12) | -90.8 (-95.0, -83.4) | 1.71 (0.94, 2.75) | -75.2 (-87.9, -50.0) | -1.53 (-4.66, 1.70) |
|  | United Kingdom | 67220447 (60468668, 73925429) | 17.0 | 527 (466, 705) | -61.4 (-66.4, -48.0) | 0.78 (0.69, 1.05) | -67.0 (-71.3, -55.5) | 1.09 (0.92, 1.56) | -65.0 (-69.8, -49.3) | -2.99 (-3.48, -2.50) |
|  | United States of America | 327978730 (285959303, 369324168) | 29.3 | 2842 (2538, 3508) | -51.5 (-55.9, -40.9) | 0.87 (0.77, 1.07) | -62.5 (-65.9, -54.3) | 1.20 (1.07, 1.49) | -55.9 (-60.4, -43.4) | -2.25 (-2.47, -2.03) |
| **High-middle SDI** | Argentina | 45115284 (39507183, 51073381) | 36.2 | 879 (548, 1128) | -43.1 (-62.6, -17.3) | 1.95 (1.22, 2.50) | -58.2 (-72.6, -39.3) | 2.50 (1.54, 3.23) | -46.0 (-65.0, -21.4) | -1.11 (-1.92, -0.29) |
|  | Belarus | 9500785 (8345414, 10677716) | -9.3 | 147 (100, 221) | -67.4 (-77.7, -46.7) | 1.54 (1.05, 2.33) | -64.1 (-75.5, -41.3) | 2.24 (1.47, 3.48) | -62.2 (-75.4, -32.3) | -1.60 (-2.55, -0.64) |
|  | Bulgaria | 6934625 (6359976, 7553856) | -20.1 | 96 (68, 125) | -68.0 (-76.9, -37.9) | 1.39 (0.99, 1.81) | -60.0 (-71.1, -22.2) | 2.82 (2.01, 3.72) | -50.7 (-64.6, -1.5) | -0.64 (-2.33, 1.07) |
|  | Chile | 18198359 (16753483, 19617223) | 37.0 | 223 (146, 302) | -56.1 (-73.1, -34.6) | 1.23 (0.80, 1.66) | -68.0 (-80.4, -52.3) | 1.82 (1.18, 2.53) | -48.8 (-69.1, -21.5) | -1.70 (-2.90, -0.50) |
|  | Greece | 10337172 (9070617, 11489319) | -0.5 | 63 (47, 78) | -75.4 (-81.7, -44.5) | 0.61 (0.45, 0.75) | -75.3 (-81.6, -44.2) | 1.26 (0.85, 1.62) | -72.2 (-79.5, -37.7) | -1.81 (-3.76, 0.18) |
|  | Hungary | 9674413 (8515522, 10788963) | -6.9 | 84 (57, 119) | -75.0 (-84.4, -61.6) | 0.87 (0.59, 1.23) | -73.2 (-83.2, -58.8) | 1.47 (1.02, 2.34) | -70.8 (-80.8, -49.7) | -2.05 (-3.18, -0.90) |
|  | Israel | 9309583 (8164697, 10550907) | 87.6 | 72 (55, 93) | -54.3 (-66.9, -0.5) | 0.77 (0.60, 1.00) | -75.7 (-82.3, -47.0) | 0.77 (0.59, 1.00) | -75.4 (-82.1, -46.4) | -2.75 (-5.15, -0.29) |
|  | Italy | 60313170 (55356080, 64983929) | 6.2 | 301 (196, 375) | -68.0 (-78.0, -48.9) | 0.50 (0.33, 0.62) | -69.9 (-79.3, -51.9) | 0.95 (0.66, 1.26) | -68.1 (-76.1, -40.9) | -2.22 (-2.87, -1.58) |
|  | Jordan | 11636717 (10588262, 12678912) | 208.4 | 588 (433, 797) | -0.4 (-38.5, 73.0) | 5.05 (3.72, 6.85) | -67.7 (-80.1, -43.9) | 4.90 (3.60, 6.66) | -47.8 (-67.7, -9.6) | -2.63 (-4.87, -0.34) |
|  | Kazakhstan | 18392068 (16794137, 19921602) | 12.4 | 523 (229, 859) | -22.1 (-58.2, 30.1) | 2.84 (1.24, 4.67) | -30.7 (-62.8, 15.7) | 2.95 (1.28, 4.90) | -23.6 (-59.1, 28.1) | 0.87 (-0.07, 1.82) |
|  | Lebanon | 5177069 (4455291, 5928542) | 58.1 | 167 (85, 288) | -56.3 (-80.1, -17.1) | 3.23 (1.64, 5.56) | -72.3 (-87.4, -47.5) | 3.29 (1.66, 5.65) | -56.6 (-80.1, -18.4) | -1.30 (-3.77, 1.23) |
|  | Libya | 6735543 (5705382, 7670829) | 59.0 | 134 (91, 191) | -75.4 (-87.6, -44.4) | 1.99 (1.35, 2.83) | -84.5 (-92.2, -65.0) | 2.93 (1.93, 4.24) | -62.9 (-81.6, -16.5) | -1.02 (-2.91, 0.91) |
|  | Malaysia | 31301402 (27339262, 35190542) | 77.3 | 441 (328, 604) | -35.0 (-61.9, 17.6) | 1.41 (1.05, 1.93) | -63.3 (-78.5, -33.6) | 1.59 (1.17, 2.19) | -46.7 (-68.6, -4.1) | -1.65 (-2.60, -0.68) |
|  | Oman | 4583999 (4209600, 4952285) | 135.9 | 93 (68, 124) | -46.2 (-73.0, 31.6) | 2.03 (1.49, 2.71) | -77.2 (-88.6, -44.2) | 2.42 (1.79, 3.23) | -57.1 (-78.2, 1.6) | -2.16 (-5.44, 1.24) |
|  | Poland | 38434445 (35378981, 41364935) | 0.7 | 290 (191, 403) | -81.4 (-87.3, -61.1) | 0.75 (0.50, 1.05) | -81.6 (-87.4, -61.4) | 1.36 (0.84, 1.99) | -75.8 (-84.5, -47.0) | -2.87 (-3.65, -2.09) |
|  | Romania | 19237066 (17030070, 21542480) | -17.8 | 195 (136, 245) | -82.0 (-87.3, -51.0) | 1.01 (0.71, 1.28) | -78.1 (-84.5, -40.4) | 2.03 (1.41, 2.60) | -69.6 (-78.9, -18.3) | -1.89 (-3.15, -0.62) |
|  | Serbia | 8746785 (7829777, 9730592) | -6.9 | 64 (46, 87) | -88.9 (-94.5, -77.5) | 0.73 (0.53, 1.00) | -88.1 (-94.1, -75.8) | 1.29 (0.90, 1.83) | -85.1 (-93.0, -68.9) | -2.85 (-4.50, -1.16) |
|  | Spain | 46021218 (42087988, 49981539) | 18.7 | 204 (151, 269) | -73.5 (-78.8, -52.3) | 0.44 (0.33, 0.58) | -77.7 (-82.2, -59.8) | 0.82 (0.64, 1.13) | -75.7 (-80.8, -51.6) | -3.81 (-4.58, -3.03) |
|  | Turkey | 81359693 (71366134, 91236684) | 36.1 | 2215 (1662, 2877) | -76.5 (-88.0, -39.0) | 2.72 (2.04, 3.54) | -82.8 (-91.2, -55.2) | 4.45 (3.30, 5.84) | -63.4 (-81.5, -4.6) | -3.13 (-3.77, -2.49) |
|  | Ukraine | 44042431 (35745535, 52268016) | -16.4 | 689 (526, 1066) | -67.6 (-78.6, -36.6) | 1.56 (1.19, 2.42) | -61.2 (-74.4, -24.2) | 2.49 (1.87, 3.87) | -58.3 (-72.8, -14.8) | -1.23 (-1.66, -0.81) |
| **Middle SDI** | Albania | 2720353 (2418308, 3021834) | -17.8 | 75 (45, 104) | -58.5 (-79.5, -40.7) | 2.74 (1.66, 3.82) | -49.6 (-75.1, -27.9) | 4.29 (2.56, 6.01) | -2.8 (-52.6, 39.4) | 1.13 (-2.64, 5.05) |
|  | Algeria | 41847290 (36020518, 47461912) | 65.5 | 2126 (1556, 2880) | -53.5 (-74.3, 0.2) | 5.08 (3.72, 6.88) | -71.9 (-84.5, -39.5) | 5.02 (3.68, 6.81) | -60.0 (-77.9, -14.3) | -1.95 (-2.81, -1.08) |
|  | Armenia | 3019674 (2651852, 3385871) | -11.6 | 61 (37, 83) | -66.1 (-84.7, -34.1) | 2.03 (1.23, 2.74) | -61.7 (-82.6, -25.5) | 2.94 (1.78, 4.11) | -40.4 (-73.1, 18.7) | 0.28 (-2.00, 2.61) |
|  | Azerbaijan | 10278674 (8953532, 11640072) | 40.2 | 331 (192, 556) | -54.1 (-73.0, -25.7) | 3.22 (1.87, 5.41) | -67.3 (-80.7, -47.0) | 4.57 (2.61, 7.70) | -42.9 (-66.5, -7.5) | -1.44 (-3.65, 0.82) |
|  | Brazil | 216664814 (189879232, 242502434) | 45.6 | 6932 (5350, 8601) | -67.0 (-78.9, -25.5) | 3.20 (2.47, 3.97) | -77.3 (-85.5, -48.8) | 4.44 (3.41, 5.55) | -65.1 (-77.8, -21.1) | -2.72 (-3.05, -2.39) |
|  | China | 1422350422 (1239302360, 1597063538) | 20.2 | 25312 (21314, 30078) | -74.6 (-83.5, -63.8) | 1.78 (1.50, 2.11) | -78.8 (-86.3, -69.9) | 3.01 (2.49, 3.63) | -64.6 (-77.4, -48.8) | -0.99 (-1.22, -0.76) |
|  | Colombia | 47776679 (44174337, 51539501) | 46.8 | 1383 (789, 2039) | -36.3 (-66.4, -2.6) | 2.90 (1.65, 4.27) | -56.6 (-77.1, -33.6) | 3.51 (1.99, 5.19) | -30.1 (-63.2, 7.1) | -0.70 (-1.50, 0.10) |
|  | Costa Rica | 4716744 (4164759, 5271508) | 55.2 | 67 (37, 95) | -58.0 (-70.7, -35.5) | 1.41 (0.77, 2.01) | -72.9 (-81.1, -58.4) | 1.94 (1.03, 2.79) | -52.1 (-66.6, -27.0) | 0.00 (-3.66, 3.79) |
|  | Cuba | 11358510 (10094746, 12738845) | 4.9 | 109 (81, 164) | -74.8 (-81.7, -54.1) | 0.96 (0.72, 1.44) | -76.0 (-82.5, -56.2) | 1.57 (1.13, 2.57) | -66.0 (-75.8, -34.0) | -2.95 (-4.02, -1.88) |
|  | Ecuador | 17588392 (15403894, 19749863) | 75.4 | 700 (406, 985) | 25.4 (-36.5, 91.9) | 3.98 (2.31, 5.60) | -28.5 (-63.8, 9.4) | 4.08 (2.36, 5.76) | 2.1 (-48.7, 56.4) | 2.65 (1.18, 4.14) |
|  | Egypt | 99069551 (90571861, 107515771) | 77.9 | 2513 (1120, 4791) | -46.9 (-80.4, 32.1) | 2.54 (1.13, 4.84) | -70.2 (-89.0, -25.8) | 2.43 (1.08, 4.62) | -55.7 (-83.4, 9.2) | -0.82 (-1.51, -0.12) |
|  | Fiji | 911248 (838786, 984189) | 20.0 | 51 (36, 71) | 20.0 (-21.1, 83.6) | 5.58 (3.92, 7.74) | 0.0 (-34.3, 53.0) | 5.78 (4.04, 8.06) | 23.0 (-19.3, 88.8) | -0.25 (-3.84, 3.46) |
|  | Georgia | 3664752 (3306198, 4043348) | -33.5 | 53 (20, 76) | -60.0 (-84.1, -38.0) | 1.44 (0.55, 2.08) | -39.8 (-76.1, -6.8) | 2.14 (0.80, 3.14) | -31.5 (-73.3, 10.6) | 1.90 (-0.81, 4.68) |
|  | Indonesia | 259465835 (226843397, 291997879) | 40.0 | 7813 (5933, 10220) | -44.3 (-67.9, 12.5) | 3.01 (2.29, 3.94) | -60.2 (-77.0, -19.6) | 3.89 (2.92, 5.12) | -37.4 (-63.9, 26.3) | -0.55 (-0.88, -0.23) |
|  | Iran (Islamic Republic of) | 84297882 (77330567, 91935910) | 44.0 | 2175 (1495, 2971) | -77.2 (-90.8, -47.5) | 2.58 (1.77, 3.52) | -84.1 (-93.6, -63.6) | 3.18 (2.16, 4.38) | -71.3 (-88.5, -34.0) | -0.90 (-1.60, -0.20) |
|  | Iraq | 42119490 (31429294, 52981880) | 139.4 | 1643 (1085, 2495) | -48.1 (-76.7, 23.6) | 3.90 (2.58, 5.92) | -78.3 (-90.3, -48.4) | 3.54 (2.34, 5.37) | -64.6 (-83.9, -17.8) | -1.71 (-2.57, -0.84) |
|  | Jamaica | 2810754 (2482603, 3132031) | 18.9 | 55 (35, 78) | -46.0 (-65.7, -7.2) | 1.95 (1.26, 2.77) | -54.6 (-71.2, -21.9) | 2.77 (1.74, 3.99) | -26.3 (-53.6, 27.5) | 0.89 (-2.20, 4.08) |
|  | Mexico | 124940175 (108607132, 140630348) | 46.1 | 4583 (2728, 5989) | -26.7 (-72.1, 13.5) | 3.67 (2.18, 4.79) | -49.9 (-80.9, -22.3) | 4.32 (2.58, 5.68) | -20.2 (-69.2, 23.7) | 0.12 (-0.29, 0.52) |
|  | Panama | 4160457 (3659255, 4679229) | 74.2 | 166 (90, 242) | -3.9 (-37.3, 45.5) | 3.98 (2.16, 5.83) | -44.8 (-64.0, -16.5) | 4.38 (2.38, 6.41) | -27.7 (-52.8, 9.6) | 0.34 (-3.40, 4.24) |
|  | Paraguay | 6930455 (5699198, 8110997) | 71.3 | 217 (146, 310) | -11.2 (-49.5, 44.9) | 3.13 (2.10, 4.47) | -48.2 (-70.5, -15.4) | 3.44 (2.31, 4.93) | -14.9 (-51.2, 39.1) | 2.29 (-0.91, 5.59) |
|  | Peru | 33995397 (31120105, 36626226) | 56.4 | 820 (447, 1362) | -1.3 (-60.8, 92.4) | 2.41 (1.31, 4.01) | -36.9 (-75.0, 23.0) | 2.60 (1.41, 4.33) | -5.2 (-62.0, 86.0) | 0.66 (-0.39, 1.73) |
|  | Republic of Moldova | 3688191 (3095661, 4327374) | -17.1 | 56 (42, 72) | -74.9 (-81.6, -47.0) | 1.52 (1.15, 1.95) | -69.8 (-77.8, -36.1) | 2.99 (2.21, 3.92) | -47.9 (-62.2, 3.4) | -1.23 (-3.21, 0.79) |
|  | South Africa | 55588425 (49169682, 62724694) | 50.9 | 808 (585, 1054) | -15.6 (-45.4, 28.8) | 1.45 (1.05, 1.90) | -44.1 (-63.8, -14.7) | 1.58 (1.13, 2.08) | -26.9 (-52.6, 10.1) | -2.49 (-3.24, -1.75) |
|  | Sri Lanka | 21854452 (19445064, 24139979) | 26.9 | 559 (361, 797) | -51.1 (-74.7, -0.3) | 2.56 (1.65, 3.65) | -61.5 (-80.0, -21.5) | 3.38 (2.09, 4.88) | -47.0 (-73.4, 9.5) | -1.62 (-2.43, -0.80) |
|  | Thailand | 70111586 (61328965, 78909536) | 23.3 | 570 (391, 773) | -71.2 (-84.0, -48.0) | 0.81 (0.56, 1.10) | -76.6 (-87.0, -57.8) | 1.69 (1.05, 2.35) | -55.2 (-76.5, -16.2) | -2.40 (-4.54, -0.22) |
|  | Tunisia | 11571604 (10423733, 12757923) | 37.1 | 282 (194, 394) | -77.4 (-88.7, -48.0) | 2.44 (1.68, 3.41) | -83.5 (-91.8, -62.1) | 3.27 (2.24, 4.57) | -70.5 (-85.4, -32.1) | -0.94 (-2.65, 0.80) |
|  | Turkmenistan | 5083080 (4614049, 5544853) | 37.2 | 225 (115, 400) | -17.2 (-62.6, 36.5) | 4.42 (2.26, 7.88) | -39.7 (-72.7, -0.5) | 4.17 (2.14, 7.40) | -8.1 (-58.5, 51.0) | 1.91 (-0.94, 4.84) |
|  | Uruguay | 3436137 (3031175, 3876964) | 9.5 | 67 (49, 89) | -50.5 (-71.7, -29.2) | 1.95 (1.43, 2.60) | -54.8 (-74.2, -35.3) | 2.75 (2.01, 3.75) | -44.7 (-68.2, -19.2) | -0.47 (-2.62, 1.74) |
|  | Uzbekistan | 33677096 (25411001, 42319371) | 60.8 | 943 (464, 1344) | -10.6 (-49.5, 37.2) | 2.80 (1.38, 3.99) | -44.4 (-68.6, -14.7) | 2.70 (1.33, 3.86) | -15.5 (-51.8, 28.3) | 0.19 (-1.28, 1.68) |
| **Low-middle SDI** | Bolivia (Plurinational State of) | 12011750 (10641659, 13418191) | 87.1 | 776 (517, 1060) | 11.8 (-50.3, 175.1) | 6.46 (4.30, 8.82) | -40.2 (-73.4, 47.1) | 5.17 (3.46, 7.01) | -19.6 (-64.1, 92.8) | 0.21 (-1.30, 1.73) |
|  | Cameroon | 29101868 (24782960, 33604810) | 180.1 | 1171 (463, 2680) | 106.5 (21.2, 331.8) | 4.02 (1.59, 9.21) | -26.3 (-56.7, 54.2) | 2.85 (1.18, 6.38) | -1.9 (-40.8, 87.7) | -0.29 (-1.75, 1.20) |
|  | Congo | 5265846 (4507068, 6008799) | 115.4 | 105 (67, 180) | 7.4 (-41.8, 125.3) | 2.00 (1.28, 3.41) | -50.1 (-73.0, 4.6) | 1.59 (1.03, 2.69) | -32.2 (-62.3, 34.9) | -1.35 (-4.39, 1.79) |
|  | Democratic People's Republic of Korea | 26232861 (22628875, 29910477) | 24.6 | 694 (466, 1024) | -78.0 (-89.0, -48.0) | 2.65 (1.78, 3.90) | -82.4 (-91.2, -58.3) | 3.73 (2.44, 5.65) | -62.4 (-81.5, -11.7) | -0.47 (-1.20, 0.27) |
|  | Dominican Republic | 10881855 (9629751, 12279753) | 51.1 | 366 (209, 624) | -26.3 (-60.5, 36.4) | 3.37 (1.92, 5.74) | -51.2 (-73.9, -9.7) | 3.33 (1.90, 5.69) | -27.4 (-60.8, 32.7) | 0.51 (-1.24, 2.29) |
|  | El Salvador | 6256143 (5393104, 7102867) | 18.8 | 214 (95, 327) | -59.7 (-81.7, -9.3) | 3.43 (1.52, 5.23) | -66.1 (-84.6, -23.6) | 3.83 (1.69, 5.87) | -44.7 (-75.2, 25.4) | 0.50 (-2.73, 3.84) |
|  | Ghana | 31536232 (27445271, 35185758) | 110.0 | 902 (443, 1695) | 59.1 (-18.2, 264.8) | 2.86 (1.41, 5.37) | -24.2 (-61.0, 73.7) | 2.29 (1.14, 4.26) | 3.2 (-44.8, 113.9) | -0.36 (-1.68, 0.98) |
|  | Guatemala | 17776490 (14652057, 20925960) | 123.1 | 494 (244, 747) | 39.0 (-70.3, 216.9) | 2.78 (1.37, 4.20) | -37.7 (-86.7, 42.0) | 2.51 (1.23, 3.81) | 2.7 (-77.8, 129.6) | 1.62 (-0.14, 3.41) |
|  | Honduras | 9814396 (8823027, 10833410) | 108.4 | 265 (151, 445) | 65.9 (-34.6, 207.2) | 2.70 (1.54, 4.53) | -20.4 (-68.6, 47.4) | 2.41 (1.38, 4.05) | 19.4 (-52.2, 117.6) | 1.76 (-0.53, 4.09) |
|  | India | 1390706968 (1237773390, 1558771721) | 62.5 | 38152 (25486, 56481) | -27.5 (-59.7, 35.0) | 2.74 (1.83, 4.06) | -55.4 (-75.2, -17.0) | 3.20 (2.11, 4.79) | -28.0 (-59.9, 35.7) | -1.36 (-1.75, -0.98) |
|  | Kenya | 50227709 (43651058, 56751223) | 116.6 | 1283 (950, 1826) | 1.7 (-45.4, 103.9) | 2.55 (1.89, 3.64) | -53.0 (-74.8, -5.9) | 2.05 (1.51, 2.92) | -28.6 (-60.8, 35.9) | 0.28 (-0.89, 1.46) |
|  | Kyrgyzstan | 6535459 (5697805, 7315212) | 46.5 | 195 (148, 377) | -26.8 (-57.3, 46.0) | 2.98 (2.26, 5.76) | -50.0 (-70.8, -0.3) | 2.77 (2.11, 5.31) | -39.1 (-63.8, 21.3) | -0.33 (-2.07, 1.45) |
|  | Lao People's Democratic Republic | 7158250 (6469590, 7826130) | 72.4 | 501 (259, 855) | -37.8 (-67.9, 89.9) | 7.00 (3.62, 11.94) | -63.9 (-81.4, 10.2) | 6.18 (3.21, 10.50) | -45.2 (-71.5, 60.5) | -1.64 (-3.34, 0.09) |
|  | Mauritania | 4014273 (3561887, 4441999) | 94.3 | 76 (39, 145) | -12.1 (-58.4, 172.2) | 1.89 (0.98, 3.61) | -54.7 (-78.6, 40.1) | 1.48 (0.79, 2.81) | -35.6 (-69.0, 79.3) | -1.41 (-5.49, 2.85) |
|  | Mongolia | 3387589 (2977541, 3795389) | 57.3 | 137 (93, 226) | -18.8 (-58.0, 54.0) | 4.06 (2.73, 6.67) | -48.4 (-73.3, -2.1) | 3.66 (2.49, 5.92) | -30.6 (-63.4, 26.1) | 0.26 (-1.61, 2.18) |
|  | Morocco | 35952186 (32338676, 39427377) | 42.1 | 821 (367, 1701) | -58.2 (-85.3, 5.0) | 2.28 (1.02, 4.73) | -70.6 (-89.7, -26.2) | 2.73 (1.22, 5.69) | -48.9 (-81.8, 24.8) | -1.76 (-4.85, 1.43) |
|  | Myanmar | 54676901 (48907818, 60236349) | 33.0 | 3449 (1922, 5828) | -36.1 (-68.5, 65.9) | 6.31 (3.52, 10.66) | -51.9 (-76.3, 24.7) | 6.76 (3.75, 11.47) | -26.0 (-62.8, 87.2) | -1.60 (-2.17, -1.03) |
|  | Nicaragua | 6510365 (5514376, 7574280) | 67.5 | 212 (160, 275) | -27.3 (-58.4, 19.1) | 3.26 (2.46, 4.23) | -56.6 (-75.2, -28.9) | 3.27 (2.47, 4.25) | -26.1 (-57.6, 20.0) | 0.62 (-2.01, 3.32) |
|  | Nigeria | 214823786 (193132521, 236573636) | 138.2 | 11280 (3768, 24751) | 85.3 (23.3, 259.7) | 5.25 (1.75, 11.52) | -22.2 (-48.2, 51.0) | 3.32 (1.16, 7.21) | -7.1 (-38.0, 73.8) | -0.56 (-1.18, 0.07) |
|  | Palestine | 4956597 (4561835, 5329689) | 139.4 | 227 (170, 302) | -44.2 (-70.0, 15.8) | 4.58 (3.44, 6.10) | -76.7 (-87.5, -51.6) | 3.74 (2.81, 4.95) | -61.0 (-79.1, -20.5) | -1.88 (-5.40, 1.78) |
|  | Philippines | 112142764 (101580880, 121868835) | 77.2 | 5031 (3726, 6241) | 16.2 (-32.0, 91.0) | 4.49 (3.32, 5.57) | -34.4 (-61.6, 7.8) | 3.99 (2.96, 4.94) | -14.1 (-48.9, 39.7) | 0.27 (-0.27, 0.81) |
|  | Sudan | 40808425 (35356109, 46009715) | 102.0 | 3864 (1966, 6569) | -34.0 (-66.3, 92.8) | 9.47 (4.82, 16.10) | -67.3 (-83.3, -4.6) | 6.99 (3.60, 11.83) | -50.3 (-74.9, 45.7) | -0.72 (-1.73, 0.29) |
|  | Syrian Arab Republic | 14491247 (12173713, 16795620) | 12.4 | 474 (313, 680) | -79.7 (-89.4, -55.8) | 3.27 (2.16, 4.70) | -81.9 (-90.6, -60.7) | 4.03 (2.65, 5.83) | -59.3 (-79.3, -10.3) | -1.24 (-3.21, 0.77) |
|  | Tajikistan | 9492414 (8213913, 10674835) | 76.6 | 1171 (412, 1714) | 346.9 (-4.6, 579.6) | 12.33 (4.34, 18.06) | 153.1 (-46.0, 284.9) | 9.74 (3.44, 14.23) | 237.6 (-26.1, 404.1) | 2.15 (0.12, 4.21) |
|  | Timor-Leste | 1334823 (1208832, 1447383) | 70.5 | 75 (48, 110) | -32.6 (-70.0, 113.8) | 5.60 (3.60, 8.22) | -60.5 (-82.4, 25.4) | 4.21 (2.73, 6.14) | -41.7 (-73.4, 77.1) | -0.52 (-5.62, 4.86) |
|  | Venezuela (Bolivarian Republic of) | 28068986 (24769383, 31423834) | 49.1 | 903 (623, 1266) | -30.8 (-60.0, 4.3) | 3.22 (2.22, 4.51) | -53.6 (-73.1, -30.0) | 3.78 (2.61, 5.33) | -25.1 (-56.0, 12.4) | -0.53 (-1.39, 0.34) |
|  | Viet Nam | 96372928 (83066414, 109007012) | 41.8 | 2089 (1413, 3072) | -41.4 (-68.0, 12.3) | 2.17 (1.47, 3.19) | -58.7 (-77.4, -20.8) | 2.91 (1.92, 4.37) | -29.4 (-61.5, 35.6) | 0.06 (-0.48, 0.60) |
|  | Zambia | 18237683 (15885855, 20473278) | 129.6 | 898 (533, 1387) | 1.3 (-56.1, 241.0) | 4.93 (2.92, 7.61) | -55.9 (-80.9, 48.5) | 3.25 (1.98, 4.95) | -41.3 (-74.0, 83.1) | -0.49 (-2.22, 1.27) |
| **Low SDI** | Afghanistan | 38277536 (26161826, 50468583) | 235.2 | 6477 (2845, 10934) | 56.8 (-11.5, 336.5) | 16.92 (7.43, 28.56) | -53.2 (-73.6, 30.2) | 9.69 (4.29, 16.17) | -47.8 (-70.4, 44.1) | -0.57 (-1.49, 0.36) |
|  | Angola | 30138521 (27054840, 33116641) | 192.1 | 1180 (648, 2061) | 76.1 (-19.8, 490.9) | 3.91 (2.15, 6.84) | -39.7 (-72.5, 102.3) | 2.40 (1.37, 4.11) | -26.9 (-66.1, 122.7) | -0.48 (-1.96, 1.03) |
|  | Bangladesh | 159259850 (141199841, 177852818) | 46.0 | 5752 (2899, 10849) | -50.3 (-81.7, 28.8) | 3.61 (1.82, 6.81) | -66.0 (-87.5, -11.8) | 4.17 (2.09, 7.93) | -36.9 (-76.6, 60.0) | -1.41 (-1.83, -0.98) |
|  | Benin | 12665751 (11316886, 13983186) | 161 | 740 (267, 1561) | 62.4 (-11.9, 296.8) | 5.85 (2.11, 12.32) | -37.8 (-66.2, 52.0) | 3.32 (1.28, 6.84) | -26.5 (-59.6, 64.5) | -0.84 (-3.24, 1.62) |
|  | Burkina Faso | 22691773 (19383320, 26173262) | 137.4 | 1958 (502, 4756) | 125.8 (28.2, 427.1) | 8.63 (2.21, 20.96) | -4.9 (-46.0, 122.0) | 4.73 (1.29, 11.42) | 5.5 (-39.3, 128.9) | -0.11 (-1.88, 1.69) |
|  | Burundi | 11934361 (10304612, 13532061) | 114.2 | 698 (337, 1393) | 9.2 (-48.0, 265.3) | 5.85 (2.82, 11.67) | -49.0 (-75.7, 70.5) | 3.41 (1.70, 6.66) | -40.4 (-71.5, 84.2) | -1.00 (-2.98, 1.03) |
|  | Cambodia | 16603118 (14206180, 18867660) | 60.0 | 838 (506, 1265) | -47.2 (-71.3, 41.9) | 5.05 (3.04, 7.62) | -67.0 (-82.1, -11.3) | 4.76 (2.86, 7.17) | -43.4 (-68.7, 42.8) | -1.31 (-2.45, -0.16) |
|  | Central African Republic | 5299863 (4459289, 6192557) | 93.1 | 258 (85, 587) | 45.0 (-10.5, 188.7) | 4.87 (1.60, 11.08) | -24.9 (-53.7, 49.5) | 3.03 (1.05, 6.79) | -11.5 (-43.9, 64.1) | -0.44 (-3.18, 2.39) |
|  | Chad | 16398860 (14327391, 18680096) | 172.3 | 995 (253, 2446) | 109.3 (28.8, 407.5) | 6.07 (1.54, 14.92) | -23.1 (-52.7, 86.4) | 2.98 (0.84, 7.14) | -18.6 (-49.7, 79.0) | -0.15 (-2.39, 2.14) |
|  | Côte d'Ivoire | 26171532 (23573125, 28872979) | 114.1 | 1037 (476, 2039) | 20.1 (-34.7, 193.5) | 3.96 (1.82, 7.79) | -43.9 (-69.5, 37.1) | 2.63 (1.26, 5.02) | -27.0 (-60.2, 60.9) | -0.61 (-2.07, 0.87) |
|  | Democratic Republic of the Congo | 87670444 (61748630, 112590350) | 127.2 | 2587 (1479, 4573) | -10.0 (-60.4, 189.4) | 2.95 (1.69, 5.22) | -60.4 (-82.6, 27.4) | 1.98 (1.16, 3.42) | -45.7 (-76.0, 57.2) | -0.55 (-1.41, 0.31) |
|  | Djibouti | 1202797 (1050469, 1362651) | 147.5 | 51 (28, 85) | 52.7 (-17.4, 221.8) | 4.22 (2.34, 7.06) | -38.3 (-66.6, 30.0) | 3.19 (1.79, 5.31) | -9.3 (-50.1, 83.2) | -0.19 (-7.34, 7.52) |
|  | Eritrea | 6711213 (4780648, 8595693) | 123.6 | 256 (139, 421) | 36.8 (-34.5, 269.4) | 3.82 (2.07, 6.27) | -38.8 (-70.7, 65.2) | 2.82 (1.56, 4.62) | -17.8 (-60.0, 108.3) | -0.17 (-2.89, 2.63) |
|  | Ethiopia | 107591164 (92024285, 122776186) | 109.4 | 4186 (2237, 6984) | -25.1 (-61.9, 142.4) | 3.89 (2.08, 6.49) | -64.2 (-81.8, 15.8) | 2.53 (1.41, 4.16) | -53.3 (-76.4, 41.5) | -2.31 (-3.01, -1.59) |
|  | Gambia | 2245866 (2028000, 2476236) | 126.4 | 60 (35, 101) | 12.7 (-50.5, 214.6) | 2.66 (1.55, 4.51) | -50.2 (-78.1, 39.0) | 1.87 (1.12, 3.16) | -29.7 (-68.6, 81.8) | 1.13 (-4.08, 6.63) |
|  | Guinea | 12643149 (11365936, 13951889) | 104.4 | 725 (231, 1667) | 13.0 (-38.9, 233.5) | 5.73 (1.82, 13.19) | -44.7 (-70.1, 63.2) | 3.41 (1.17, 7.72) | -31.5 (-62.7, 87.2) | -0.05 (-2.20, 2.15) |
|  | Guinea-Bissau | 1901191 (1666339, 2146164) | 88.7 | 72 (31, 141) | -16.8 (-58.1, 159.5) | 3.79 (1.64, 7.43) | -55.9 (-77.8, 37.5) | 2.59 (1.18, 4.99) | -42.9 (-71.2, 60.0) | -0.67 (-5.23, 4.11) |
|  | Haiti | 12402099 (10373558, 14713362) | 95.1 | 1492 (652, 2419) | 12.0 (-38.7, 118.8) | 12.03 (5.26, 19.51) | -42.6 (-68.6, 12.1) | 9.85 (4.27, 16.17) | -22.4 (-56.9, 42.2) | -0.45 (-1.33, 0.44) |
|  | Liberia | 4789907 (4131045, 5420863) | 143.9 | 125 (57, 244) | -43.8 (-76.9, 214.8) | 2.61 (1.20, 5.08) | -76.9 (-90.5, 29.1) | 2.01 (0.96, 3.88) | -68.4 (-86.9, 55.3) | -1.04 (-4.66, 2.72) |
|  | Madagascar | 26690344 (20373798, 32844324) | 123.3 | 999 (591, 1645) | -7.3 (-57.1, 138.5) | 3.74 (2.21, 6.16) | -58.5 (-80.8, 6.8) | 2.57 (1.56, 4.20) | -45.8 (-74.8, 32.5) | -1.12 (-2.60, 0.38) |
|  | Malawi | 18442238 (17149774, 19745167) | 93.0 | 812 (470, 1307) | -33.3 (-70.7, 127.7) | 4.40 (2.55, 7.09) | -65.4 (-84.8, 18.0) | 3.20 (1.90, 5.09) | -48.7 (-77.4, 63.8) | -0.64 (-2.34, 1.09) |
|  | Mali | 21917467 (19126101, 24868428) | 152.7 | 1577 (434, 3848) | 82.2 (2.6, 442.5) | 7.20 (1.98, 17.55) | -27.9 (-59.4, 114.7) | 3.80 (1.13, 9.12) | -21.2 (-55.8, 118.2) | -0.81 (-2.62, 1.03) |
|  | Mozambique | 29528037 (27057907, 31808337) | 125.9 | 2101 (1019, 3692) | 62.2 (-29.4, 472.9) | 7.12 (3.45, 12.50) | -28.2 (-68.7, 153.6) | 4.18 (2.13, 7.18) | -20.0 (-64.7, 163.2) | 1.39 (-1.44, 4.30) |
|  | Nepal | 30416382 (26611408, 34238807) | 55.7 | 914 (541, 1448) | -45.8 (-76.7, 53.7) | 3.00 (1.78, 4.76) | -65.2 (-85.0, -1.3) | 3.03 (1.78, 4.82) | -38.2 (-73.3, 71.7) | -1.51 (-2.51, -0.51) |
|  | Niger | 23295353 (20797660, 25931631) | 190.4 | 1427 (312, 3495) | 82.2 (-3.0, 489.1) | 6.12 (1.34, 15.00) | -37.2 (-66.6, 102.9) | 2.93 (0.70, 7.05) | -34.7 (-65.5, 98.0) | -0.76 (-2.81, 1.34) |
|  | Pakistan | 224062847 (207077327, 241657356) | 98.6 | 11519 (6948, 18709) | 40.5 (-17.3, 152.6) | 5.14 (3.10, 8.35) | -29.2 (-58.3, 27.2) | 3.86 (2.33, 6.25) | -4.7 (-42.8, 65.2) | 0.24 (-0.51, 0.99) |
|  | Papua New Guinea | 9866614 (8688195, 10952388) | 141.4 | 960 (342, 1917) | 116.4 (43.2, 293.9) | 9.73 (3.47, 19.43) | -10.4 (-40.7, 63.2) | 6.67 (2.50, 13.10) | -3.5 (-35.5, 64.9) | 0.14 (-1.35, 1.66) |
|  | Rwanda | 12688117 (11344258, 14076210) | 76.9 | 581 (311, 977) | -15.7 (-61.5, 183.2) | 4.58 (2.45, 7.70) | -52.3 (-78.2, 60.1) | 3.54 (1.92, 5.90) | -30.7 (-67.8, 117.6) | -1.97 (-3.75, -0.16) |
|  | Senegal | 15134067 (13503992, 16852509) | 98.6 | 516 (258, 945) | 4.0 (-53.9, 195.0) | 3.41 (1.70, 6.25) | -47.6 (-76.8, 48.6) | 2.44 (1.25, 4.42) | -28.6 (-67.8, 87.8) | -0.35 (-2.35, 1.70) |
|  | Sierra Leone | 8284755 (7526348, 9080218) | 126.9 | 646 (178, 1515) | 39.9 (-22.9, 327.5) | 7.80 (2.15, 18.29) | -38.3 (-66.0, 88.4) | 5.14 (1.48, 11.91) | -24.6 (-57.9, 116.2) | 0.05 (-2.78, 2.96) |
|  | Somalia | 20343112 (15201445, 25703627) | 184.6 | 1397 (565, 2749) | 125.0 (20.4, 457.1) | 6.87 (2.78, 13.51) | -20.9 (-57.7, 95.7) | 3.72 (1.59, 7.19) | -19.7 (-56.4, 89.6) | -0.13 (-3.79, 3.66) |
|  | South Sudan | 9282963 (8050826, 10613472) | 58.4 | 621 (235, 1248) | 5.8 (-38.9, 154.4) | 6.69 (2.53, 13.45) | -33.2 (-61.4, 60.6) | 3.88 (1.56, 7.66) | -28.6 (-58.4, 66.5) | -0.42 (-2.96, 2.18) |
|  | Togo | 7921527 (6943022, 8897862) | 116.3 | 211 (98, 434) | -0.4 (-44.5, 142.0) | 2.66 (1.24, 5.48) | -54.0 (-74.4, 11.9) | 1.96 (0.95, 3.97) | -36.0 (-63.7, 43.4) | -0.70 (-3.43, 2.11) |
|  | Uganda | 41117856 (37023696, 44955511) | 137.5 | 2247 (1064, 4089) | 45.5 (-24.9, 310.0) | 5.46 (2.59, 9.94) | -38.7 (-68.4, 72.7) | 3.19 (1.56, 5.67) | -19.9 (-58.3, 108.5) | -0.21 (-1.55, 1.14) |
|  | United Republic of Tanzania | 56736116 (50495485, 63230443) | 119.0 | 4096 (2121, 6870) | 37.1 (-29.8, 280.7) | 7.22 (3.74, 12.11) | -37.4 (-68.0, 73.8) | 4.39 (2.38, 7.24) | -23.7 (-60.8, 101.6) | -0.21 (-1.22, 0.81) |
|  | Yemen | 31502896 (26596895, 36775107) | 129.5 | 3082 (1873, 4751) | -10.1 (-51.7, 140.9) | 9.78 (5.94, 15.08) | -60.8 (-78.9, 5.0) | 7.04 (4.29, 10.85) | -38.6 (-66.7, 61.5) | -0.03 (-1.23, 1.19) |
|  | Zimbabwe | 15010852 (13317204, 16650709) | 45.2 | 405 (244, 612) | 40.0 (-15.9, 126.4) | 2.70 (1.63, 4.08) | -3.6 (-42.1, 55.9) | 2.04 (1.26, 3.02) | 15.5 (-28.3, 81.9) | 0.91 (-0.68, 2.52) |

**Notes:**

All-age mortality=crude mortality rate.

Age-standardized mortality rate is computed by direct standardization with global standard population in GBD 2019.

† Net drifts are estimates derived from the age-period-cohort model and denotes overall annual percent change in mortality.

*Parenthesis for all GBD health estimate indicates 95% uncertainty intervals; parenthesis for net drift indicates 95% confidence intervals.

SDI= Socio-demographic Index; APC= age-period-cohort


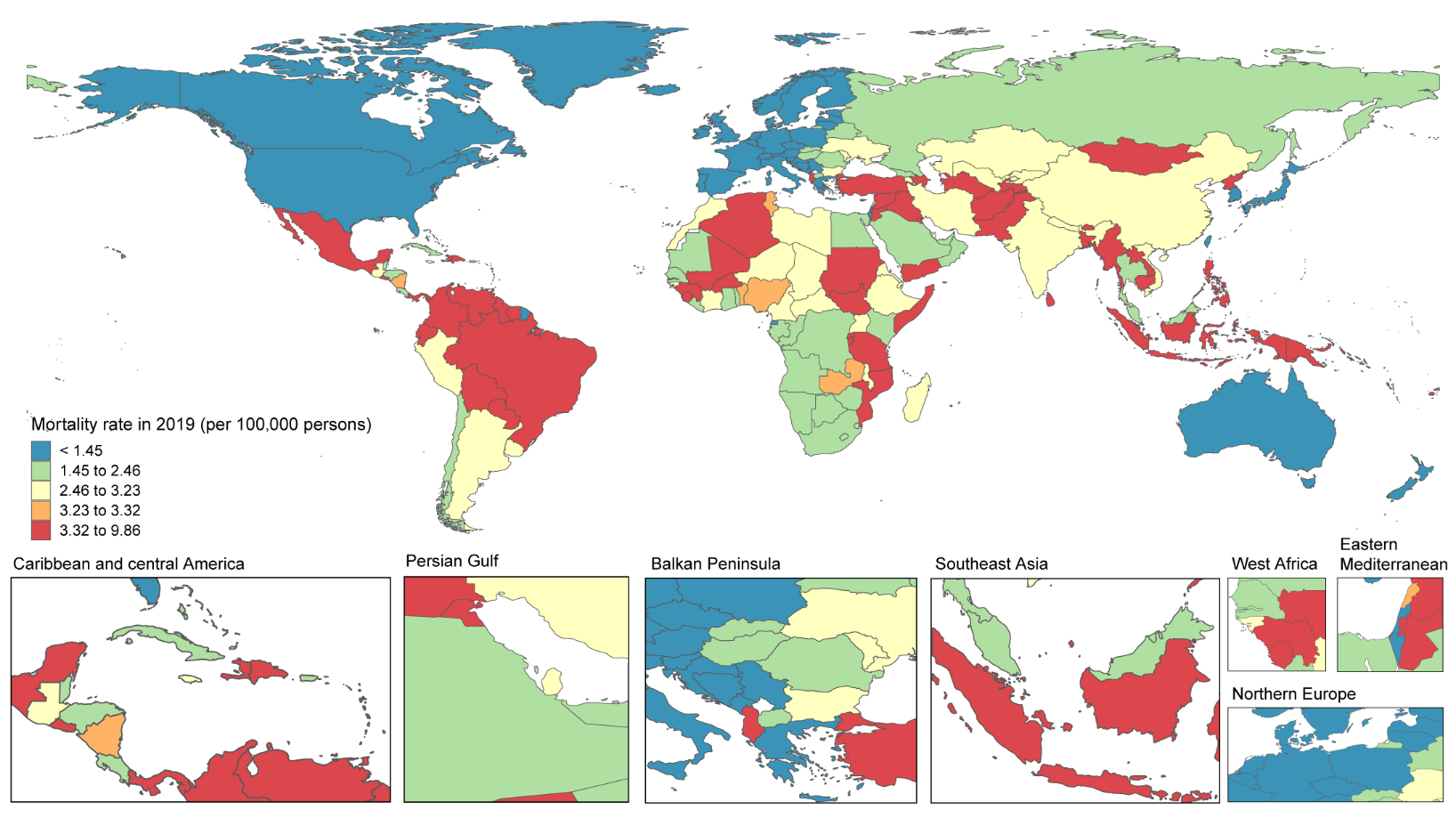
**Figure S1. The world map of age-standardized mortality for congenital heart disease, 1990-2019**

Note: In 2019, global age-standardized mortality rate was 3.23 (95%UI: 2.64-3.92) per 100,000 population.

**Figure S2. The local drifts and age distribution of deaths for congenital heart disease under 25 years of age across SDI quintile, 1990-2019**


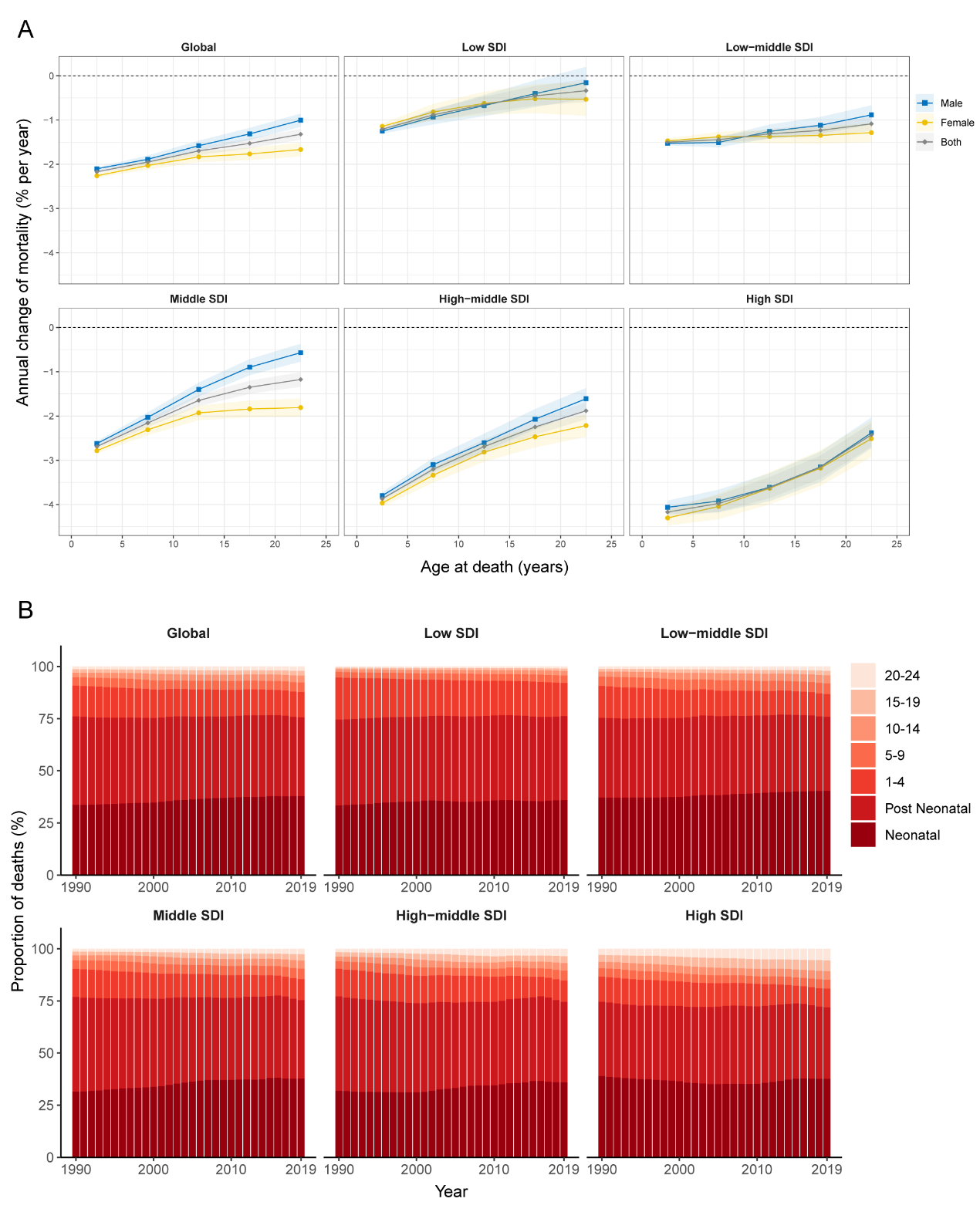


(A) The dots and shaded areas indicate the values of local drift (annual percentage change in mortality) and its 95% CIs for congenital heart disease in younger age groups (0~4 to 20~24 years). (B) Age distribution of deaths is represented as temporal change in the relative proportion of deaths across these age groups during 1990-2019. SDI=Socio-demographic Index.

**Figure S3. The local drifts of congenital heart disease mortality in high-SDI countries, 1990-2019**


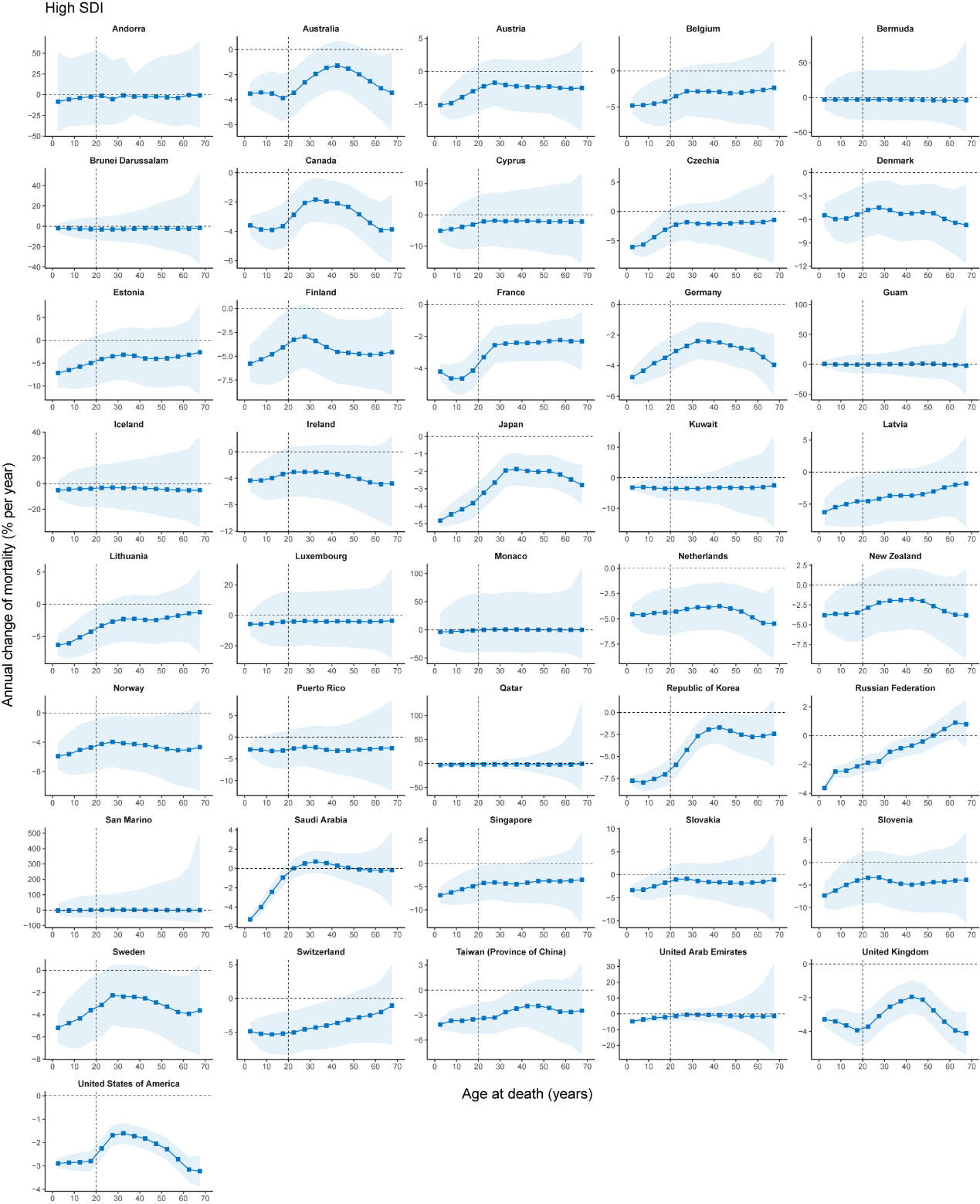


The dots and shaded areas indicate the values of local drift (annual percentage change in mortality) and its 95% CIs for congenital heart disease in 14 age groups (0~4 to 65~69 years). SDI=Socio-demographic Index.

**Figure S4. The local drifts of congenital heart disease mortality in high-middle SDI countries, 1990-2019**


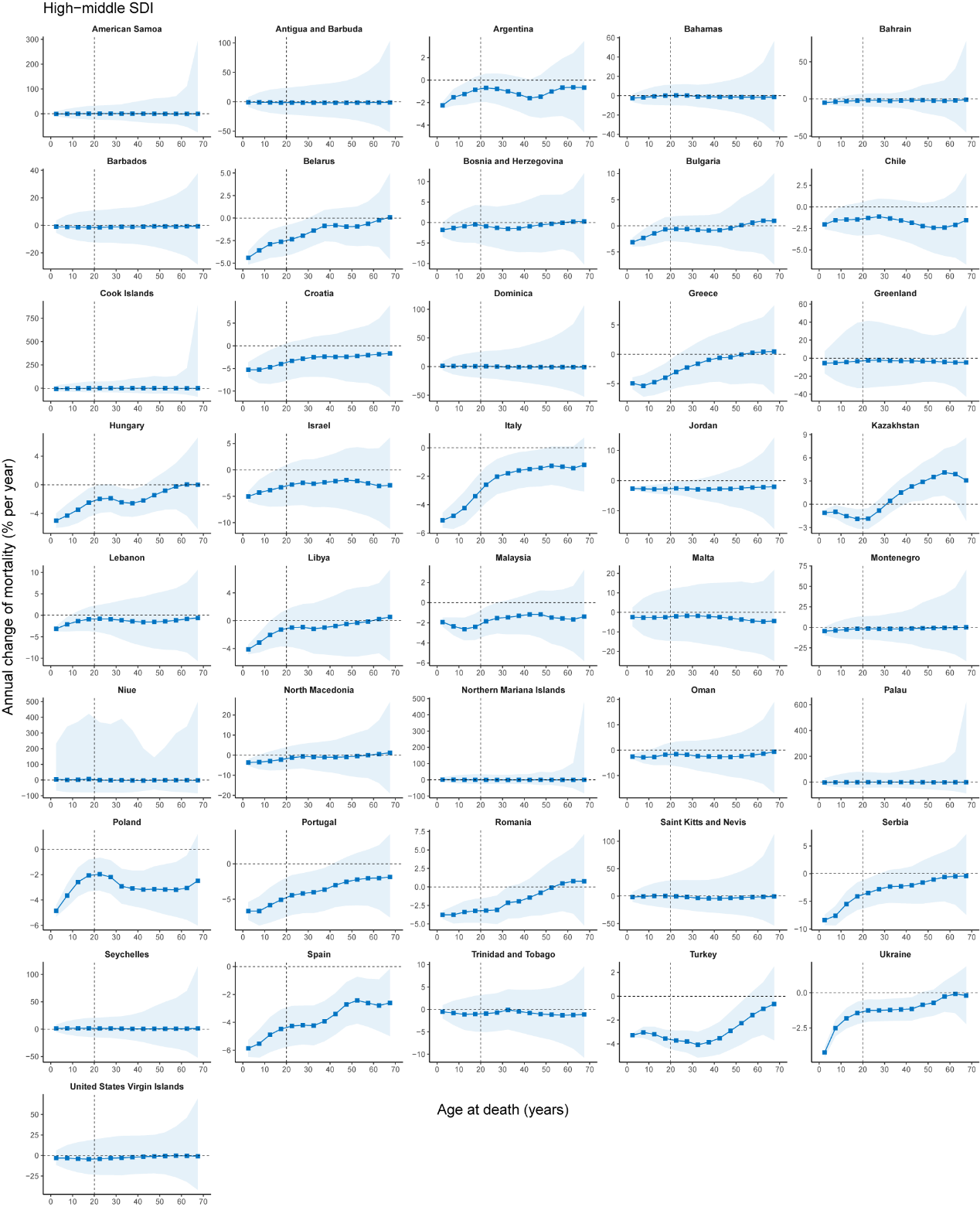


The dots and shaded areas indicate the values of local drift (annual percentage change in mortality) and its 95% CIs for congenital heart disease in 14 age groups (0~4 to 65~69 years). SDI=Socio-demographic Index.

**Figure S5. The local drifts of congenital heart disease mortality in middle-SDI countries, 1990-2019**


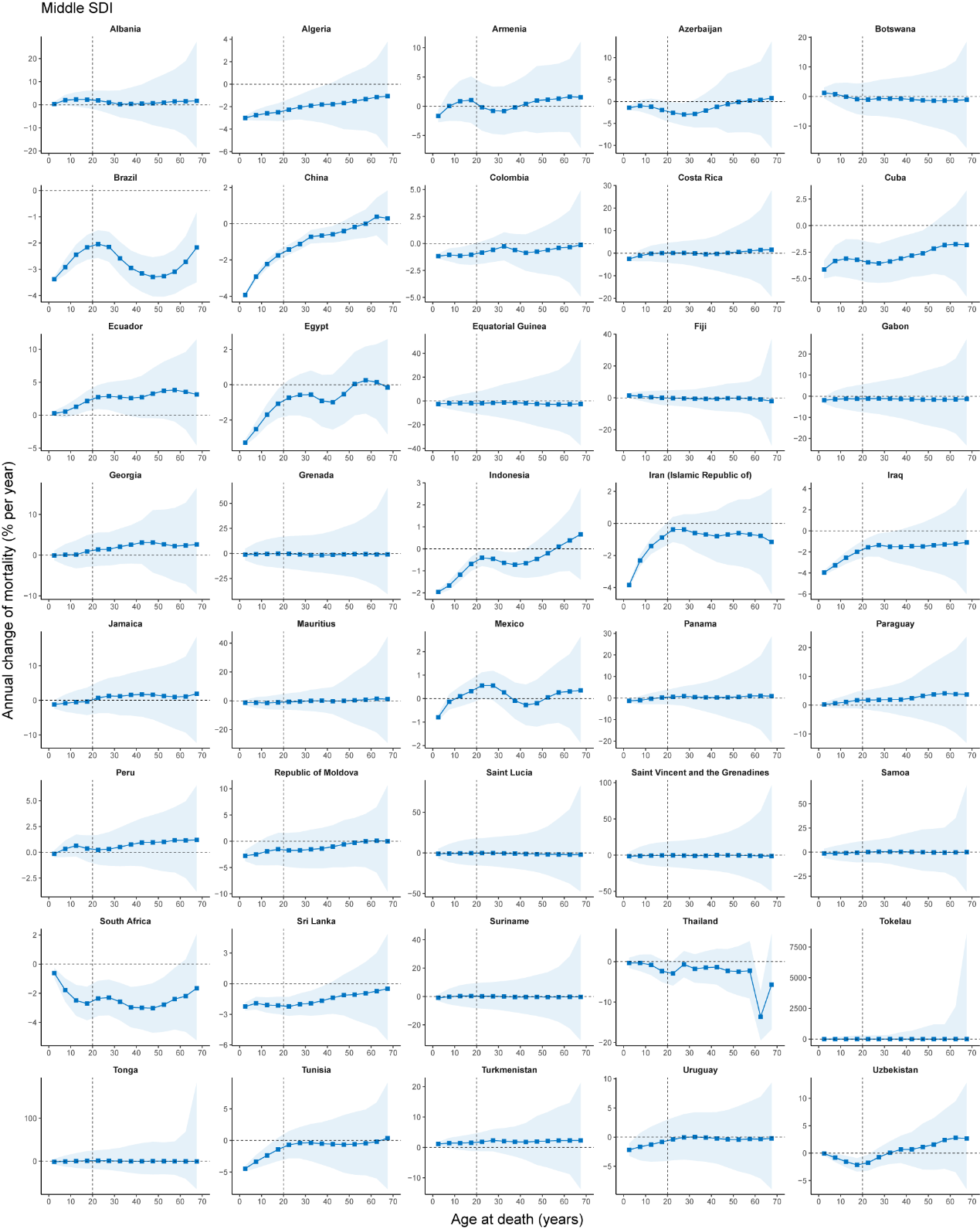


The dots and shaded areas indicate the values of local drift (annual percentage change in mortality) and its 95% CIs for congenital heart disease in 14 age groups (0~4 to 65~69 years). SDI=Socio-demographic Index.

**Figure S6. The local drifts of congenital heart disease mortality in low-middle SDI countries, 1990-2019**


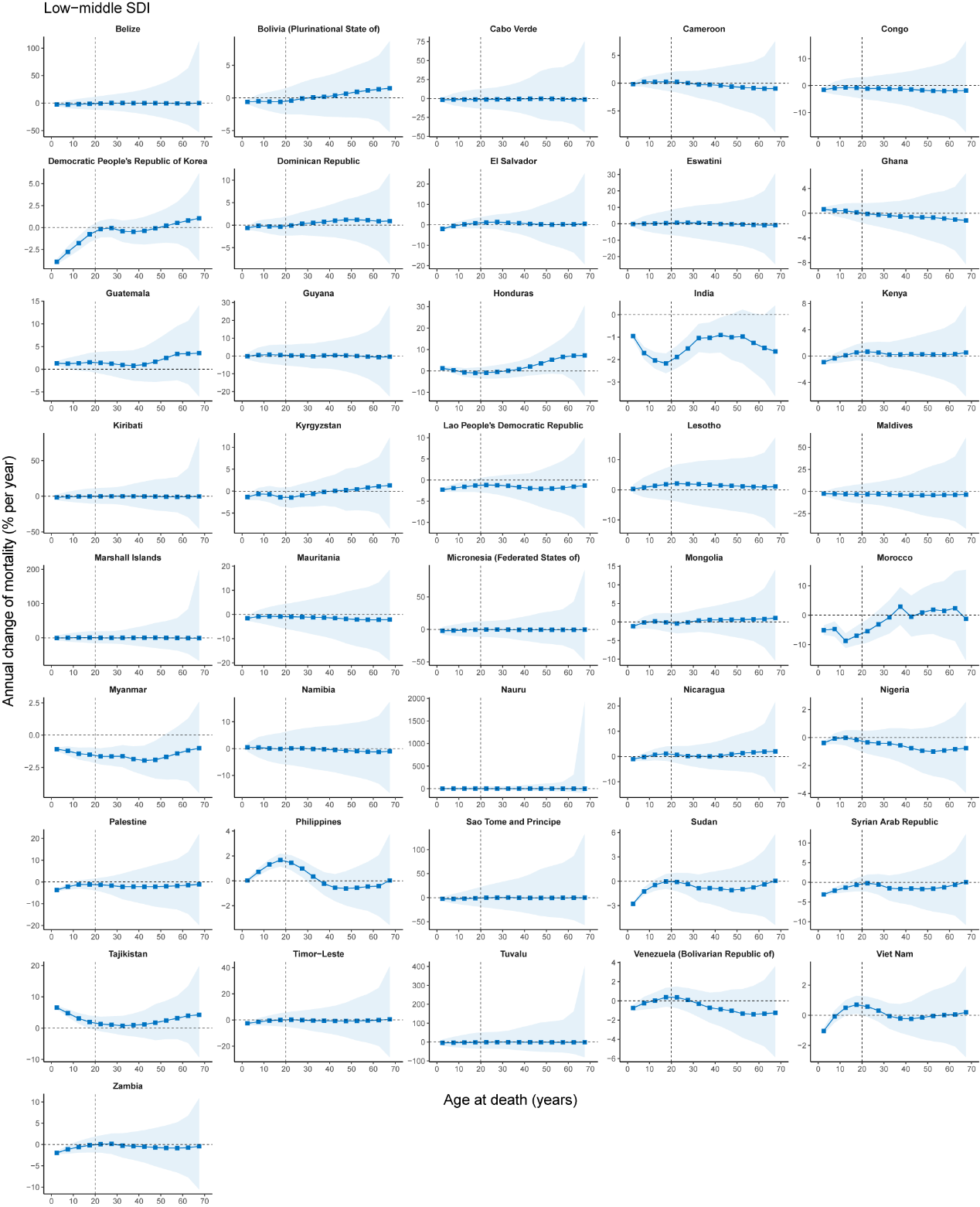


The dots and shaded areas indicate the values of local drift (annual percentage change in mortality) and its 95% CIs for congenital heart disease in 14 age groups (0~4 to 65~69 years). SDI=Socio-demographic Index.

**Figure S7. The local drifts of congenital heart disease mortality in low-SDI countries, 1990-2019**


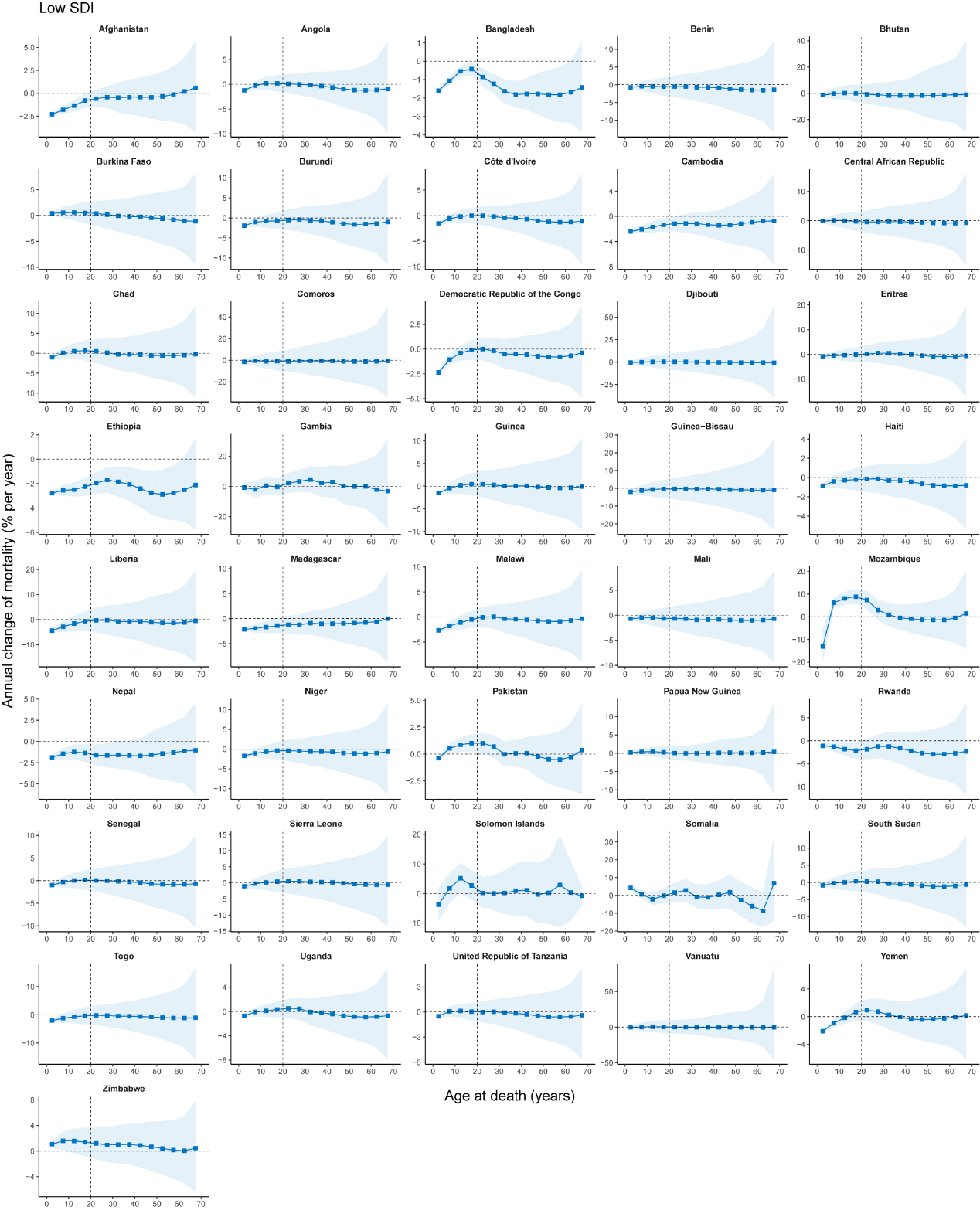


The dots and shaded areas indicate the values of local drift (annual percentage change in mortality) and its 95% CIs for congenital heart disease in 14 age groups (0~4 to 65~69 years). SDI=Socio-demographic Index.


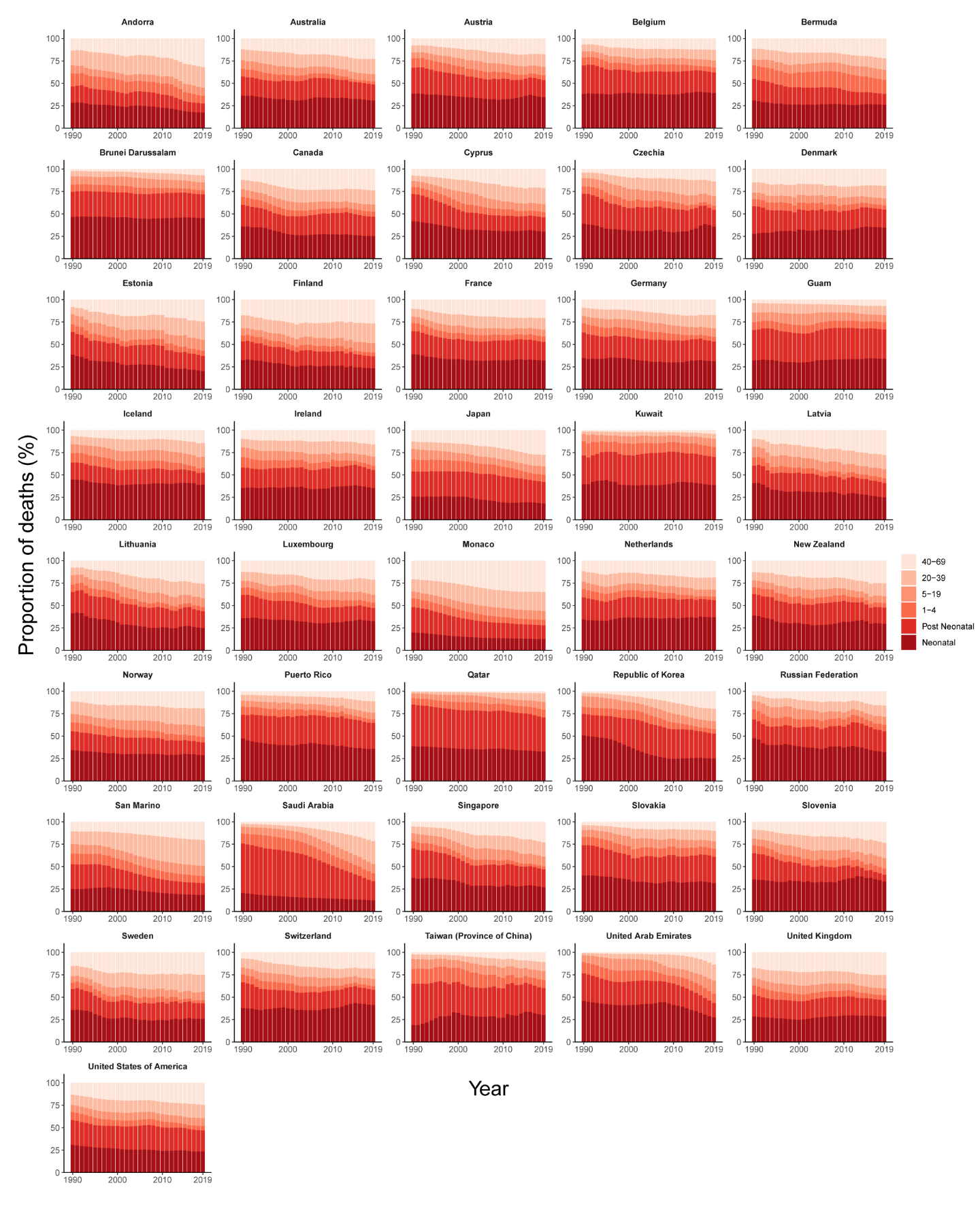
**Figure S8.** **Age distribution of deaths from congenital heart disease in high-SDI countries, 1990-2019**

Age distribution of deaths is represented as temporal change in the relative proportion of deaths across age groups (neonatal, post neonatal, 1-4, 5-19, 20-39, 40-69 years) during 1990-2019. Neonatal: <30 days; Post neonatal: 30 days-1year.

SDI=Socio-demographic Index.


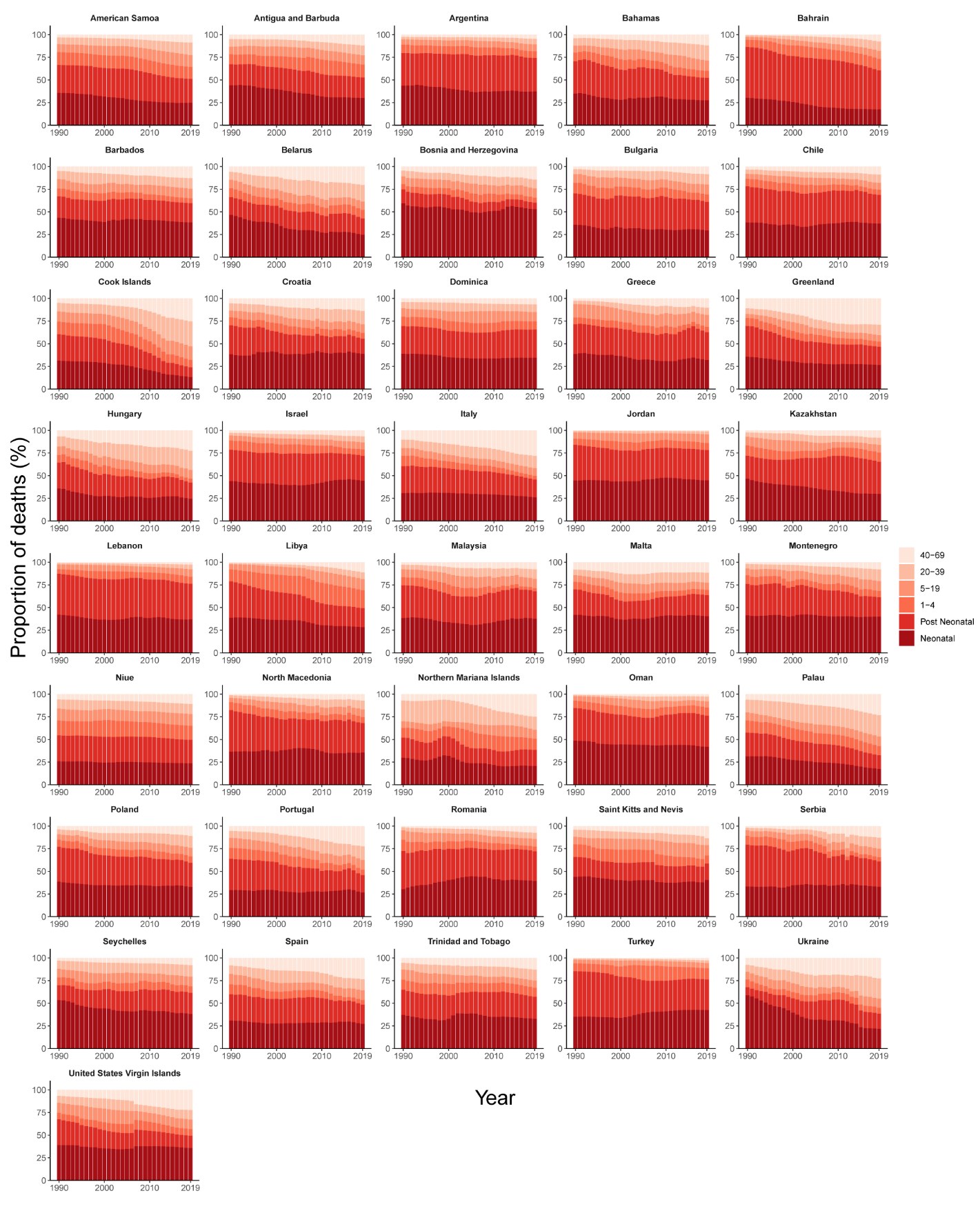
**Figure S9. Age distribution of deaths from congenital heart disease in high-middle SDI countries, 1990-2019**

Age distribution of deaths is represented as temporal change in the relative proportion of deaths across age groups (neonatal, post neonatal, 1-4, 5-19, 20-39, 40-69 years) during 1990-2019. Neonatal: <30 days; Post neonatal: 30 days-1year.

SDI=Socio-demographic Index.


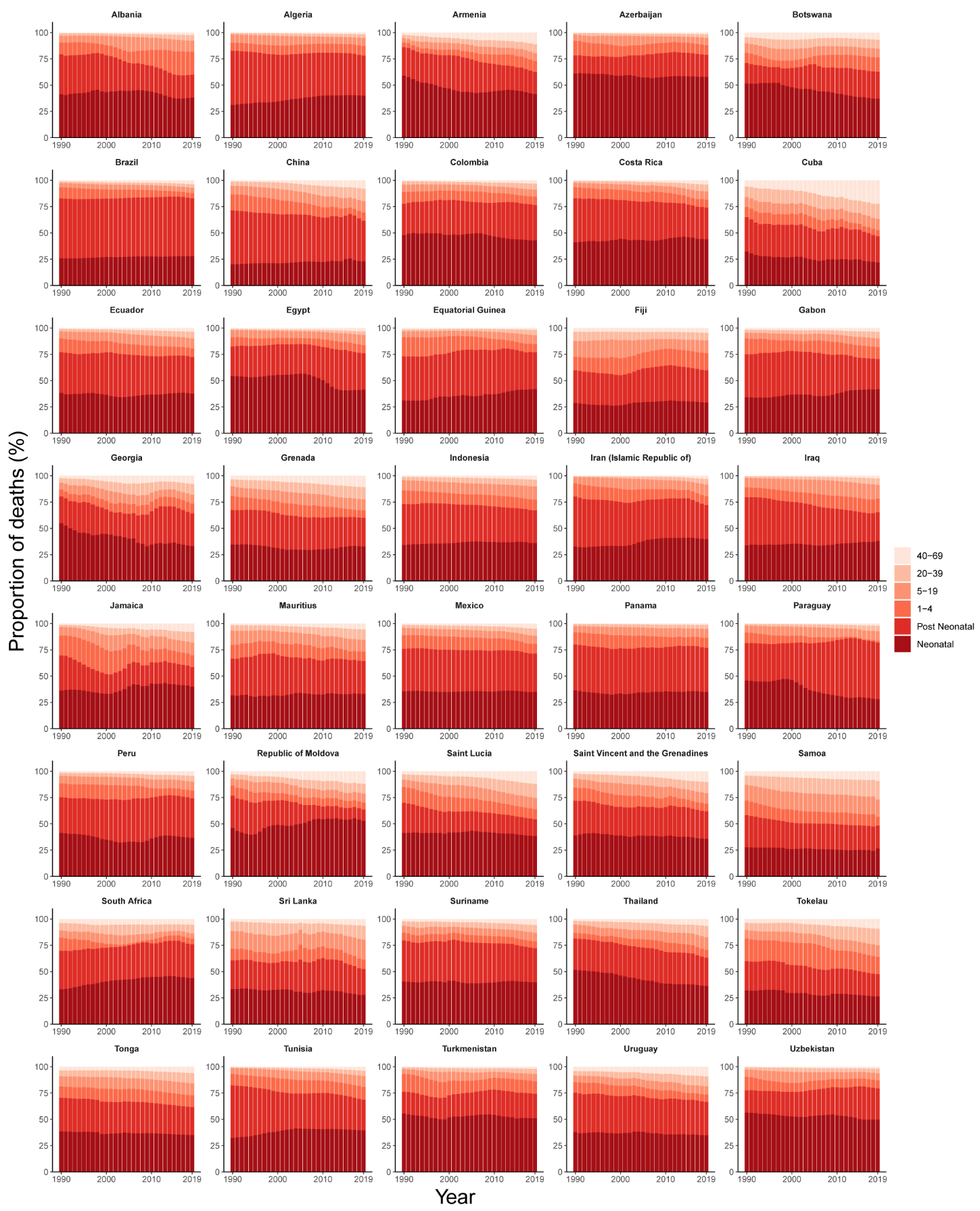
**Figure S10. Age distribution of deaths from congenital heart disease in middle-SDI countries, 1990-2019**

Age distribution of deaths is represented as temporal change in the relative proportion of deaths across age groups (neonatal, post neonatal, 1-4, 5-19, 20-39, 40-69 years) during 1990-2019. Neonatal: <30 days; Post neonatal: 30 days-1year.

SDI=Socio-demographic Index.


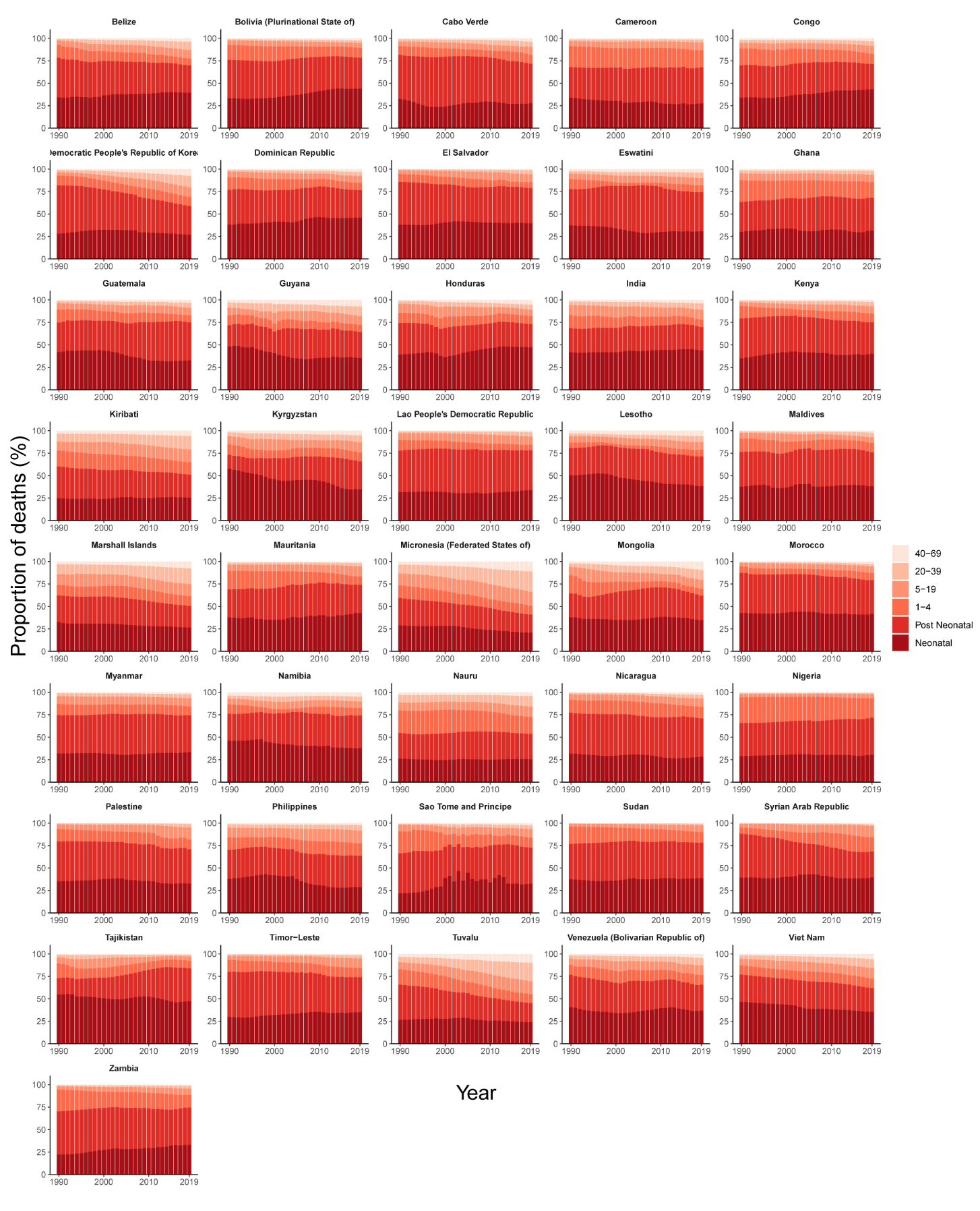
**Figure S11. Age distribution of deaths from congenital heart disease in low-middle SDI countries, 1990-2019**

Age distribution of deaths is represented as temporal change in the relative proportion of deaths across age groups (neonatal, post neonatal, 1-4, 5-19, 20-39, 40-69 years) during 1990-2019. Neonatal: <30 days; Post neonatal: 30 days-1year.

SDI=Socio-demographic Index.


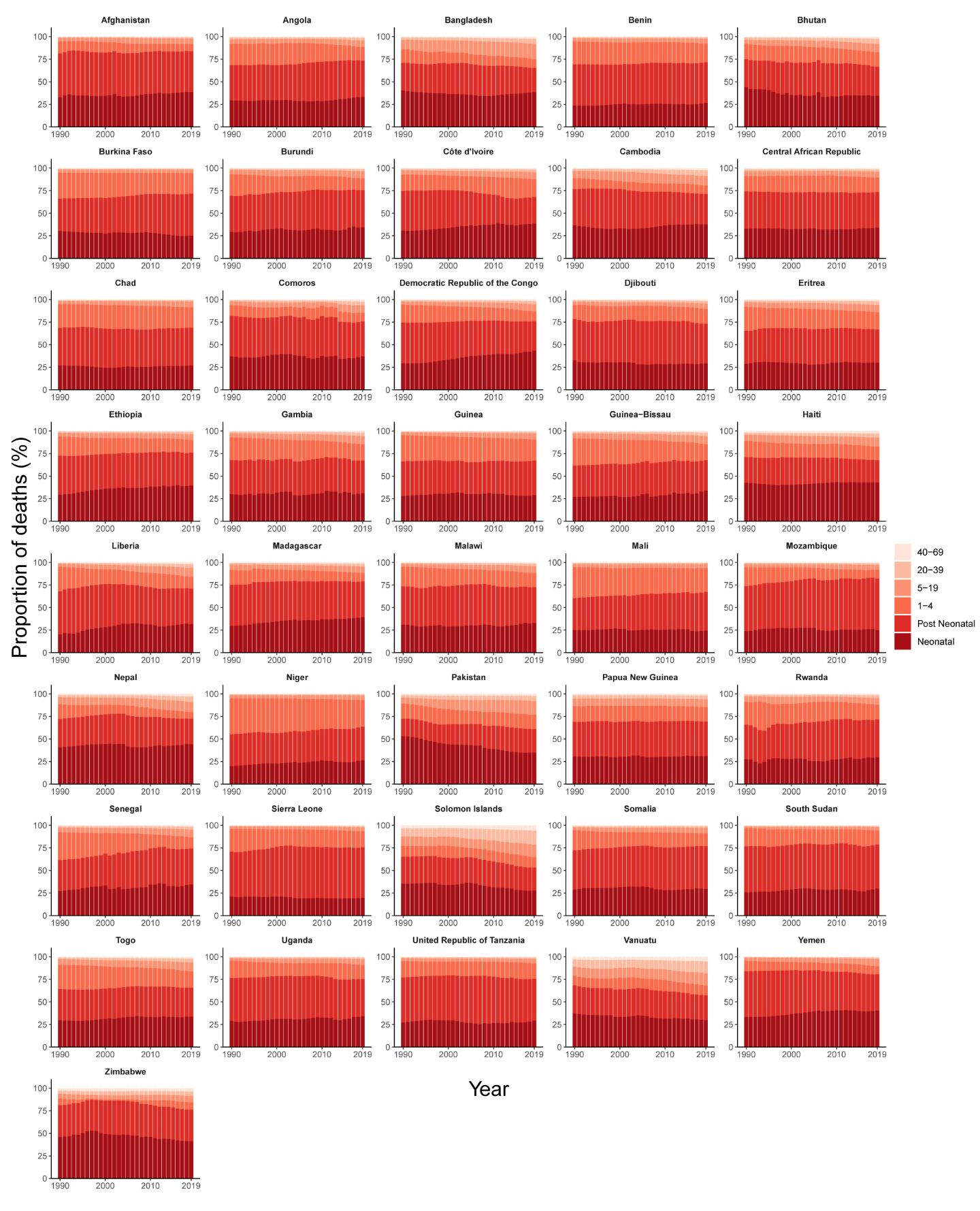
**Figure S12. Age distribution of deaths from congenital heart disease in low-SDI countries, 1990-2019**

Age distribution of deaths is represented as temporal change in the relative proportion of deaths across age groups (neonatal, post neonatal, 1-4, 5-19, 20-39, 40-69 years) during 1990-2019. Neonatal: <30 days; Post neonatal: 30 days-1year.

SDI=Socio-demographic Index.

**Figure S13. Age effects on congenital heart disease mortality in high-SDI countries**


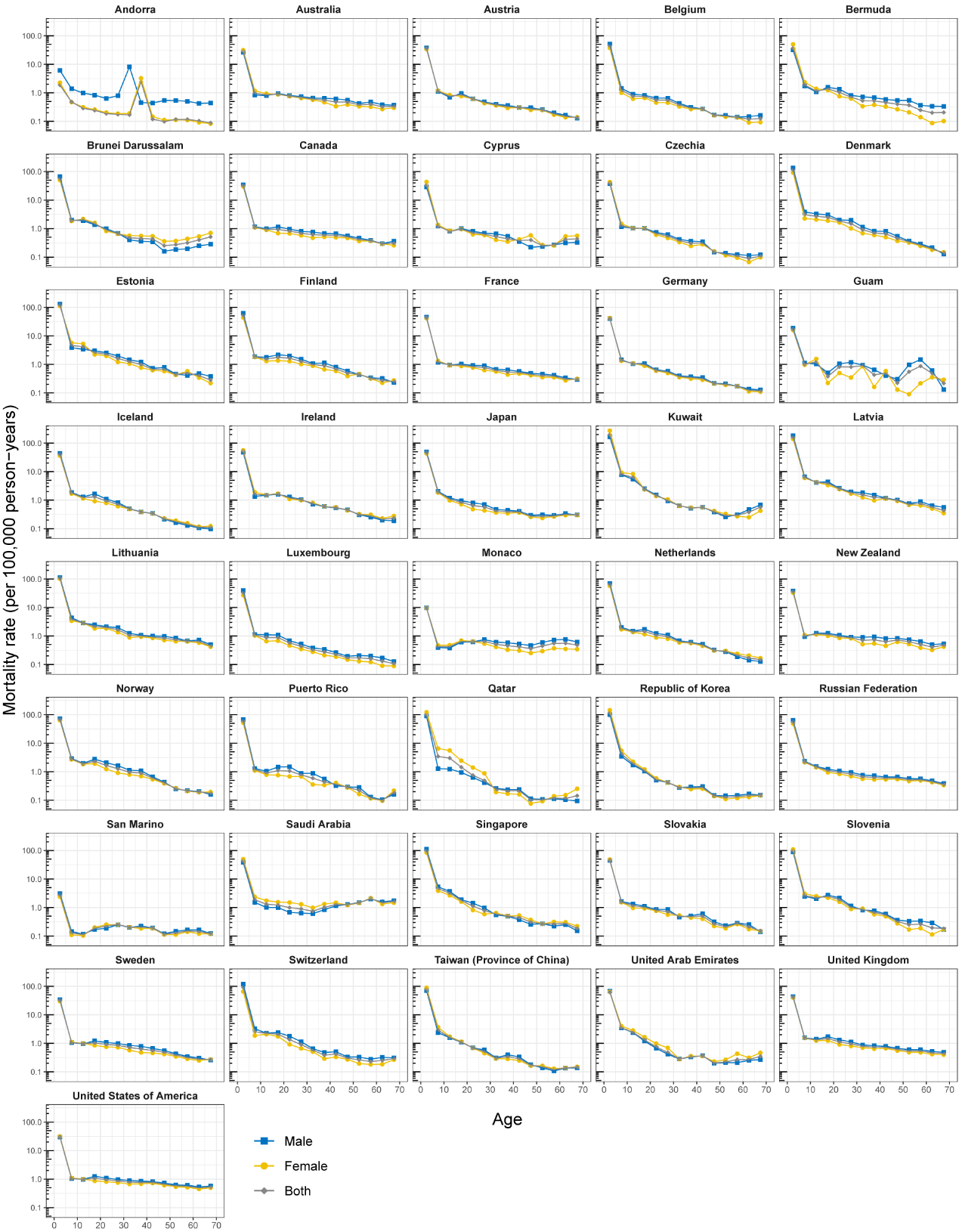


Age effects indicate age-associated natural history and are shown by the fitted longitudinal age curves of mortality (per 100000 person-years) adjusted for period deviations, with the dots and shaded areas denoting mortality rates with 95% CIs. SDI=Socio-demographic Index.

**Figure S14. Age effects on congenital heart disease mortality in high-middle SDI countries**


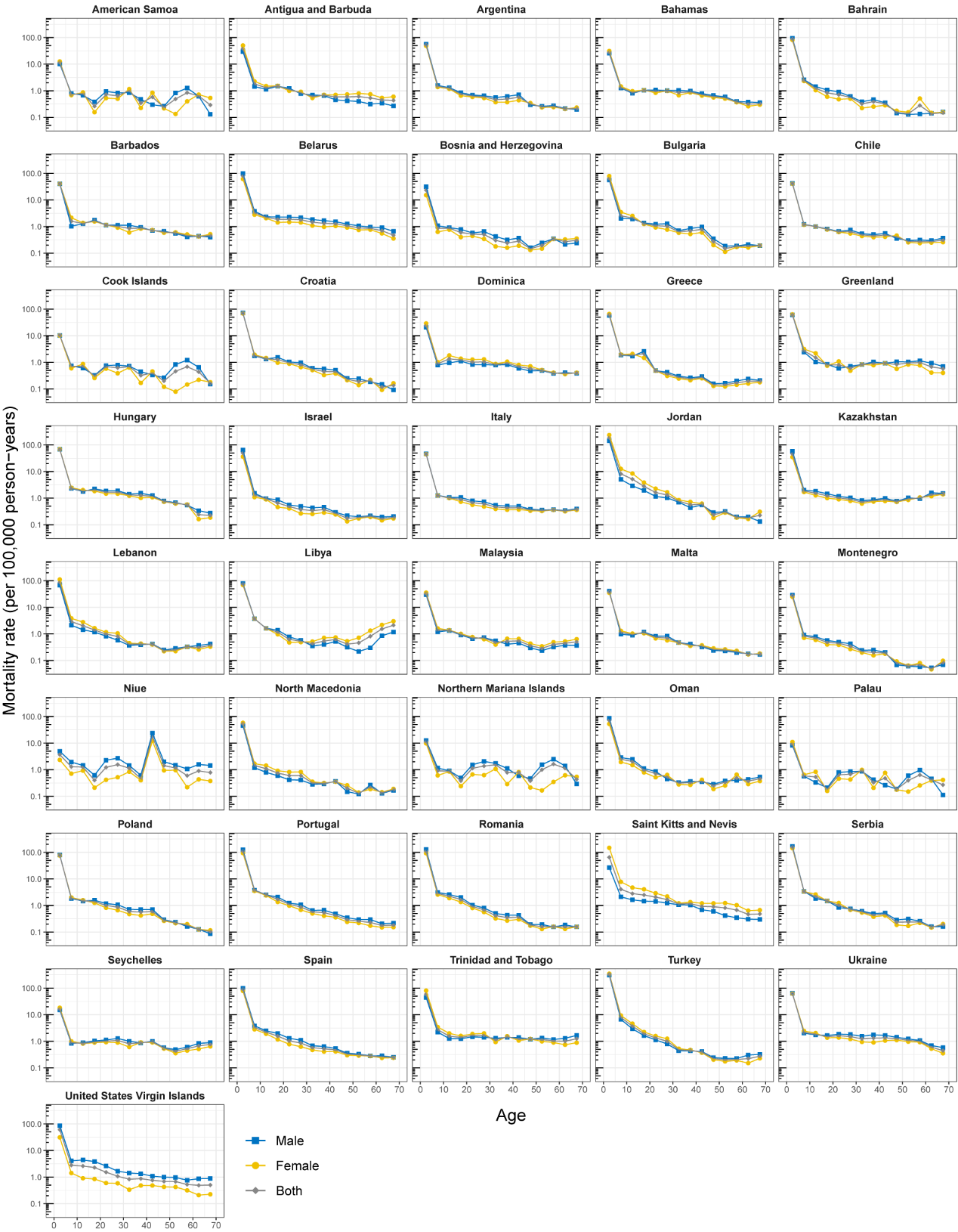


Age effects indicate age-associated natural history and are shown by the fitted longitudinal age curves of mortality (per 100000 person-years) adjusted for period deviations, with the dots and shaded areas denoting mortality rates with 95% CIs. SDI=Socio-demographic Index.

**Figure S15. Age effects on congenital heart disease mortality in middle SDI countries**


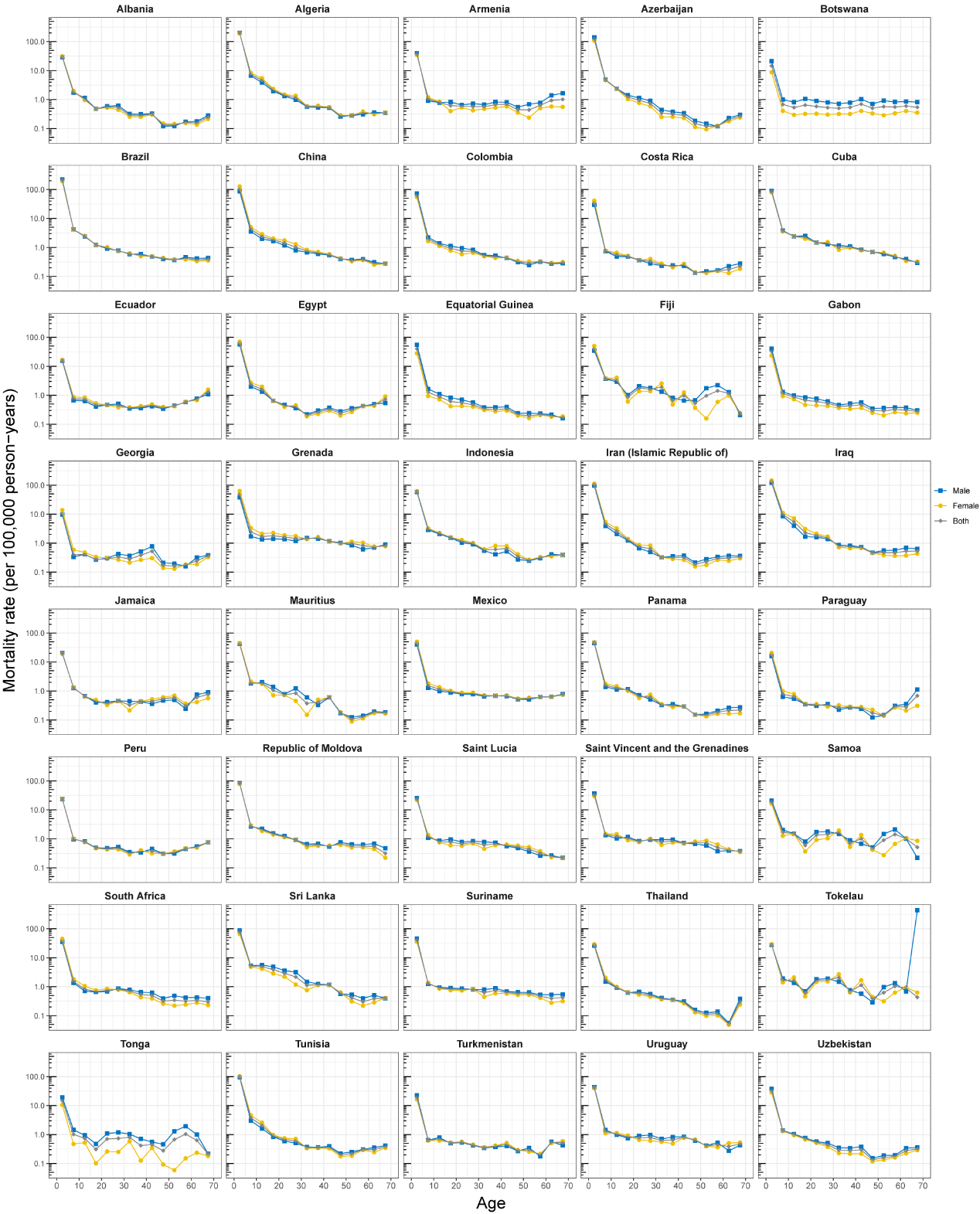


Age effects indicate age-associated natural history and are shown by the fitted longitudinal age curves of mortality (per 100000 person-years) adjusted for period deviations, with the dots and shaded areas denoting mortality rates with 95% CIs. SDI=Socio-demographic Index.

**Figure S16. Age effects on congenital heart disease mortality in low-middle SDI countries**


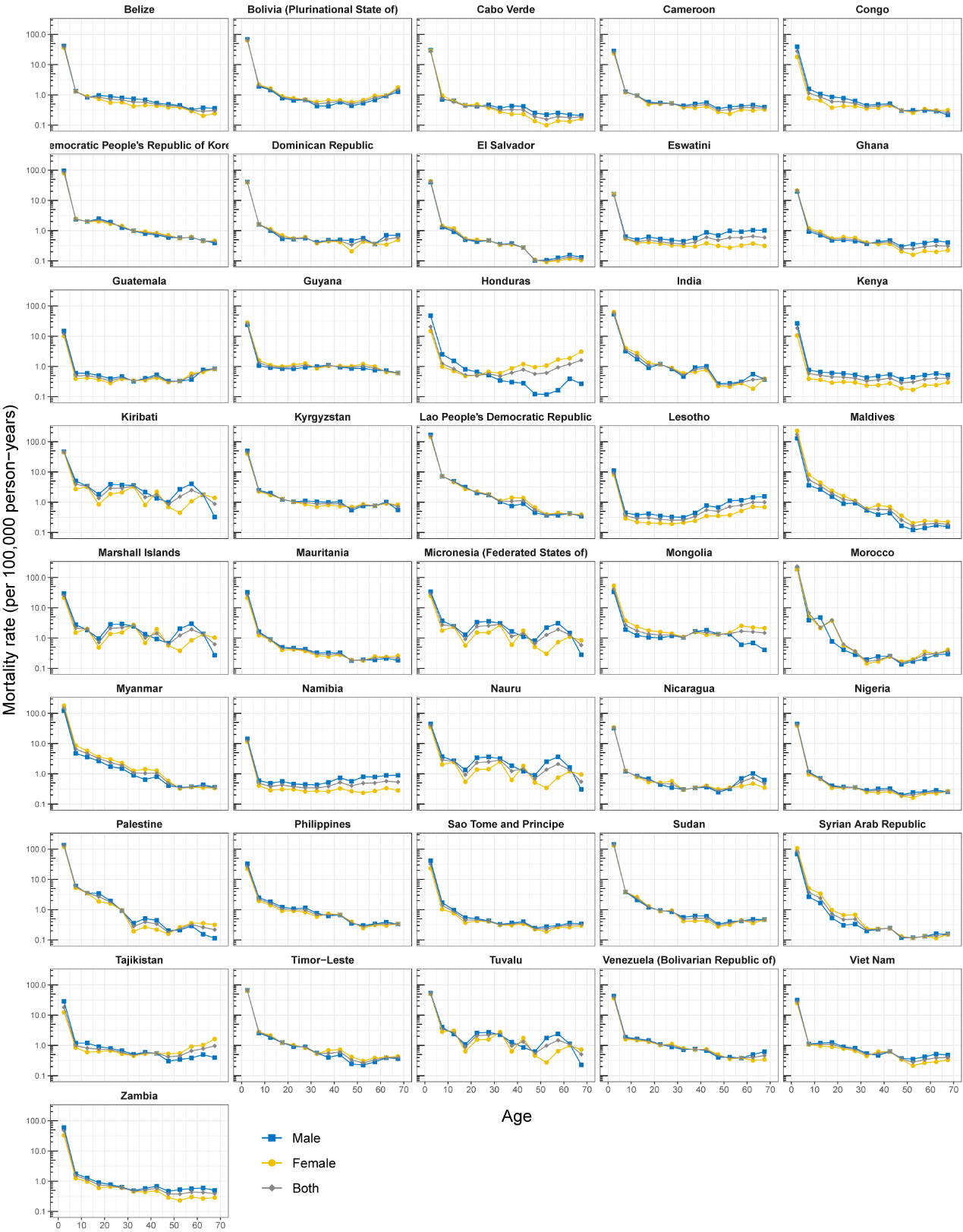


Age effects indicate age-associated natural history and are shown by the fitted longitudinal age curves of mortality (per 100000 person-years) adjusted for period deviations, with the dots and shaded areas denoting mortality rates with 95% CIs. SDI=Socio-demographic Index.

**Figure S17. Age effects on congenital heart disease mortality in low-SDI countries**


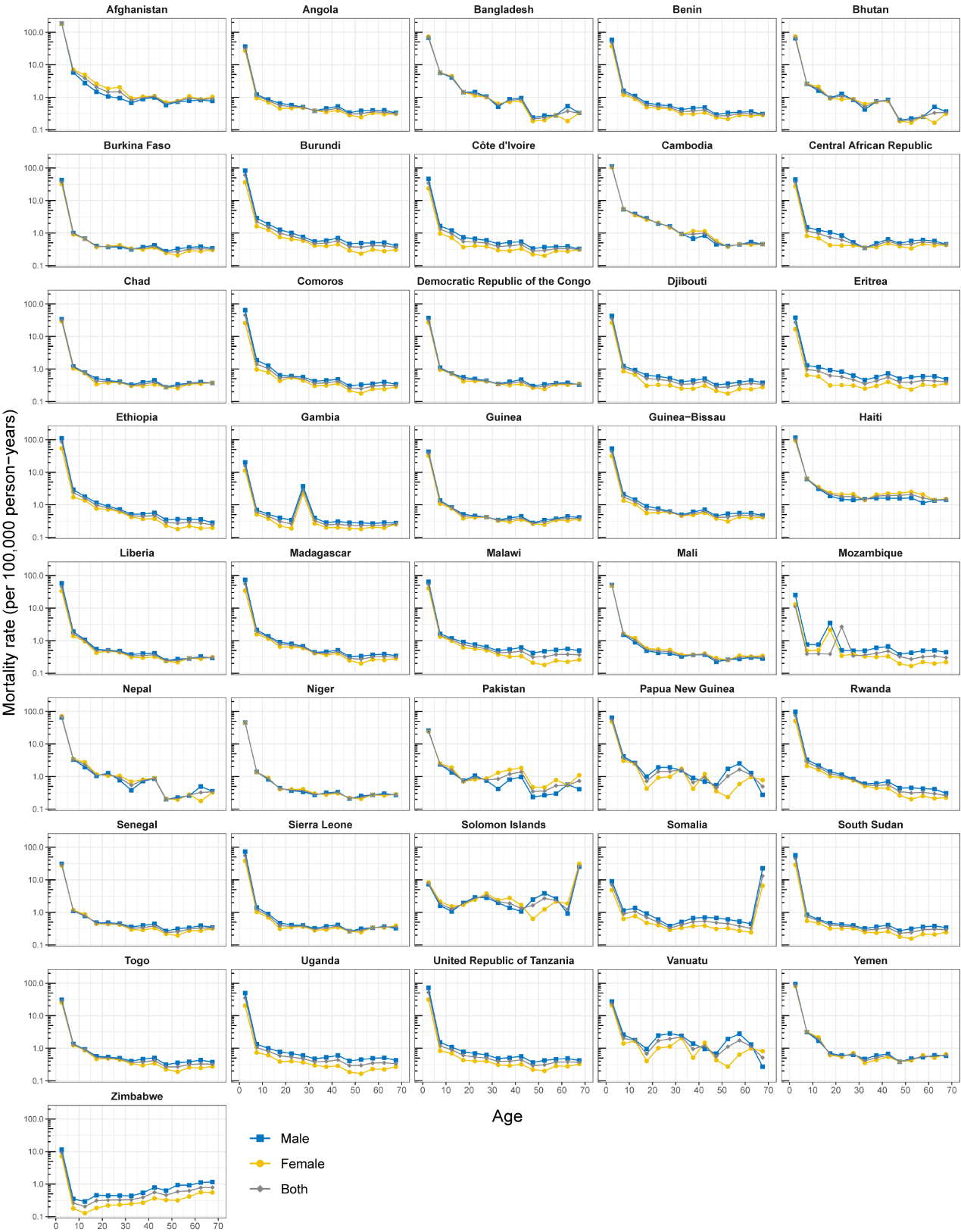


Age effects indicate age-associated natural history and are shown by the fitted longitudinal age curves of mortality (per 100000 person-years) adjusted for period deviations, with the dots and shaded areas denoting mortality rates with 95% CIs. SDI=Socio-demographic Index.

**Figure S18. Period effects on congenital heart disease mortality in high-SDI countries**


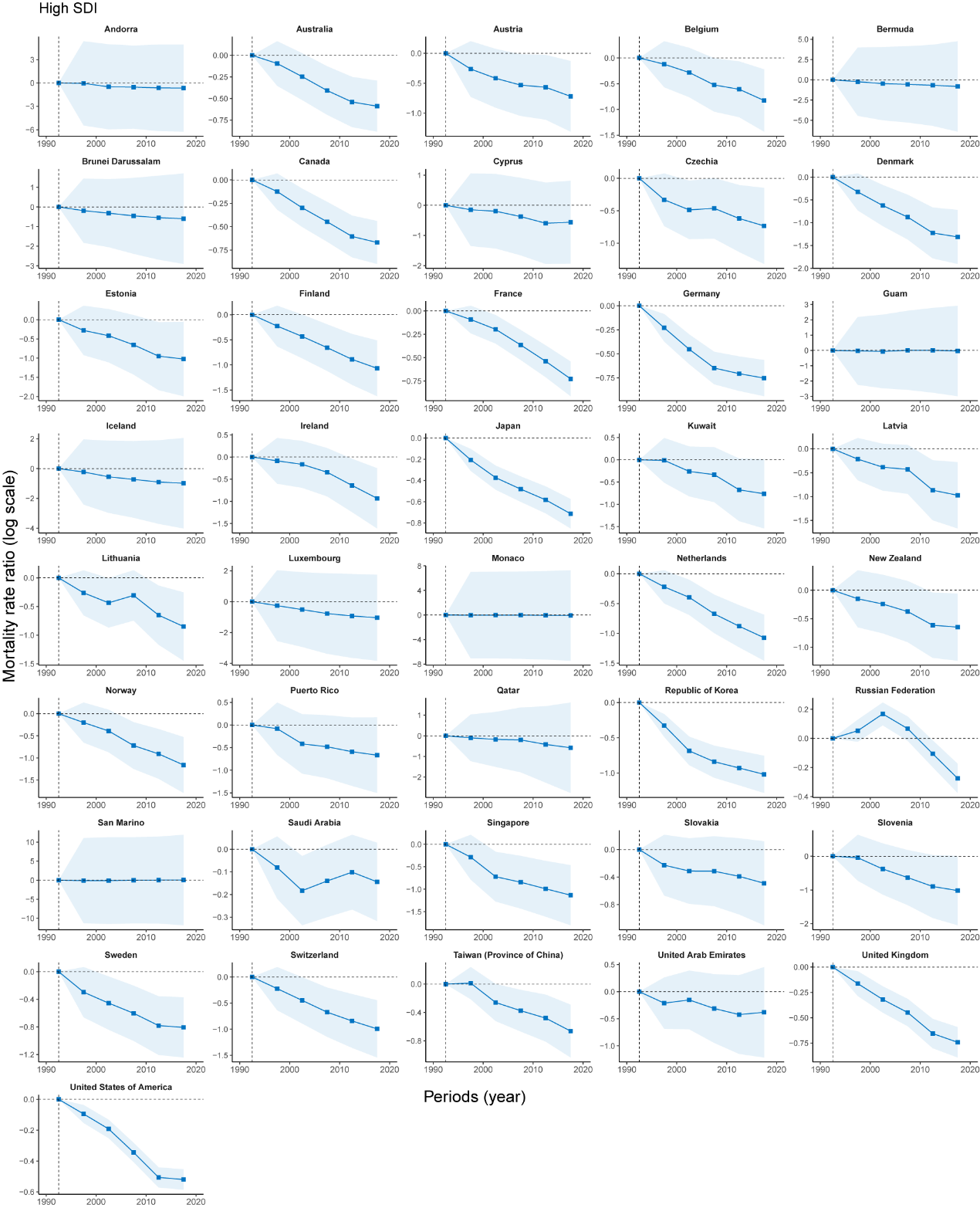


Period effects are shown by the relative risk of mortality (mortality rate ratio) for each period from 1990-1994 to 2015-2019, with the dots and shaded areas representing rate ratios and 95% CIs for a given period relative to the referent period (1990-1994). SDI=Socio-demographic Index.

**Figure S19. Period effects on congenital heart disease mortality in high-middle SDI countries**


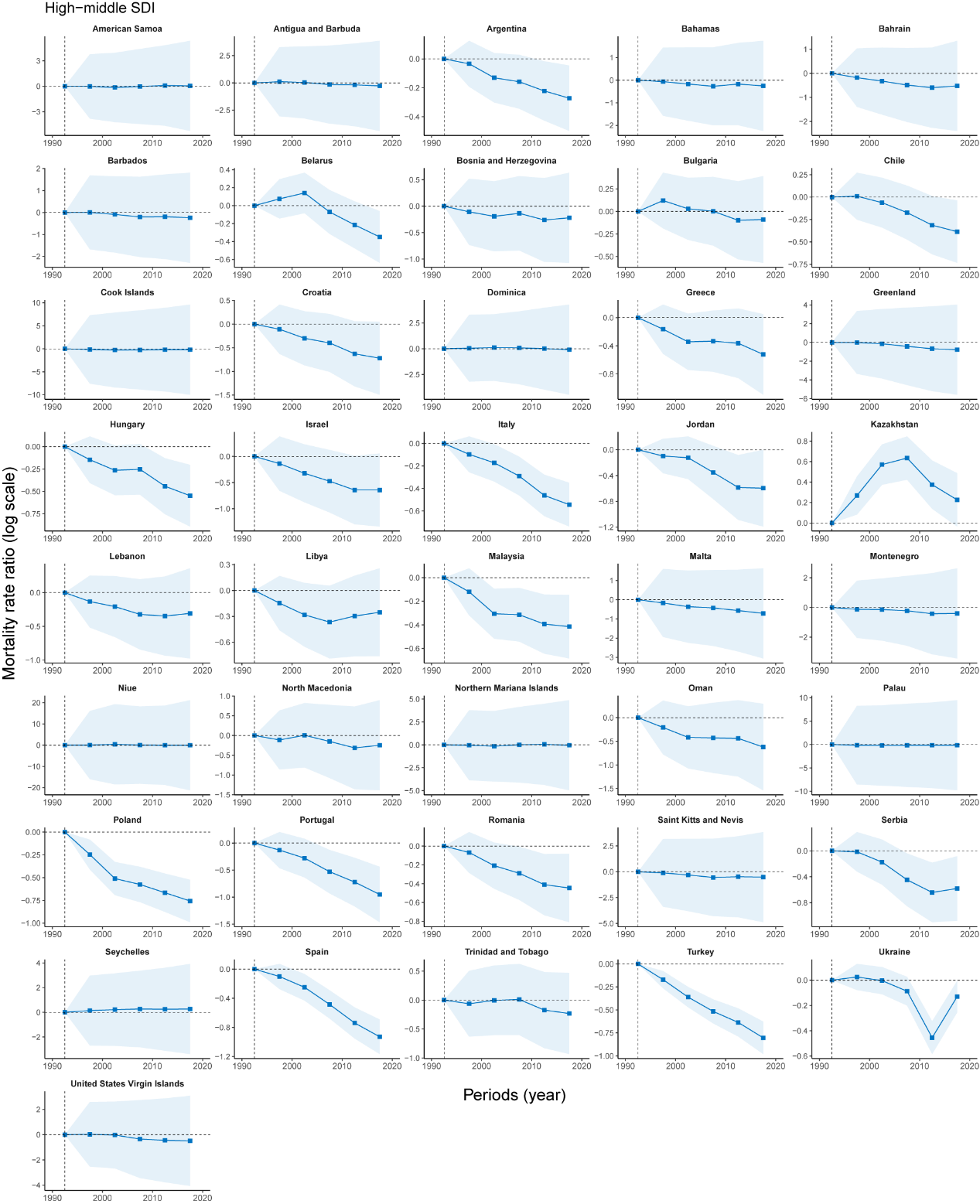


Period effects are shown by the relative risk of mortality (mortality rate ratio) for each period from 1990-1994 to 2015-2019, with the dots and shaded areas representing rate ratios and 95% CIs for a given period relative to the referent period (1990-1994). SDI=Socio-demographic Index.

**Figure S20. Period effects on congenital heart disease mortality in middle-SDI countries**


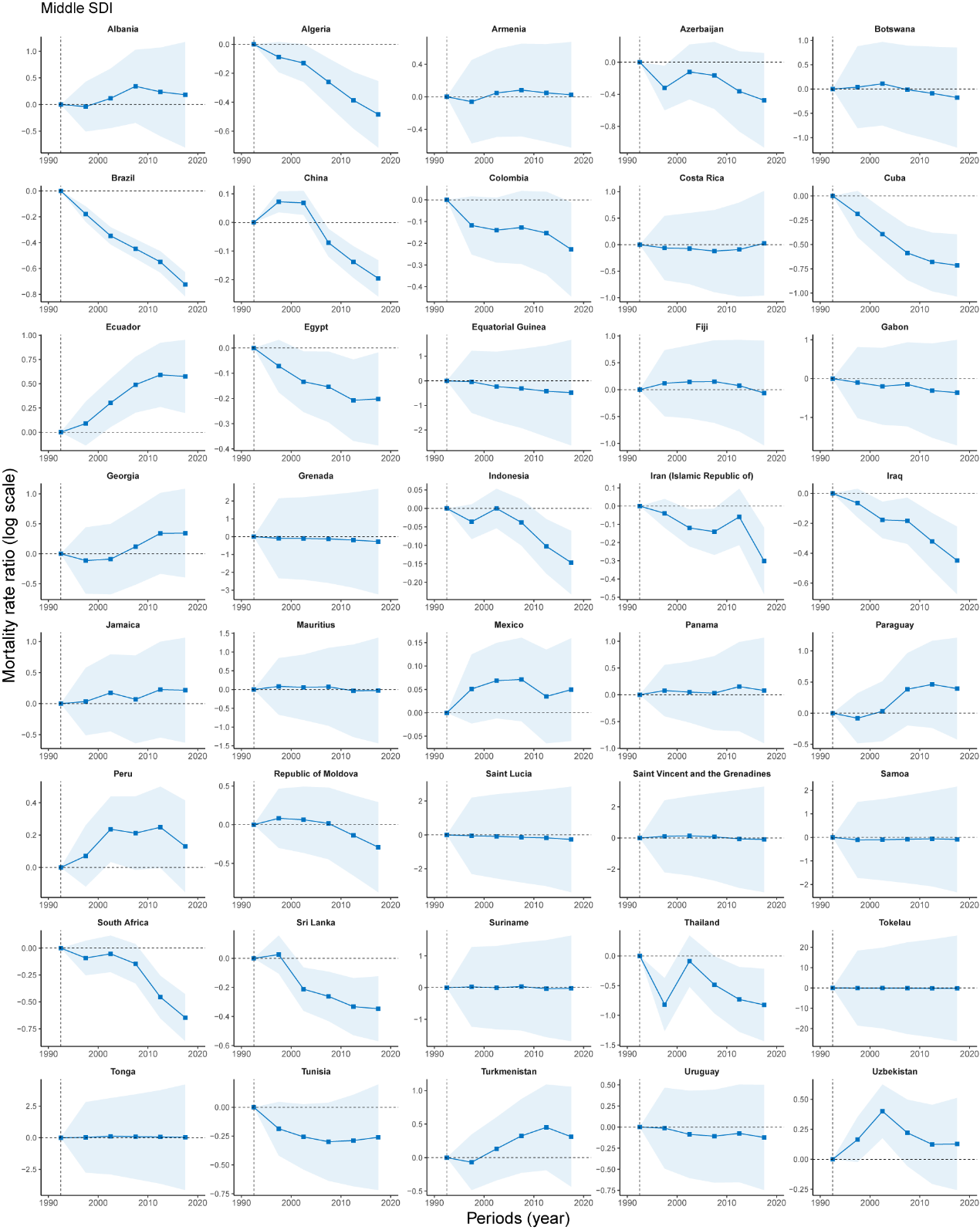


Period effects are shown by the relative risk of mortality (mortality rate ratio) for each period from 1990-1994 to 2015-2019, with the dots and shaded areas representing rate ratios and 95% CIs for a given period relative to the referent period (1990-1994). SDI=Socio-demographic Index.

**Figure S21. Period effects on congenital heart disease mortality in low-middle SDI countries**


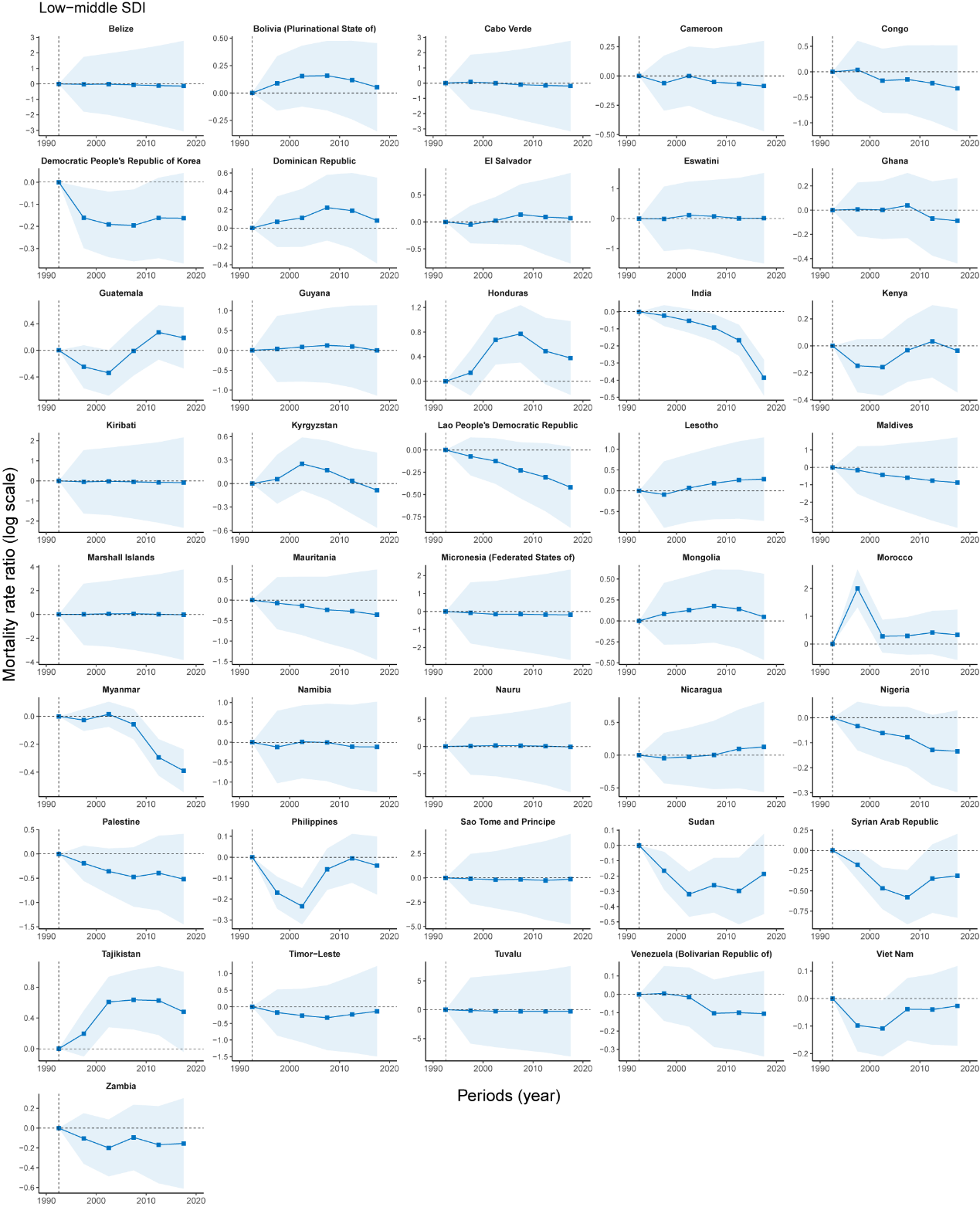


Period effects are shown by the relative risk of mortality (mortality rate ratio) for each period from 1990-1994 to 2015-2019, with the dots and shaded areas representing rate ratios and 95% CIs for a given period relative to the referent period (1990-1994). SDI=Socio-demographic Index.

**Figure S22. Period effects on congenital heart disease mortality in low-SDI countries**


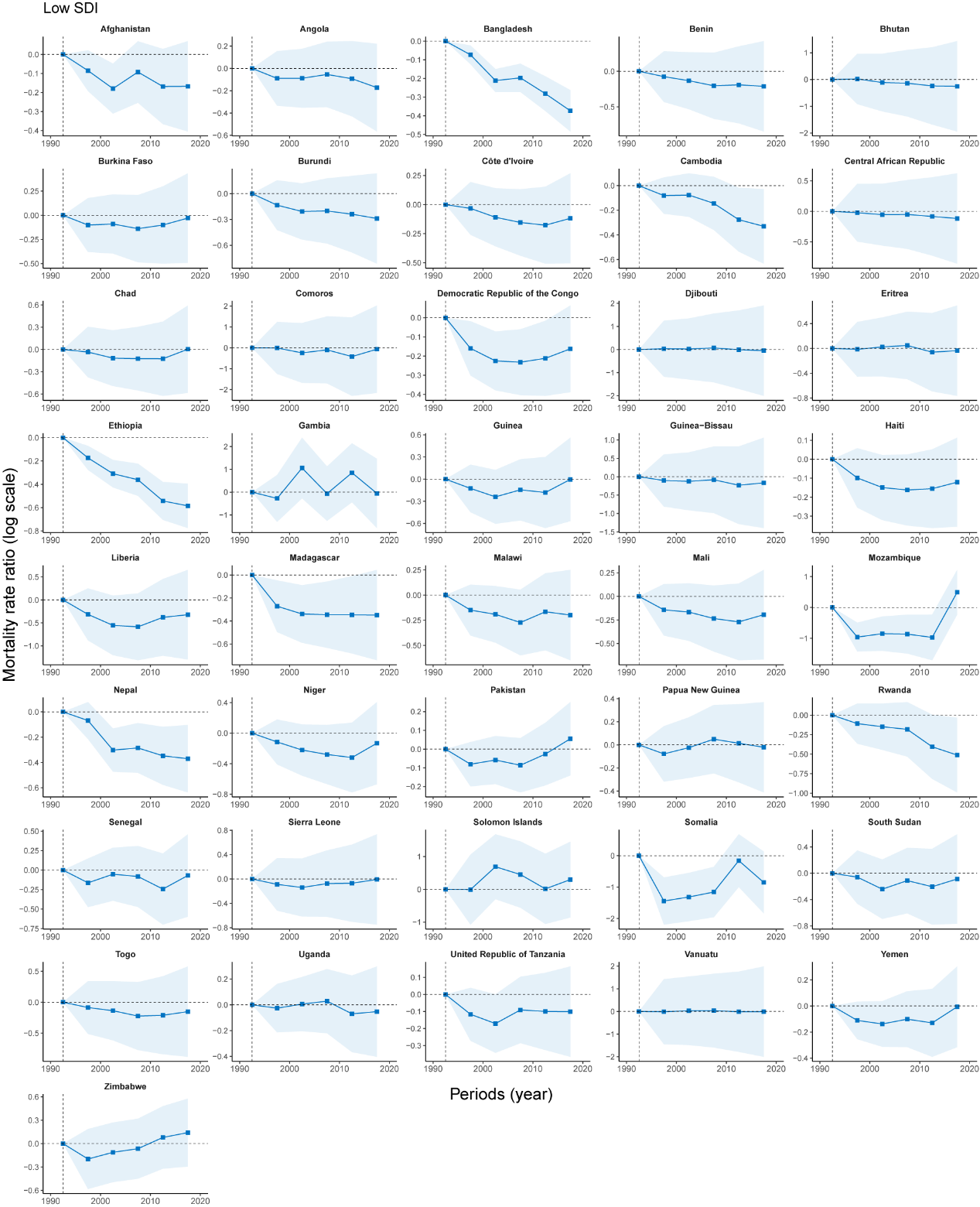


Period effects are shown by the relative risk of mortality (mortality rate ratio) for each period from 1990-1994 to 2015-2019, with the dots and shaded areas representing rate ratios and 95% CIs for a given period relative to the referent period (1990-1994). SDI=Socio-demographic Index.

**Figure S23. Cohort effects on congenital heart disease mortality in high-SDI countries**


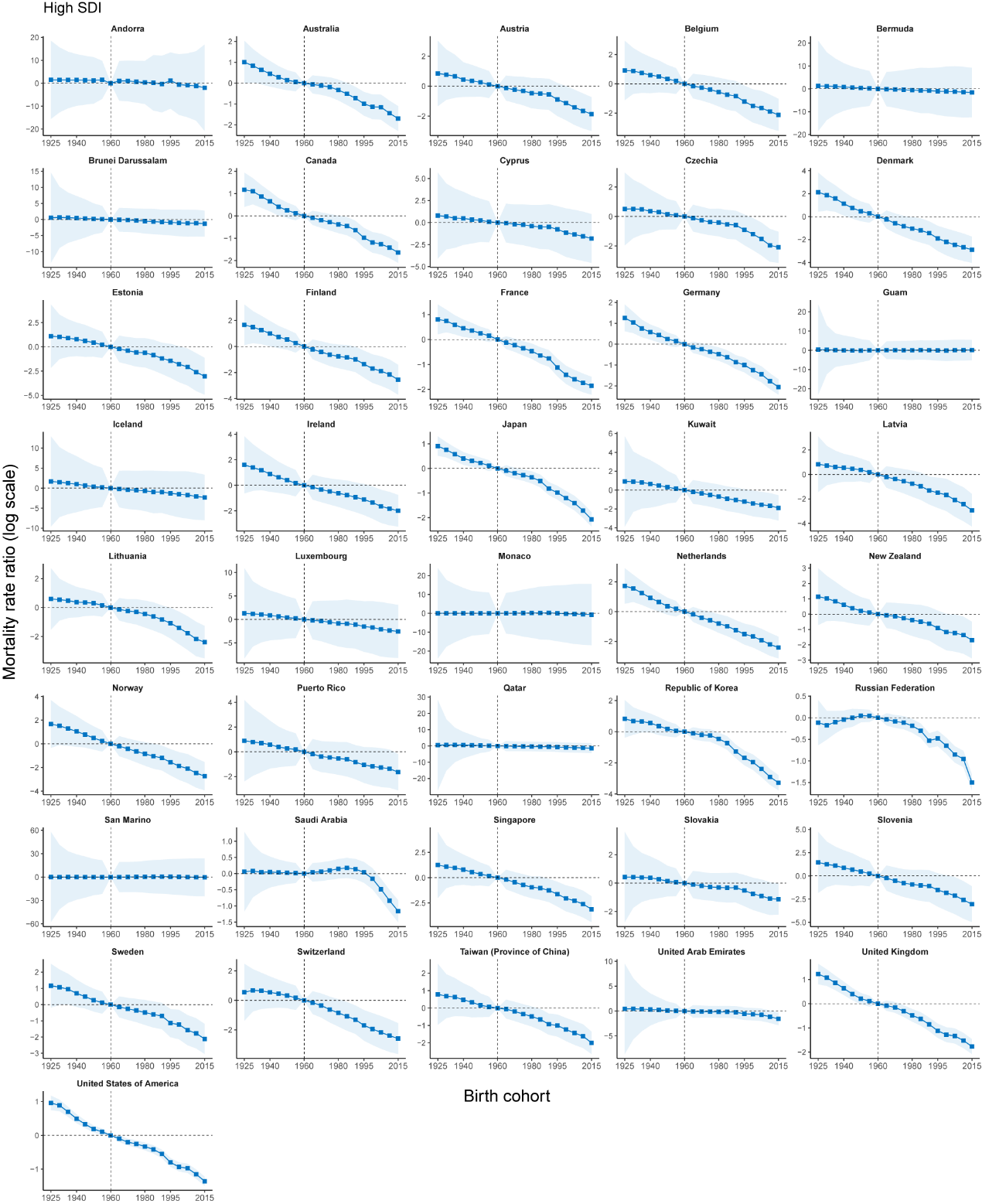


Cohort effects are shown by the relative risk of mortality (mortality rate ratio) for each birth cohort from 1925 to 2015, with the dots and shaded areas represent rate ratios and 95% CIs for a given cohort relative to the referent 1960 cohort. SDI=Socio-demographic Index.

**Figure S24. Cohort effects on congenital heart disease mortality in high-middle SDI countries**


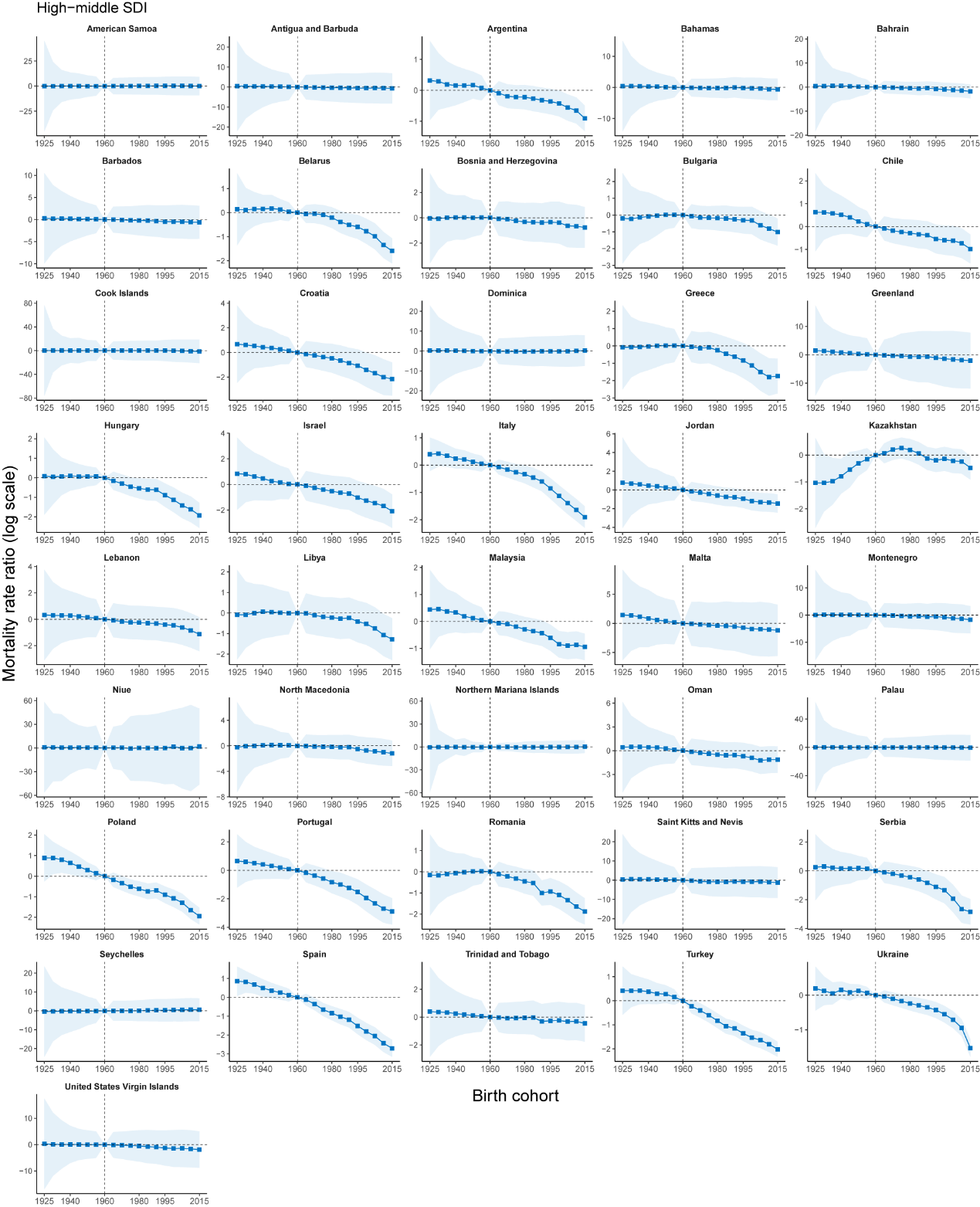


Cohort effects are shown by the relative risk of mortality (mortality rate ratio) for each birth cohort from 1925 to 2015, with the dots and shaded areas represent rate ratios and 95% CIs for a given cohort relative to the referent 1960 cohort. SDI=Socio-demographic Index.

**Figure S25. Cohort effects on congenital heart disease mortality in middle-SDI countries**


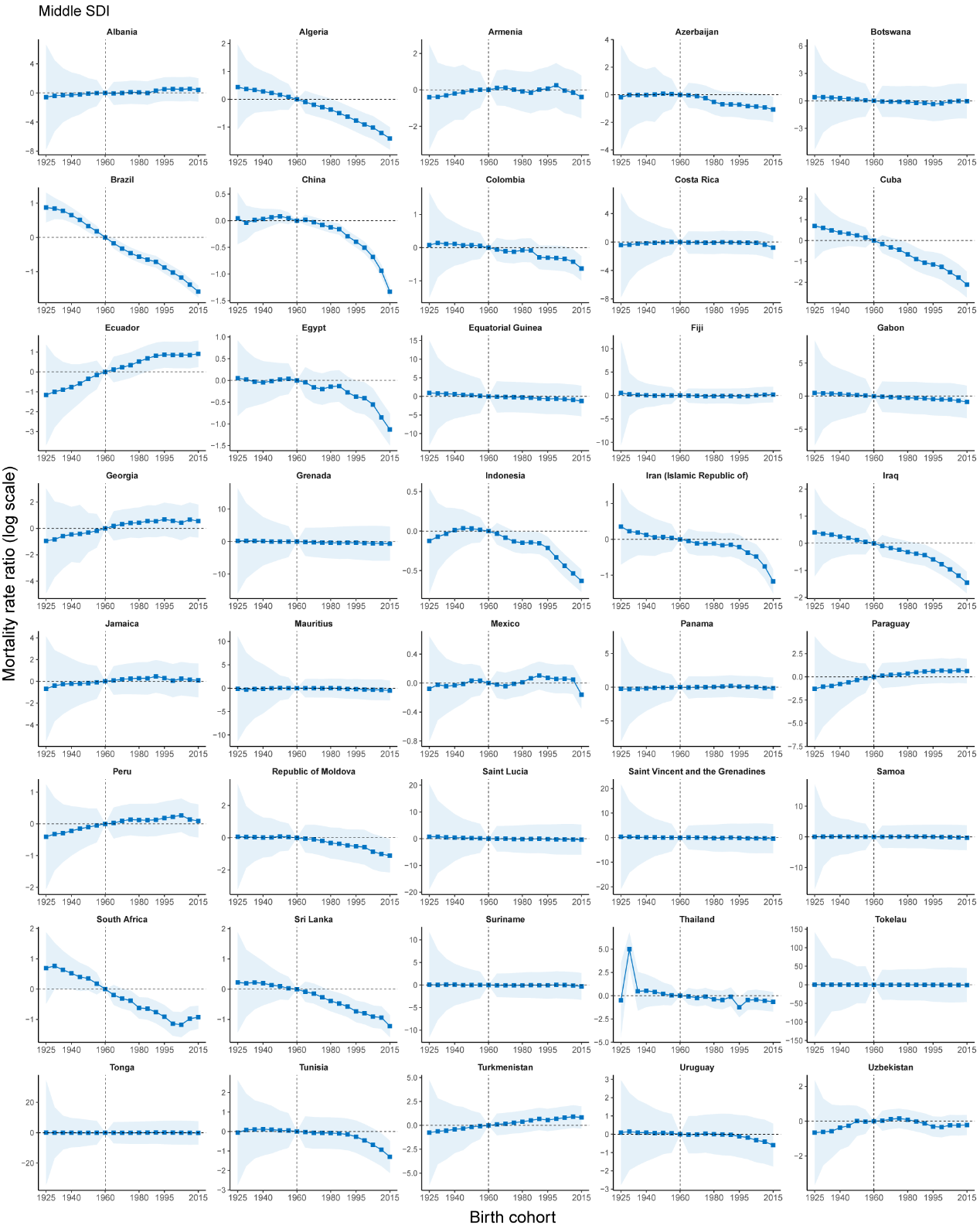


Cohort effects are shown by the relative risk of mortality (mortality rate ratio) for each birth cohort from 1925 to 2015, with the dots and shaded areas represent rate ratios and 95% CIs for a given cohort relative to the referent 1960 cohort. SDI=Socio-demographic Index.

**Figure S26. Cohort effects on congenital heart disease mortality in low-middle SDI countries**


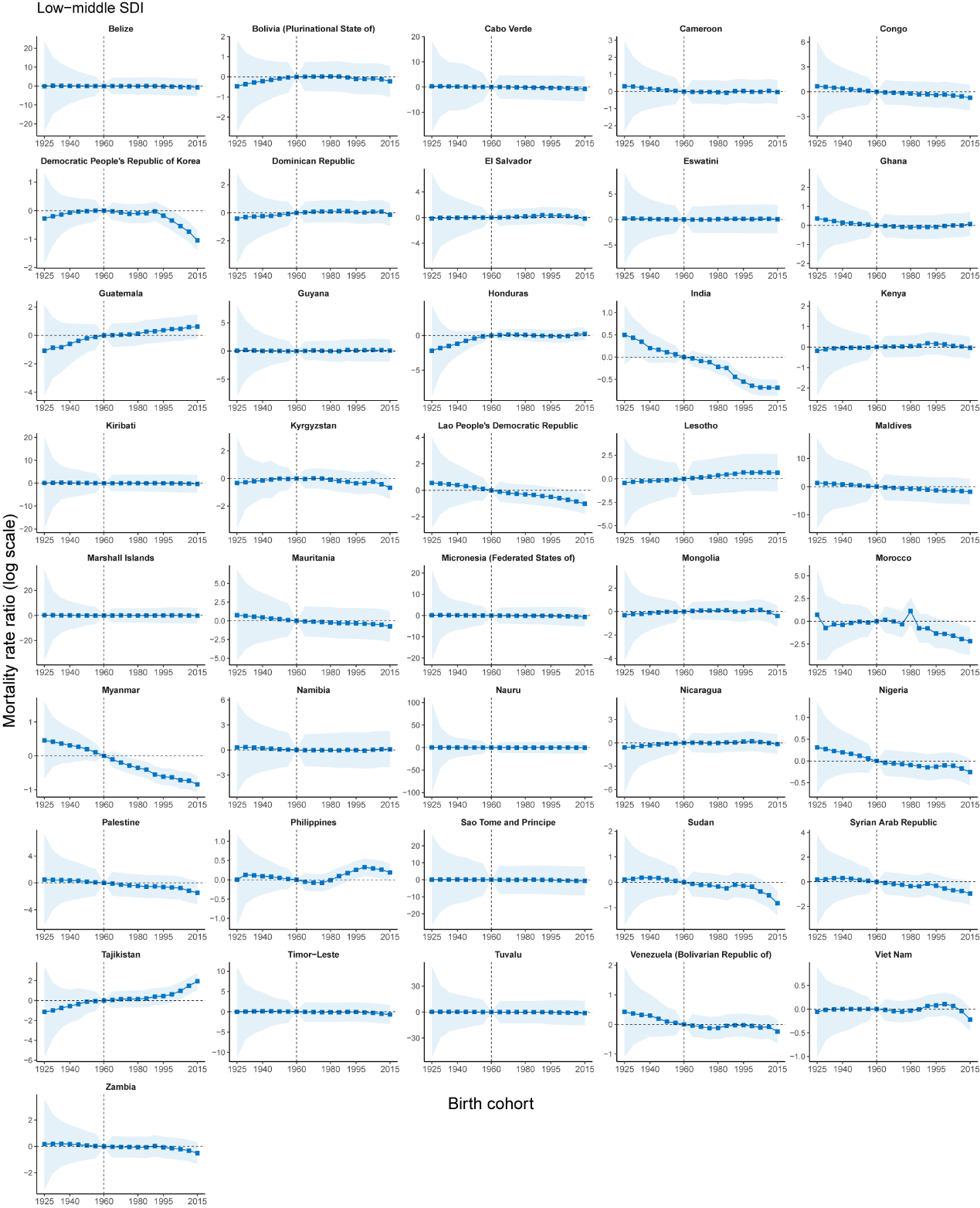


Cohort effects are shown by the relative risk of mortality (mortality rate ratio) for each birth cohort from 1925 to 2015, with the dots and shaded areas represent rate ratios and 95% CIs for a given cohort relative to the referent 1960 cohort. SDI=Socio-demographic Index.

**Figure S27. Cohort effects on congenital heart disease mortality in low-SDI countries**


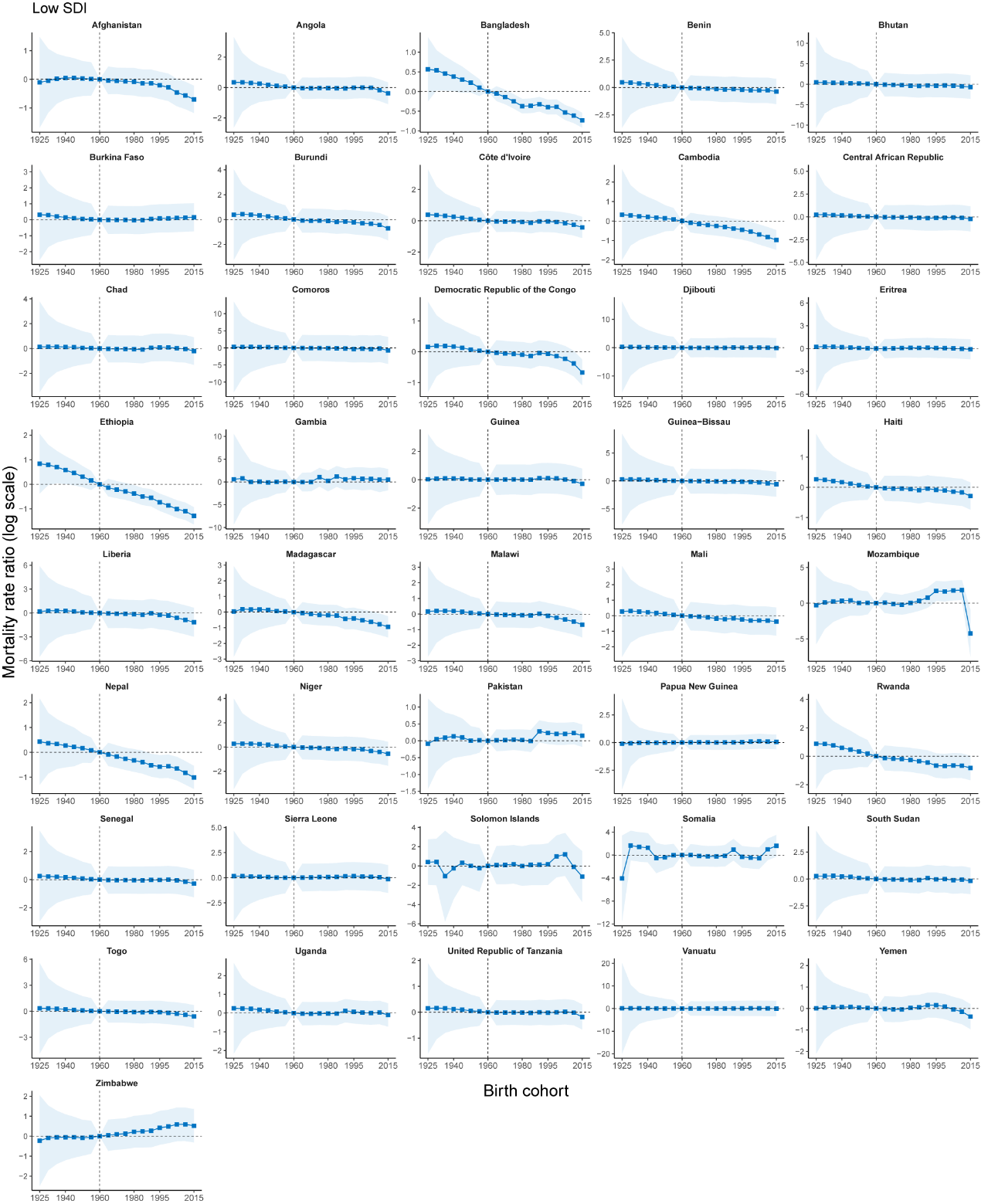


Cohort effects are shown by the relative risk of mortality (mortality rate ratio) for each birth cohort from 1925 to 2015, with the dots and shaded areas represent rate ratios and 95% CIs for a given cohort relative to the referent 1960 cohort. SDI=Socio-demographic Index.
